# Pan-cancer efficacy of pralsetinib in patients with *RET* fusion–positive solid tumors from the phase 1/2 ARROW trial

**DOI:** 10.1038/s41591-022-01931-y

**Published:** 2022-08-12

**Authors:** Vivek Subbiah, Philippe A. Cassier, Salvatore Siena, Elena Garralda, Luis Paz-Ares, Pilar Garrido, Ernest Nadal, Jacqueline Vuky, Gilberto Lopes, Gregory P. Kalemkerian, Daniel W. Bowles, Mahesh Seetharam, Jianhua Chang, Hui Zhang, Jennifer Green, Alena Zalutskaya, Martin Schuler, Yun Fan, Giuseppe Curigliano

**Affiliations:** 1 The University of Texas MD Anderson Cancer Center, Houston, TX USA; 2 Centre Léon Bérard, Lyon, France; 3 Department of Oncology and Hemato-Oncology, Università degli Studi di Milano (La Statale) and Niguarda Cancer Center, Grande Ospedale Metropolitano Niguarda, Milan, Italy; 4 Vall d’Hebron Institute of Oncology, Vall d’Hebron Hospital, Barcelona, Spain; 5 Hospital Universitario 12 de Octubre, CNIO-H12o Lung Cancer Unit, Ciberonc and Complutense University, Madrid, Spain; 6 IRYCIS Hospital Universitario Ramón y Cajal, Ciberonc and Alcalá University, Madrid, Spain; 7 Catalan Institute of Oncology, L’Hospitalet, Spain; 8 Oregon Health & Science University, Portland, OR USA; 9 University of Miami Health System, Miami, FL USA; 10 University of Michigan, Ann Arbor, MI USA; 11 University of Colorado, Aurora, CO USA; 12 Mayo Clinic, Phoenix, AZ USA; 13 Cancer Hospital Chinese Academy of Medical Sciences, Shenzhen, China; 14 Blueprint Medicines Corporation, Cambridge, MA USA; 15 West German Cancer Center Essen, University Hospital Essen, Essen, Germany, and German Cancer Consortium (DKTK), Essen, Germany; 16 Department of Medical Oncology, Zhejiang Cancer Hospital, Hangzhou City, China; 17 Department of Oncology and Hemato-Oncology, University of Milan, Milan, Italy; 18 Istituto Europeo di Oncologia, IRCCS, Milan, Italy

**Keywords:** Target validation, Prognostic markers, Pharmacodynamics

## Abstract

Oncogenic *RET* fusions occur in diverse cancers. Pralsetinib is a potent, selective inhibitor of RET receptor tyrosine kinase. ARROW (NCT03037385, ongoing) was designed to evaluate pralsetinib efficacy and safety in patients with advanced *RET*-altered solid tumors. Twenty-nine patients with 12 different *RET* fusion–positive solid tumor types, excluding non-small-cell lung cancer and thyroid cancer, who had previously received or were not candidates for standard therapies, were enrolled. The most common *RET* fusion partners in 23 efficacy-evaluable patients were *CCDC6* (26%), *KIF5B* (26%) and *NCOA4* (13%). Overall response rate, the primary endpoint, was 57% (95% confidence interval, 35–77) among these patients. Responses were observed regardless of tumor type or *RET* fusion partner. Median duration of response, progression-free survival and overall survival were 12 months, 7 months and 14 months, respectively. The most common grade ≥3 treatment-related adverse events were neutropenia (31%) and anemia (14%). These data validate RET as a tissue-agnostic target with sensitivity to RET inhibition, indicating pralsetinib’s potential as a well-tolerated treatment option with rapid, robust and durable anti-tumor activity in patients with diverse *RET* fusion–positive solid tumors.

## Abstract (web summary)

Results from the precision oncology ARROW trial identify the RET receptor tyrosine kinase as a tissue-agnostic target and the drug pralsetinib’s potential as a well-tolerated treatment option with rapid, robust and durable anti-tumor activity in patients with diverse RET fusion–positive solid tumors.

## Main

The RET proto-oncogene (*RET*) encodes a transmembrane receptor tyrosine kinase (proto-oncogene tyrosine protein kinase receptor RET) that has a physiological role in the embryonic development of the nervous system and the kidneys^1,2^. *RET* fusions and mutations induce oncogenic transformation, leading to the aberrant activation of RET receptor tyrosine kinase^3^. *RET* fusions can be found in 1–2% of non-small-cell lung cancers (NSCLCs), approximately 20% of papillary thyroid cancers and <1% of many other solid tumors, including ovarian, pancreatic, salivary and colorectal cancers^4–8^.

Pralsetinib (formerly known as BLU-667, Blueprint Medicines Corporation) is a selective RET inhibitor that potently targets RET kinases, including RET fusion proteins. The recommended phase 2 dose of 400 mg once daily (QD) orally administered pralsetinib was determined in phase 1 of the ARROW study^9^. Pralsetinib has low affinity for off-target kinases. In a biochemical assay, pralsetinib was 88-fold more selective for RET than for vascular endothelial growth factor receptor 2 (VEGFR2), a tyrosine kinase receptor that is targeted by multi-kinase inhibitors such as cabozantinib and vandetanib^2^. Based on the results from the registrational phase 1/2 ARROW study (NCT03037385)^10,11^, pralsetinib was approved in several countries globally, including the United States, for treatment of metastatic *RET* fusion–positive NSCLC, advanced or metastatic *RET*-mutant medullary thyroid cancer and *RET* fusion–positive thyroid cancer^12^, as well as in the European Union for treatment of advanced *RET* fusion–positive NSCLC^13^.

Pre-clinical and early clinical evidence suggests that *RET* fusions lead to oncogene addiction across tumor types and have the potential to be targeted by selective RET inhibition. Recent tumor-agnostic drug approvals have demonstrated that patients can benefit from select molecularly targeted therapies irrespective of tumor histology^14–18^. These landmark approvals have heralded the era of precision oncology for tissue-agnostic targets. Since the approvals of pralsetinib and selpercatinib in NSCLC and thyroid cancer^12,19^, biomarker testing for *RET* alterations is recommended in treatment guidelines for patients with these tumor types^20–22^. However, this is not standard of care across all disease indications where *RET* alterations are recognized as oncogenic drivers^23,24^.

ARROW is a phase 1/2 study of the highly selective RET inhibitor pralsetinib in patients with medullary thyroid cancer, *RET*-altered NSCLC and other *RET*-altered solid tumors. Efficacy and safety of pralsetinib in patients with *RET*-altered NSCLC and thyroid cancer from the ARROW study were previously reported^10,11^. After recent approvals of pralsetinib in patients with *RET*-altered NSCLC and thyroid cancers and respective publications of these data, here we present interim data on the efficacy and safety of pralsetinib in prospectively identified patients with diverse *RET* fusion–positive tumors.

## Results

### Patients

**Fig. 1:**
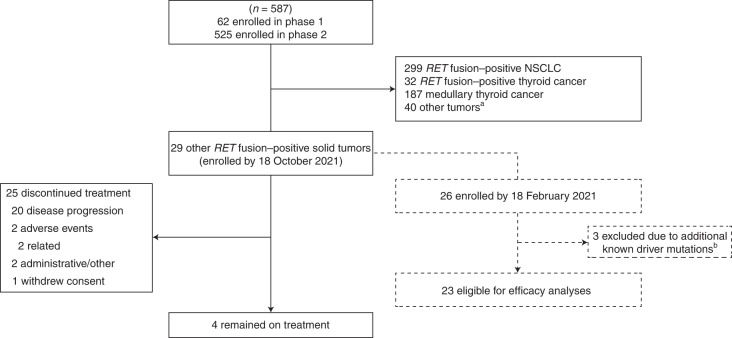
Patient disposition. A flowchart that illustrates enrollment of patients with *RET* fusion–positive solid tumors in the safety (*n* = 29) and efficacy-evaluable (*n* = 23) populations in the context of the overall study population of 587 patients, as well as the status of these patients at the data cutoff. ^a^Other *RET*-mutant tumors (*n* = 15), no or unknown *RET* status (*n* = 2) and prior treatment with a RET inhibitor (*n* = 23). ^b^Three patients (two with colon cancer and one with cholangiocarcinoma) had additional driver mutations (*KRAS*, *PIK3CB* and *BRAF*).

**Extended Data Fig. 1:**
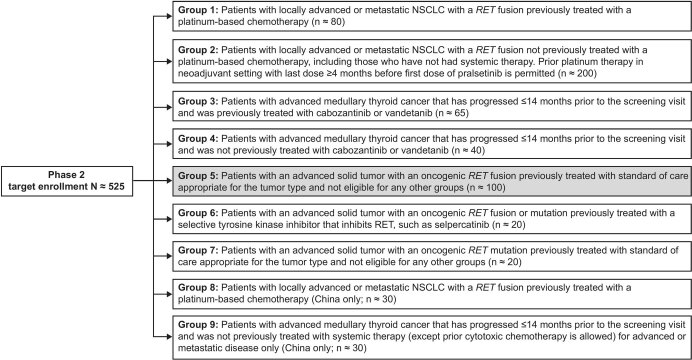
Prior therapies at baseline. All Ns are target enrollment. Safety and efficacy analyses presented herein were based on the shaded group (5).

Between 17 March 2017 and the data cutoff date of 18 October 2021, 587 patients were enrolled across all groups (Fig. 1 and Extended Data Fig. 1). Of these, 29 patients had *RET* fusion–positive solid tumors, excluding *RET* fusion–positive NSCLC or thyroid cancer, and were included in the safety population presented here. In total, 28 patients (96%) received a starting dose of pralsetinib 400 mg QD, and one patient (4%) received a starting dose of pralsetinib 200 mg/100 mg twice daily but transitioned to 400 mg QD after 3.4 months; the latter patient was the only patient included from the dose-escalation phase of the ARROW trial. At the data cutoff date, four patients (14%) remained on treatment, and 25 patients (86%) had discontinued treatment for the following reasons: disease progression (20 patients (69%)); administrative/other (two patients (7%)); adverse events (AEs) (two patients (7%), of which both were treatment-related—grade 3 thrombocytopenia and grade 3 neutropenia); and withdrew consent (one patient (3%)).

**Extended Data Fig. 2:**
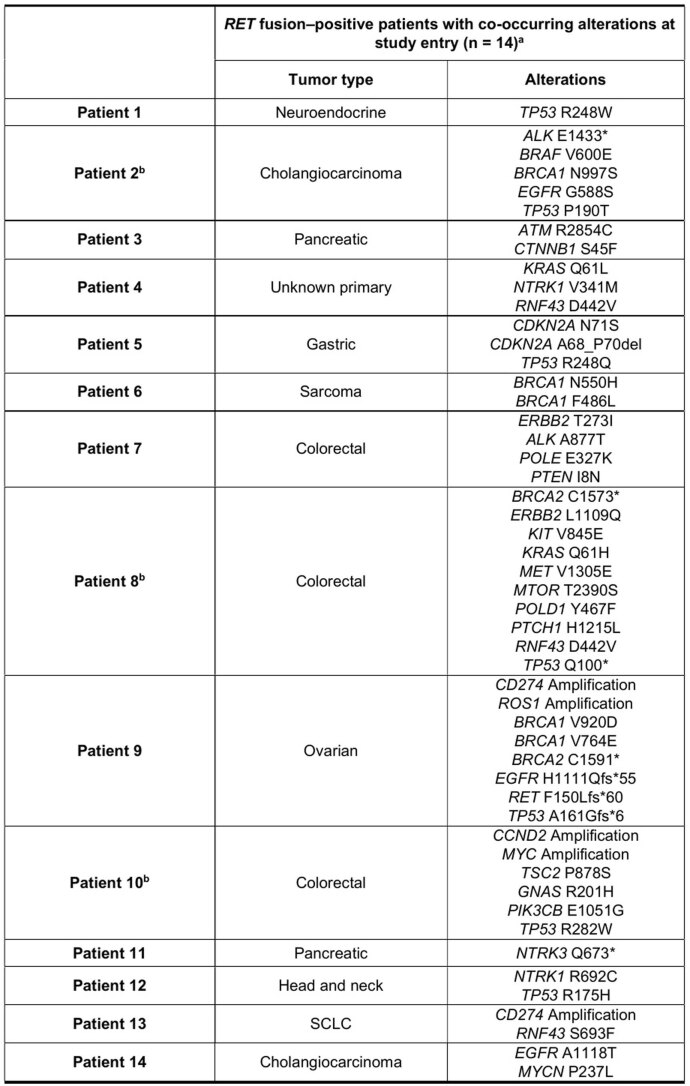
Co-occurring alterations in patients with *RET* fusions in the ARROW trial at study entry. ^a^12 patients from the efficacy population did not have any genomics carried out except CRF before the first treatment and were therefore not included. ^b^Two patients with colorectal cancer and one patient with cholangiocarcinoma were excluded from efficacy analyses due to additional co-occurring driver alterations of *PIK3CB* E1051G, *KRAS* Q61H and *BRAF* V600E, respectively. After enrollment, although the patients were allowed to continue on-study, considering concurrent activating events the sponsor later excluded these patients from the efficacy cohort as the concurrent drivers were adjudicated centrally.

**Extended Data Fig. 3:**
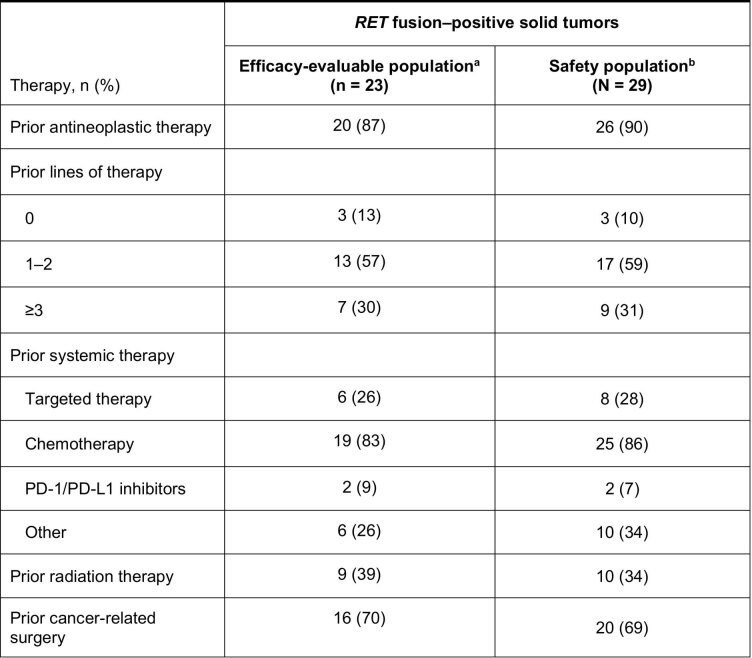
Phase 2 study design. ^a^Enrollment as of 18 February 2021 and data cutoff date 18 October 2021. ^b^Enrollment and data cutoff date as of 18 October 2021. PD-1, programmed death-1; PD-L1, programmed death ligand-1.

Twenty-six of the 29 patients in the safety population enrolled by the 18 October 2021 data cutoff, with *RET* fusion–positive solid tumors excluding *RET* fusion–positive NSCLC or thyroid cancer, were enrolled by the efficacy enrollment cutoff date of 18 February 2021. Fourteen of 26 patients were confirmed to have co-occurring alterations at study entry (Extended Data Fig. 2). *RET* fusions were identified as the only oncogenic driver in 23 patients (three patients had other oncogenic drivers in addition to *RET* and were excluded from the efficacy-evaluable population due to this pre-specified criterion). Among these 23 patients evaluable for efficacy, the median age was 53 years (range, 31–71); 14 patients (61%) were female; 20 (87%) patients had metastatic disease; and 20 (87%) patients had received prior therapies at baseline (Table 1 and Extended Data Fig. 3). The most common cancer diagnoses in the efficacy-evaluable population were pancreatic cancer (four patients (17%)), cholangiocarcinoma (three patients (13%)), neuroendocrine cancer (three patients (13%)), sarcoma (three patients (13%): malignant mesenchymal tumor (one patient (4%)), mixed sarcoma and adenocarcinoma (one patient (4%)), malignant isolated fibroma (one patient (4%))), head and neck cancer (two patients (9%): sweat gland cancer (one patient (4%)), salivary duct cancer (one patient (4%))), colorectal cancer (two patients (9%)) and small-cell lung cancer (SCLC) (two patients (9%)). Three patients had stage 3 disease, including one patient with stage 3 ovarian cancer who had received nine prior lines of therapy and one patient each with stage 3B gastric cancer or sarcoma who had both received one prior line of therapy. Two patients had not received prior systemic therapy, both of whom had stage 4 head and neck cancer.

**Table 1:** Patient demographics and baseline characteristics

Demographic/characteristic	*RET* fusion*–*positive solid tumors	
	Efficacy-evaluable population^a^ (*n* = 23)	Safety population^b^ (*n* = 29)
Median age (range), years	53 (31–71)	55 (25–75)
Sex, *n* (%)		
Female	14 (61)	18 (62)
Male	9 (39)	11 (38)
Race, *n* (%)		
White	15 (65)	20 (69)
Asian	7 (30)	8 (28)
Black	1 (4)	1 (3)
ECOG performance status, *n* (%)		
0	7 (30)	11 (38)
1	16 (70)	18 (62)
Tumor type, *n* (%)		
Pancreatic	4 (17)	5 (17)
Cholangiocarcinoma	3 (13)	4 (14)
Neuroendocrine	3 (13)	3 (10)
Sarcoma	3 (13)	3 (10)
Head and neck	2 (9)	2 (7)
Colorectal	2 (9)	5 (17)
SCLC	2 (9)	2 (7)
Unknown primary	1 (4)	1 (3)
Gastric	1 (4)	1 (3)
Ovarian	1 (4)	1 (3)
Thymic	1 (4)	1 (3)
CNS	0	1 (3)
History of CNS metastases, *n* (%)	6 (26)	7 (24)
TNM stage, *n* (%)		
III	3 (13)	4 (14)
IV	20 (87)	24 (83)
Unknown	0	1 (3)
*RET* fusion partner, *n* (%)		
*CCDC6*	6 (26)	9 (31)
*KIF5B*	6 (26)	6 (21)
*NCOA4*	3 (13)	4 (14)
Other^c^	5 (22)	6 (21)
Unknown	3 (13)	4 (14)
Median prior lines of therapy, *n* (range)	2 (1–9)	2 (1–9)

Baseline demographic and clinical characteristics of patients in the safety population (*n* = 29) and efficacy-evaluable population (*n* = 23) with *RET* fusion–positive solid tumors. Three patients in the safety population were not enrolled by the efficacy enrollment date of 18 February 2021, and an additional three patients had oncogenic drivers in addition to *RET* and were excluded from the efficacy-evaluable population. ^a^Enrollment as of 18 February 2021 and data cutoff date 18 October 2021. ^b^Enrollment and data cutoff date as of 18 October 2021. ^c^Includes *ANKRD26*, *MYH10*, *NUP93*, *SATB1*, *PRKG1* and *TRIM24*, and *TRIM33* and *JMJD1C*. CNS, central nervous system; ECOG, Eastern Cooperative Oncology Group; TNM, tumor, node, metastases.

*RET* fusions were identified by next-generation sequencing (NGS) in 16 patients (70%), by fluorescence in situ hybridization (FISH) in five patients (22%) and by GeneTrails Solid Tumor Fusion Panel and local NGS each in one patient (4%). Central circulating tumor DNA (ctDNA) analysis was also performed in patients for whom FISH was used, with the aim of identifying the *RET* fusion partners. The most common *RET* fusion partners were *CCDC6* (six patients (26%)), *KIF5B* (six patients (26%)) and *NCOA4* (three patients (13%)) (Table 1). None of the tumors in the patients with pancreatic cancer was identified to harbor *KRAS* mutations.

### Efficacy

**Fig. 2:**
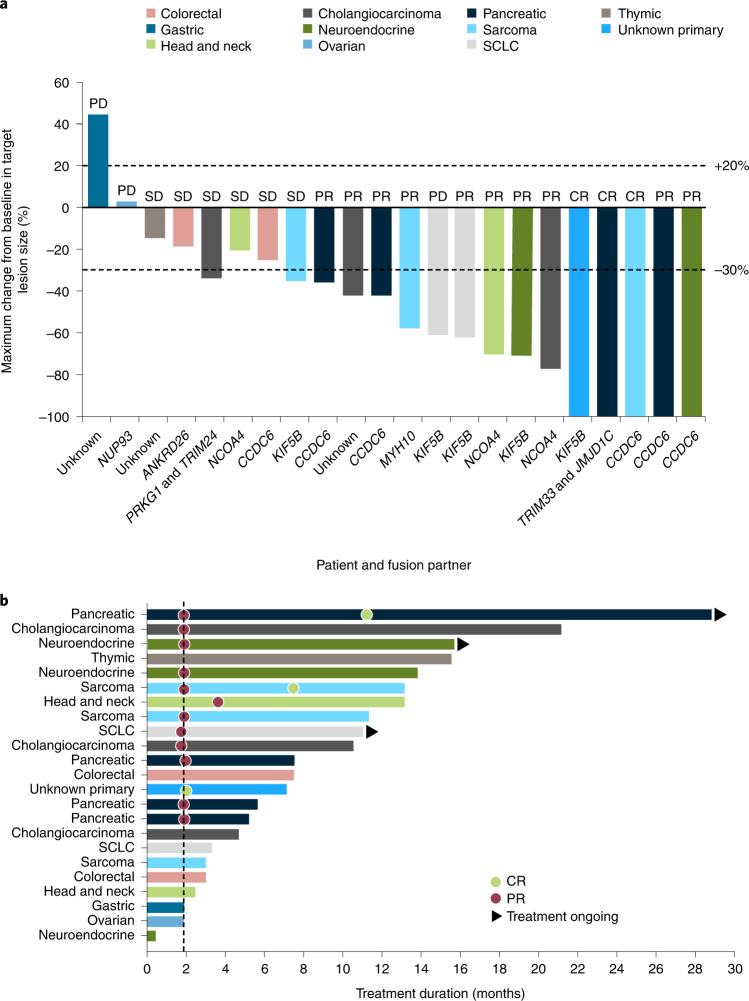
Individual tumor response and treatment duration waterfall and swimlane plots for the efficacy-evaluable population. In 23 patients eligible for efficacy analyses: **a**, tumor response by BICR and maximum change from baseline in target lesion size, showing each patient’s tumor type and *RET* fusion partner; **b**, treatment duration, indicating the corresponding tumor type and the timeline for response, where the dotted line represents median time to response (1.9 months). One patient with progression based on a new site of disease did not have post-baseline assessment of RECIST target lesions and so is not shown in **a**.

Overall response rate (ORR) was the primary endpoint of phase 2 of this study. In the 23 patients eligible for efficacy analyses, the ORR was 57% (95% confidence interval (CI), 35–77); three (13%) had a confirmed complete response (CR); and ten (43%) had a confirmed partial response (PR) (Table 2). Target tumor shrinkage per Response Evaluation Criteria in Solid Tumors (RECIST) version 1.1 was seen in 91% of patients with post-baseline tumor assessments (Fig. 2a); one patient with progression based on a new site of disease did not have post-baseline assessment of RECIST target lesions.

**Table 2:** Summary of tumor response

Response, *n* (%)	*RET* fusion–positive solid tumors (*n* = 23)^a^
ORR^b^ (95% CI)	13 (57) (35–77)
CR	3 (13)
PR	10 (43)
SD	6 (26)
PD	4 (17)
CBR^c^ (95% CI)	16 (70) (47–87)
DCR^d^ (95% CI)	19 (83) (61–95)
Median DOR, months (95% CI)^e^	11.7 (5.5–19.0)

Response rates and the number of patients with each individual response per RECIST version 1.1 in the efficacy-evaluable population (*n* = 23). Two-sided 95% CIs were based on exact binomial distributions using the Clopper–Pearson method. ^a^Enrollment as of 18 February 2021 and data cutoff date 18 October 2021. Excludes three patients (two with colon cancer and one with cholangiocarcinoma) with additional driver mutations (*KRAS*, *PIK3CB* and *BRAF*). ^b^Confirmed CR or PR. ^c^Confirmed CR, PR or SD ≥16 weeks. ^d^Confirmed CR, PR or SD. ^e^Kaplan–Meier estimated.

Confirmed tumor responses were observed in all four patients with pancreatic cancer (including one CR), two of three patients with cholangiocarcinoma, two of three patients with sarcoma (including one CR), two of three patients with neuroendocrine cancer and single patients with head and neck cancer and unknown primary tumor (CR). The other patient with cholangiocarcinoma had a single timepoint response before discontinuing treatment due to an AE.

**Extended Data Fig. 4:**
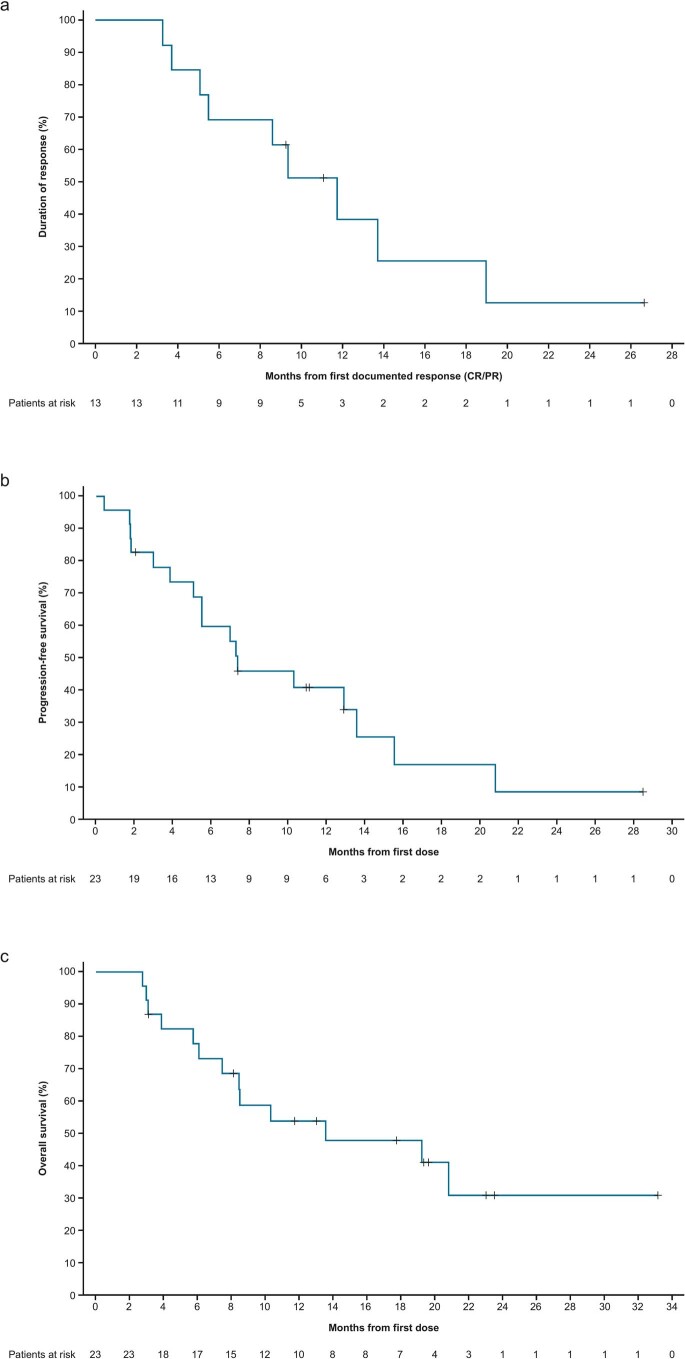
**a**, Duration of response in 13 patients with *RET* fusion–positive tumors in the efficacy-evaluable population with a confirmed CR or PR. **b**, Progression-free survival in 23 patients with *RET* fusion–positive tumors in the efficacy-evaluable population. **c**, Overall survival in 23 patients with *RET* fusion–positive tumors in the efficacy-evaluable population.

The clinical benefit rate (CBR) was 70% (16/23 patients), and the disease control rate (DCR) was 83% (19/23 patients) (Table 2). Median duration of response (DOR) was 11.7 months (95% CI, 5.5–19.0) with a median follow-up of 26.7 months (95% CI, 9.3–26.7) (Extended Data Fig. 4a). DOR rates were 69% (95% CI, 44–94) at 6 months and 39% (95% CI, 8–69) at 12 months. Of the 13 patients with CR or PR, DOR was ≥6 months for nine (69%) (Fig. 2b); two (15%) had response durations of ≥18 months, one (8%) of whom had a response duration of ≥24 months. Median time to response was 1.9 months (range, 1.7–3.6); at data cutoff, 31% (4/13) of patients had ongoing responses.

Although individual results may vary, among the patients with tumor response, three cases were particularly notable. A man in his early 30s had pancreatic cancer (2.5-cm pancreatic head mass) with multiple hepatic metastases (largest 2.3 cm and 2.4 cm) and multiple peripancreatic lymph nodes at treatment initiation, and his tumor harbored *RET–TRIM33* and *RET–JMJD1C* fusions; other genomic alterations detected on liver biopsy were FGFR4 p.R493Q, which was a variant of unknown significance, and PTCH1 p.1287_1303del and *PTEN* copy number loss, which were not considered actionable drivers. Baseline cancer antigen (CA) 19-9 was 12.6 U ml^−1^, and baseline carcinoembryonic antigen was 2.5 ng ml^−1^. This patient, who had previously experienced progressive disease (PD) and treatment-limiting toxicity on one prior line of chemotherapy (PD and toxicity on capecitabine), had a CR with pralsetinib (100% decrease in the sum of lesion diameters (SLD)). This patient continued treatment with an ongoing CR at a treatment duration of 33.1 months as of the 18 October 2021 data cutoff.

**Fig. 3:**
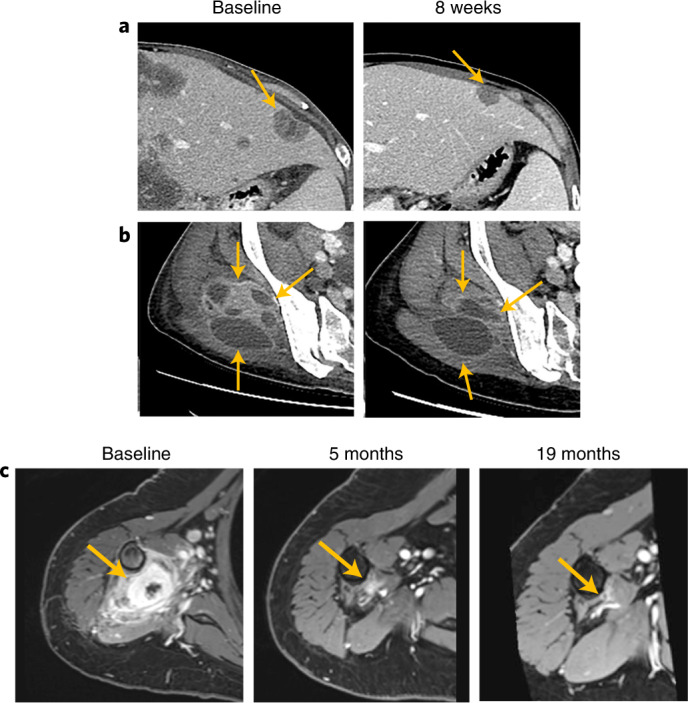
Time-dependent disease evaluations in two patients after pralsetinib treatment. Baseline and 8-week disease evaluation in a 51-year-old woman with *RET–NCOA4* fusion–positive cholangiocarcinoma: **a**, at first disease evaluation after 8 weeks receiving pralsetinib, a left hepatic lobe lesion measuring 2 × 3 cm at baseline had reduced to 1.2 × 1.9 cm; **b**, a prior heterogeneously enhancing soft tissue mass in the right gluteal muscles had decreased in size and enhancement and showed increased cystic and necrotic components compared to baseline scans. **c**, Baseline, 5-month and 19-month disease evaluation in a 60-year-old woman with a *RET–CCDC6* fusion–positive sarcoma presenting as two muscular masses in the right upper arm.

A woman in her early 50s had an intrahepatic cholangiocarcinoma with a *RET–NCOA4* fusion and metastases to liver and bone on diagnosis. Diagnostic imaging revealed a large mass with at least a 13 × 8 cm diameter and 20 satellites of different diameters (most ~15 mm) (Fig. 3a,b). The patient had a PR with pralsetinib after experiencing a best response of PD on all three prior lines of therapy (received for ≤3 months: gemcitabine/cisplatin/abraxane, erlotinib/bevacizumab and osimertinib). Other genomic alterations identified in this patient were *EGFR* A1118T, which was not an actionable driver, and *CDKN2A/B* loss, which was a variant of unknown significance; microsatellite status was stable, and tumor mutation burden was low (three mutations per megabase). Liver immunohistochemistry was positive for CK7 and CDX-2 and negative for CK20. Throughout treatment with pralsetinib, CA 19-9 reduced from 1 × 10^6^ U ml^−1^ to 82 U ml^−1^, and CA-125 reduced from 1,591 U ml^−1^ to 16.4 U ml^−1^, with rapid and near-complete clearance of *RET–NCOA4* fusion ctDNA. This patient received treatment with continued tumor shrinkage (to a maximum of 77% reduction in measurable disease) for 20.7 months before ultimately succumbing to PD, leading to treatment discontinuation and death before the data cutoff.

A woman in her early 60s with a sarcoma (malignant mesenchymal tumor) with a *RET–CCDC6* fusion and no metastases had a PR after 1.9 months of pralsetinib treatment that had evolved to a CR at the time of the data cutoff (100% decrease in SLD), with treatment duration of 19.4 months (Fig. 3c). She had two muscular masses on her right upper arm, and the initial pathologic diagnosis after resection was pigmented villonodular synovitis (PVNS). Given this diagnosis, the patient received imatinib as first-line therapy but progressed, with locoregional recurrence with at least four macroscopic tumor nodules in the right arm that were considered inoperable. In parallel to RNA sequencing analysis that confirmed a *RET–CCDC6* fusion, transcriptomic analysis suggested that the diagnosis was not PVNS but, rather, an undifferentiated histiocytic tumor. Other mutations in the whole-exome RNA sequence included an in-frame fusion of type FN1-PRG4 as well as the reciprocal transcript (also in-frame) and mutations in exon 43 of *ATM* (p.R2105G) and exon 5 of *PDGFRB* (p.R251H). These three mutations were variants of unknown significance. At the time of treatment initiation, a total of seven nodules (8–50 mm) were found on pre-treatment magnetic resonance imaging (MRI) in the soft tissue of the right arm as well as two right axillary lymph nodes (10 mm and 12 mm). As of October 2021, the CR was ongoing after 19.6 months of follow-up.

Among all 23 patients, median progression-free survival (PFS) was 7.4 months (95% CI, 5.1–13.6) at a median follow-up of 28.5 months (95% CI, 10.9–28.5) (Extended Data Fig. 4b), with a PFS rate of 60% (95% CI, 39–80) at 6 months and 41% (95% CI, 20–62) at 12 months. Median overall survival (OS) was 13.6 months (95% CI, 7.5–not reached) with median follow-up of 23.5 months (95% CI, 19.8–23.9) (Extended Data Fig. 4c), with an OS rate of 78% (95% CI, 61–95) at 6 months and 54% (95% CI, 33–75) at 12 months.

### Safety

**Extended Data Fig. 5:**
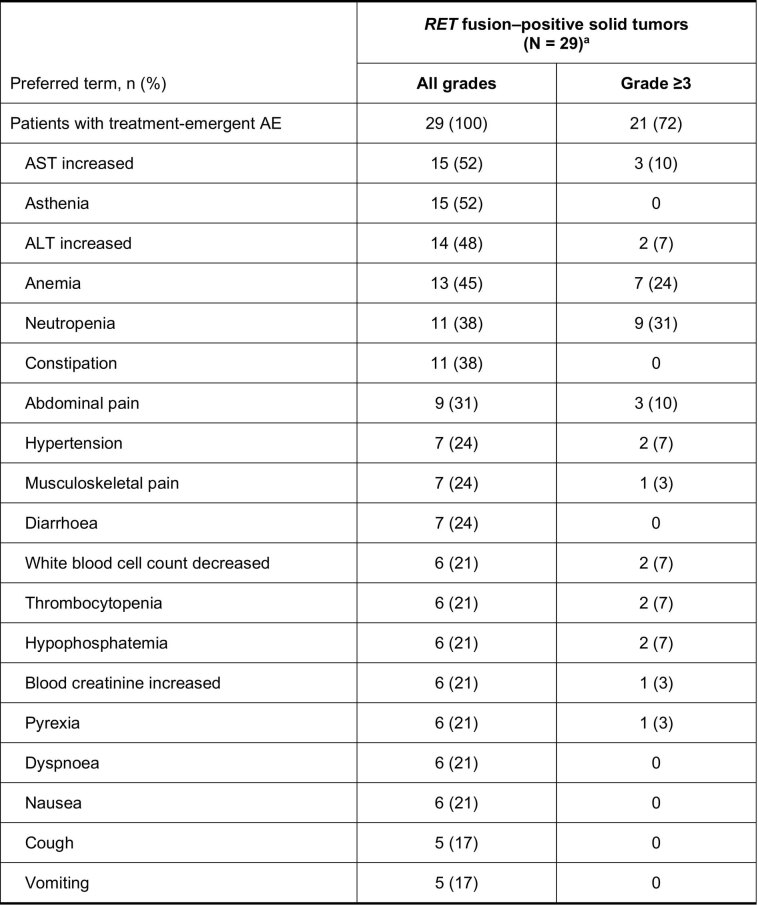
Treatment-emergent adverse events occurring in ≥15% of patients. ^a^Enrollment and data cutoff date as of 18 October 2021.

For the 29 patients in the safety population, median relative dose intensity was 86% (range, 51–124), with a median daily dose of 397 mg (range, 212–400), and median time on treatment was 7.0 months (range, 0.4–33.1). Dose intensity was calculated based on starting dose; as the patient who initiated at 200 mg/100 mg twice daily dosing subsequently received 400 mg QD, this patient had a dose intensity >100%. All patients experienced treatment-emergent AEs, of whom 21 (72%) experienced grade ≥3 events (Extended Data Fig. 5). Treatment-related adverse events (TRAEs) occurred in 25 patients (86%), of whom 20 (69%) experienced a grade ≥3 TRAE (Table 3). The most common any-grade TRAEs were increased aspartate transaminase (AST; 11 patients (38%)), increased alanine transaminase (ALT; ten patients (34%)) and neutropenia (ten patients (34%)). Grade 4 events were experienced by two patients (7%); one patient experienced thrombocytopenia, and one patient experienced thrombocytopenia, pancytopenia and acute kidney injury. One death occurred in which the cause was unknown; this patient had multiple possible causes of death, including disease progression, pulmonary infection, respiratory failure, cardiac insufficiency and hypertensive heart disease. The death was recorded as treatment related because the cause could not be unequivocally excluded.

**Table 3:** Treatment-related adverse events (TRAEs)

Preferred term, *n* (%)	*RET* fusion–positive solid tumors (*n* = 29)^a^	
	All grades	Grade ≥3
Patients with TRAEs	25 (86)	20 (69)
Increased AST	11 (38)	3 (10)
Increased ALT	10 (34)	2 (7)
Neutropenia	10 (34)	9 (31)
Anemia	9 (31)	4 (14)
Constipation	7 (24)	0
Decreased white blood cell count	6 (21)	2 (7)
Thrombocytopenia	5 (17)	2 (7)
Hypertension	5 (17)	2 (7)
Asthenia	5 (17)	0

TRAEs that occurred in ≥15% of patients in the safety population (*n* = 29), which were graded according to the Common Terminology Criteria for Adverse Events version 4.03, with terms pooled. ^a^Enrollment and data cutoff date as of 18 October 2021.

In total, 17 patients (59%) had transient dose interruptions due to TRAEs, and 13 patients (45%) had permanent dose reductions due to TRAEs. The most common TRAEs leading to dose interruption were neutropenia (eight patients (28%)), anemia and increased AST (each three patients (10%)) and thrombocytopenia and increased ALT (each two patients (7%)). The most common TRAEs leading to dose reduction were neutropenia (eight patients (28%)), anemia, increased AST and increased ALT (each two patients (7%)).

## Discussion

In this phase 1/2 study of pralsetinib in patients with advanced or metastatic *RET* fusion–positive solid tumors, almost all of whom were previously treated with systemic therapy, pralsetinib showed robust and durable anti-tumor activity regardless of tumor type or *RET* fusion partner. *RET* fusions have been identified as oncogenic drivers in multiple tumor types^4–8,25^, and, generally, standard therapies that are effective in tumors without oncogenic drivers are less effective than targeted therapies^26–28^. Precision oncology paradigms that comprise identification of oncogenic alterations through clinical NGS and subsequent application of genomically targeted therapies are applicable to multiple malignancies. Herein, *RET* fusions defined a unique subset of alterations across multiple tumor types (>15 including NSCLC and multiple subtypes of thyroid cancer) targeted by pralsetinib, validating RET as a tissue-agnostic target.

In this patient group, whose disease was resistant to prior treatments where available, treatment with pralsetinib resulted in an ORR of 57% across seven tumor types, and clinical benefit was reported in 70% of patients by blinded independent central review (BICR). This compares to an ORR of 61% and 70% in patients with *RET* fusion–positive NSCLC who received prior platinum therapy and no prior systemic treatment, respectively, and an ORR of 89% for patients with *RET* fusion–positive thyroid cancer in previously published data on the ARROW study^10,11^. Despite the small number of patients, responses were seen in all four patients with pancreatic cancer (including an ongoing CR with treatment duration of 33.1 months) as well as in two of the three patients with cholangiocarcinoma (including a patient who received treatment for over 20 months after a best response of PD on all three prior lines of therapy). These are encouraging findings because these tumor types are difficult to treat, and the unmet need for better treatments to improve clinical benefit is high. Indeed, response rates for standard-of-care therapies are 26% in biliary cancers (cisplatin plus gemcitabine^29^) and 23–32% (first-line oxaliplatin-based combination chemotherapy and first-line gemcitabine plus nab-paclitaxel) in pancreatic cancer^30,31^. In ARROW, responses were seen in treatment-naive patients who were not candidates for standard therapies and in patients who had received several prior lines of therapy, highlighting the need for targeted therapies across a range of tumor types for patients who currently have no standard of care and for those who have exhausted all other options. The strategy of treating patients with *RET* fusion–positive solid tumors with targeted therapies is also supported by results with selpercatinib: in an analysis that included adult patients with locally advanced or metastatic *RET* fusion–positive non-lung/non-thyroid solid tumors who received selpercatinib twice daily, the ORR was 47%^32^. The efficacy-evaluable population comprised patients who were enrolled long enough to allow a 6-month follow-up from their first dose.

The safety profile reported in this analysis is consistent with previously reported results in patients with *RET* fusion–positive NSCLC and thyroid cancer from the ARROW study^10,11^, with no new safety signals identified, and no effect of pralsetinib on QT interval was observed^11^. The most common TRAEs were increased ALT/AST and neutropenia. Common TRAEs seen with selpercatinib include increased ALT/AST, dry mouth, diarrhea and fatigue^1,32^. For patients with other solid tumors who received selpercatinib in the LIBRETTO-001 study, a grade ≥3 TRAE of QT interval prolongation was reported in 4% of patients^32^.

ARROW is a single-arm study with no comparator group. The safety population for the cohort analyzed here included a small heterogeneous number of patients (*n* = 29); despite this, all but two patients included in the efficacy-evaluable population (*n* = 23), a subset of the safety population, experienced a tumor shrinkage. In combination with the robust activity seen in patients with NSCLC and thyroid cancer in the ARROW study^10,11^, these data further support the potential of pralsetinib to address the unmet medical need across a broad range of *RET*-altered tumor types with differing histology.

Overall, these data highlight the need for broad *RET* testing, preferably by NGS, to identify candidates who may benefit from treatment with pralsetinib. Enrollment of patients with other *RET* fusion–positive solid tumors in ARROW is ongoing.

## Methods

### Study design and patient population

ARROW (NCT03037385) is an open-label, international, phase 1/2 study evaluating the efficacy and safety of pralsetinib across various *RET*-altered solid tumors conducted at 84 sites across 13 countries. The phase 1 dose-escalation portion identified the maximum tolerated dose and recommended phase 2 dose of pralsetinib as 400 mg QD^10^. Adults with unresectable, locally advanced or metastatic solid tumors were enrolled into nine phase 2 groups as defined by disease type and prior therapy status. This current analysis reports results for the subgroup of patients with *RET* fusion*–*positive solid tumor types, excluding NSCLC and thyroid cancer, who were enrolled in the phase 1 study portion and in the phase 2 expansion group 5. In accordance with study eligibility requirements, these patients had previously received or were not candidates for appropriate standard-of-care therapy. Additional eligibility criteria were as previously reported^10^.

This study was conducted in accordance with the ethical principles of Good Clinical Practice and the Declaration of Helsinki, and was based on the International Council for Harmonisation E6 requirements. The full protocol was approved by the institutional review board or independent ethics committee of each participating site, and all patients provided signed informed consent. The name of each participating institute, organization or site whose ethical committee approved the protocol is provided in the Supplementary Information.

### Outcomes

Phase 2 primary endpoints were ORR (defined as the proportion of patients who had confirmed CR or PR per RECIST version 1.1) and safety. Key secondary endpoints included CBR (defined as the proportion of patients who had confirmed CR, PR or SD lasting *≥*16 weeks); DCR (defined as the proportion of patients who had confirmed CR, PR or SD); DOR (defined as time from first documented tumor response (CR/PR) until first documented disease progression or death); PFS (defined as time from first dose of pralsetinib to first documented disease progression or death due to any cause); and OS (defined as time from first dose of pralsetinib to death due to any cause).

### Assessments

Tumor response per RECIST version 1.1 was assessed by BICR. Computed tomography or MRI of all known disease sites was performed at screening and approximately every 8 weeks during treatment. For the purpose of study eligibility, *RET* fusions were identified by local testing using NGS, FISH or GeneTrails Solid Tumor Fusion Panel, which used DNA and RNA, with RNA used to identify *RET* fusions. In accordance with the statistical analysis plan, patients were confirmed as *RET* fusion positive if any one of these methods returned a positive fusion result. The presence of concurrent non-*RET* fusion oncogenic drivers was determined prospectively based on local testing and/or by retrospective central analysis if necessary. As per the study protocol, concurrent drivers were defined as known primary driver alterations consistent with the scientific literature for different tumor types, and the final decision was made by the sponsor. AEs were graded according to the Common Terminology Criteria for Adverse Events version 4.03, and terms were pooled.

### Statistical analysis

All patients with *RET* fusion–positive solid tumors, excluding NSCLC and thyroid cancer, who were enrolled by the analysis cutoff date (18 October 2021) were included in the safety analyses. Of these patients, those who began treatment by the enrollment cutoff date (18 February 2021), who had baseline measurable disease per BICR, who had at least one evaluable post-baseline disease response assessment and who were without other known oncogenic mutations were included in efficacy analyses. The enrollment cutoff for efficacy analyses was employed to provide adequate follow-up time for responses to pralsetinib. Two-sided 95% CIs were based on exact binomial distributions using the Clopper–Pearson method. DOR, PFS and OS were analyzed using the Kaplan–Meier method. Estimates of follow-up duration for DOR, PFS and OS were based on the inverse Kaplan–Meier method, with 95% CIs based on the Greenwood formula.

For group 5, which excluded patients with *RET* fusion–positive NSCLC but included patients with *RET* fusion–positive thyroid cancer, a total sample size of 100 patients with solid tumors harboring a *RET* fusion was intended to allow >90% power at the two-sided significance level of 0.05 for testing the assumption of null hypothesis of ORR = 0.1 versus the alternative ORR = 0.3. As results for 20 patients with *RET* fusion–positive thyroid cancer were reported previously^11^, patients with these cancers were excluded from this interim analysis. All statistical analyses were performed with SAS version 9.4 software.

### Reporting summary

Further information on research design is available in the Nature Research Reporting Summary linked to this article.

## Online content

Any methods, additional references, Nature Research reporting summaries, source data, extended data, supplementary information, acknowledgements, peer review information; details of author contributions and competing interests; and statements of data and code availability are available at 10.1038/s41591-022-01931-y.

## Supplementary information

Supplementary Information Protocol and list of study sites whose ethics committees approved the protocol

Reporting Summary

## Data Availability

The anonymized derived data from the registrational ARROW study (NCT03037385) that underlie the results reported in this article may be made available after Roche and/or Blueprint Medicines have received regulatory approval for pralsetinib in the United States and the European Union in the tumor-agnostic setting described herein or upon terminating its clinical development in this setting. Qualified researchers can then request access to individual patient-level clinical data through a data request platform. At the time of writing, this platform is Vivli (https://vivli.org/ourmember/roche/). As *RET* fusions are rare alterations, the anonymization of patient-level data in patient subgroups or trial cohorts of fewer than 50 patients may be difficult to achieve. As a result, Roche will assess the feasibility of anonymization and, therefore, data release as part of the review of inquiries. For up-to-date details on Roche’s Global Policy on the Sharing of Clinical Information and how to request access to related clinical study documents, see https://go.roche.com/data_sharing.
