# Supplementary material for: Pan-cancer efficacy of pralsetinib in patients with RET fusion–positive solid tumors from the phase 1/2 ARROW trial
Source: Nat Med. 2022 Aug 12;28(8):1640–5. doi: 10.1038/s41591-022-01931-y (PMC9388374; doi:10.1038/s41591-022-01931-y)
Supplement: Supplementary file 1 — Protocol and list of study sites whose ethics committees approved the protocol [file 41591_2022_1931_MOESM1_ESM.pdf]

nature portfolio
https://doi.org/10.1038/s41591-022-01931-y

Supplementary information

Pan-cancer efficacy of pralsetinib in patients with *RET* fusion–positive solid tumors from the phase 1/2 ARROW trial

In the format provided by the authors and unedited

Subbiah V et al.

Pan-cancer efficacy of pralsetinib in patients with *RET* fusion–positive solid tumors from the phase 1/2 ARROW trial

Supplementary Information

Table of Contents

Study Protocol ………………………………………………………………………........................ 1

Supplementary Table 1 | Study Sites Whose IEC/IRB Approved the Protocol …….........140

CLINICAL RESEARCH PROTOCOL

DRUG:	Pralsetinib (previously known as BLU-667)
STUDY NUMBER:	BLU-667-1101
PROTOCOL TITLE:	A Phase 1/2 Study of the Highly-selective RET Inhibitor, BLU-667, in Patients with Thyroid Cancer, Non-Small Cell Lung Cancer (NSCLC) and Other Advanced Solid Tumors
IND NUMBER:	131825
EudraCT NUMBER	2016-004390-41
SPONSOR:	Blueprint Medicines
ORIGINAL PROTOCOL DATE:	08 November 2016
VERSION NUMBER:	Amendment 13
VERSION DATE:	08 July 2020

CONFIDENTIALITY STATEMENT
The information contained herein is confidential and the proprietary property of Blueprint Medicines and any unauthorized use or disclosure of such information without the prior written authorization of Blueprint Medicines is expressly prohibited.

CLINICAL PROTOCOL

**Protocol Title:**	**A Phase 1/2 Study of the Highly-selective RET Inhibitor, BLU-667, in Patients with Thyroid Cancer, Non-Small Cell Lung Cancer (NSCLC) and Other Advanced Solid Tumors**
**Study No:**	**BLU-667-1101**
**Original Protocol Date:**	**08 November 2016**
**Protocol Version No:**	**Amendment 13**
**Protocol Version Date:**	**08 July 2020**

This study protocol was subject to critical review and has been approved by the Sponsor. The information contained in this protocol is consistent with the current risk-benefit evaluation of the investigational product.

The Investigator will be supplied with details of any significant or new findings, including adverse events, relating to treatment with the investigational product.

[redacted]

AMENDMENT 13 RATIONALE

This amendment updates and supersedes Global Protocol BLU-667-1101 Amendment 9 dated 03 July 2019

Major changes include the following:

- Group 5: To more accurately characterize the activity of pralsetinib across various RET fusion-positive solid tumor types, the sample size of Group 5 has increased from N ~ 40 to N ~ 100 patients. (increasing the study's total sample size to 647 patients). As the intent of this cohort is to enroll a variety of RET-fusion tumor types, it is also clarified that Blueprint will notify sites if/when sufficient data are available and accrual should cease for a particular tumor type. Additionally, to ensure enrollment of a population with unmet medical need, patients should have previously received SOC appropriate for their tumor type, unless there is no accepted standard therapy for the tumor type or the Investigator has determined that treatment with standard therapy is not appropriate. Finally, given the primary endpoint of ORR, it is now required that subjects in Group 5 must have at least 1 target lesion evaluable by RECIST v1.1.

- Phase 2 secondary objective in NSCLC patients and exploratory objective in patients with tumor types other than NSCLC have been updated to assess brain metastases activity in patients with measurable (target by RECIST v1.1) lesions in the brain by blinded independent central review (brain metastases sub-population) and assess the time to intracranial progression in all patients.

- An additional analysis population, the RET-altered Measurable Disease Population, has been added to provide an assessment of the activity of pralsetinib in a mechanistically-relevant population. New language clarifies that this will be the primary analysis population for ORR, DOR, CBR and DCR, while the remaining efficacy endpoints will be examined using the Efficacy Population.

- Subgroup analyses will be completed for Groups 2 and 4, consisting of:
  1) patients enrolled prior to Protocol Amendment 9 (who were not candidates for standard therapies and thus may have had a poor prognosis) and 2) those enrolled on or after Protocol Amendment 9 (possibly a more typical first-line population). This will allow examination of the activity of pralsetinib in more typical first-line populations of patients with NSCLC and MTC.

- Pharmacodynamic parameters of pralsetinib will include changes in tumor/blood including, but not limited to, changes in blood calcitonin and CEA and biochemical response rate (MTC patients only).

- Group 2: The primary intent of this cohort is to assess the activity of pralsetinib in the treatment-naïve patients with NSCLC; therefore, text was added to clarify that this group will include no more than 30 patients with prior systemic therapy.

- Updated study visit frequency after Cycle 17. All study visits after Cycle 17 will occur at every 4 cycles (16 weeks) (e.g. Cycle 21, Cycle 25…_); study visit frequency and imaging was aligned within the Schedule of Assessments table.

- Adjusted pralsetinib dose modification recommendations for treatment-related Grade 1 and Grade 2 pneumonitis.

Minor changes include the following:

- Name of the investigational product updated from BLU-667 to pralsetinib.

- Updated background information for consistency with IB version 4.0 updates along with updated efficacy, PK, and safety data from interim analyses.

- Deleted text to clarify the role of IDMC; IDMC review of study progress does not include primary efficacy outcomes.

- Updated the Schedule of Assessments footnote "s" and the Schedule for PK Sample Collection footnote "g" to specify that Cycle1 Days 2 and 16 will only be for ~12 patients in Groups 8 and 9 enrolled under Protocol Amendment 10. The overall number of patients for serial PK changed from 60 to 72.

- Incorporated major revisions to China-specific amendments (10 and 12). The updates to protocol amendment 13 include the addition of Groups 8 and 9, updates to the serum chemistry test panel to assess either bicarbonate (venous) or CO2 level in blood, and updates to the urinalysis test panel updated to assess either leukocyte esterase or white blood cells. These changes are applicable only for patients in China.

AMENDMENT 12 RATIONALE

Protocol Amendment 12 was made for all investigative sites in China. Protocol Amendment 12 is not active outside of China. This amendment updates and supersedes Protocol BLU-667-1101 Amendment 10 dated 26 July 2019.

- Allowed inclusion of patients in Group 9 who received prior radiotherapy, surgery, or systemic cytotoxic chemotherapy.
- Updated contact information for reporting of serious adverse events
- Allowed white blood cells to be evaluated instead of leukocyte esterase in the urinalysis (dipstick) test for sites in China.
- Corrected formatting, grammatical, and typos.

AMENDMENT 11 RATIONALE

This amendment updates and supersedes Global Protocol BLU-667-1101 Amendment 9 dated 3 July 2019 for all investigative sites in France. This protocol amendment is not active outside of France. The following changes were made to address feedback from the Agence Nationale de Sécurité du Médicament et des Produits de Santé (ANSM).

- Clarified typographical errors in section 6.4.2 (concomitant therapy to be used with caution) introduced in Protocol Amendment 9 (3 July 2019)

- Clarified that Group 4 (medullary thyroid cancer patients that do not have a RET mutation) will not be enrolled in the BLU-667-1101 clinical trial in France

Details of all changes are presented in the "Protocol Amendment – Summary of Changes" document dated 23 September 2019.

AMENDMENT 10 RATIONALE

Amendment 10 was made for all investigative sites in China. Protocol Amendment 10 is not active outside of China. This amendment updates and supersedes Protocol BLU-667-1101 Amendment 8 dated 20 December 2018. The changes were made in reference to Global Protocol BLU-667-1101 Amendment 9.

AMENDMENT 9 RATIONALE

This amendment updates and supersedes Global Protocol BLU-667-1101 Amendment 7 dated 12 December 2018.

Major Changes include the following:

- The study phase was updated from a Phase 1 to a Phase 1/2 to better describe the study design with the increased patient population and hypothesis testing.
  - Throughout the protocol, Part 1 and Part 2 have been replaced with Phase 1 and Phase 2, respectively.
- Due to the encouraging initial data in treatment naïve RET fusion-positive NSCLC patients, the Phase 2, Group 2 sample size was increased to 200 patients to allow for enrollment of treatment naïve RET fusion-positive NSCLC patients (increasing the study's total sample size to 527 patients).
  - The enrollment for this group has been increased to provide >90% power to test the efficacy hypothesis as described in Section 11.3.2 in treatment naïve RET fusion positive NSCLC patients.
  - Inclusion criteria 3 and 4 were edited to allow for treatment naïve patients in Group 2.
- Exclusion criteria 7 was edited to clarify that patients who have received previous immunotherapy or other antibody therapy cannot be started on BLU-667 within the defined washout of 28 days.
- A Phase 2 independent data safety monitoring committee (Section 12.2.2) is replacing the Phase 2 Safety Review Committee (Section 12.2.1) to account for the increased number of patients in the study.
- To help alleviate patient burden, Cycle 1 Days 2 and 16 were removed as serial PKs are no longer required.
- Stopping rules were updated to account for increased sample size and move out of the body of the protocol to Appendix 7.
- Dose modification for Grade 3 or worse pneumonitis was modified in Table 8 for additional safety measures that patients should be permanently discontinued from study treatment, unless the Investigator discusses with the Sponsor and believes the risk-benefit justifies retreatment.
- The statistics section (Section 11) was updated to align with the statistical analysis plan for the study. Following changes were made:
  - Provided justification for increased sample size

- Aligned analysis population definitions with study objectives/endpoints
- Swimmer plots to display tumor burden over time and Spider plots to display tumor reduction will not be produced
- Clarified that Patients enrolled in Phase 1 and treated at the RP2D will be pooled together with the appropriate Part 2 group patients for analyses.
- Predefined 95% Confidence Intervals of ORR were updated based on increased sample size

Other minor updates include the following:

- Clarified in Section 5.5 that screen failure patients need to be entered into the clinical database.
- RET pre-screening in Section 7.1 was updated to remove the central laboratory for RET pre-screening as all investigational sites will use local methods.
- Details regarding OS follow-up were included in Sections 5.6.2, 6.6, 7.9.

AMENDMENT 8 RATIONALE

Amendment 8 was made for all investigative sites in China. Protocol Amendment 8 is not active outside of China.

AMENDMENT 7 RATIONALE

This amendment updates and supersedes Global Protocol BLU-667-1101 Amendment 4.1 dated 25 July 2018, Republic of Korea Protocol Amendment 5 dated 25 July 2018, and France Protocol Amendment 6 dated 27 August 2018.

Major changes include the following:

- The Part 2, dose expansion group definitions were adjusted to better account for current approved standard of care agents.
  - The study schema showing the adjustments to the Part 2 dose expansion groupings is found in the study schematic in the protocol synopsis and in the Study Plan (Section 4). Briefly, the non-small cell lung cancer (NSCLC) groups (Groups 1 and 2) were updated to reflect previous treatment with a regimen that contained platinum chemotherapy versus a multi-kinase inhibitor (MKI). The medullary thyroid cancer (MTC) groups (Groups 3 and 4) were updated to reflect previous treatment with one of the approved MKIs (cabozatinib and/or vandetanib) and not any MKI. Finally, the previous other *RET*-altered solid tumor group (former Group 5) was split into 2 separate groups: rearranged during transfection (RET) fusions (new Group 5) or RET mutations (new Group 7). This was done in recognition that solid tumor patients with *RET*-fusions and *RET*-mutations may be distinct clinical and/or regulatory populations.
  - The overall enrollment for Part 2 (Dose Expansion) has been increased to provide >95% power in Groups 1 and 3, >90% power in Group 5, and ~68% power in Group 2 to test the efficacy hypothesis for each group as described in Section 11.3.2. The goal of each of the Part 2 groups remains to evaluate the safety, efficacy, and pharmacokinetics (PK) of BLU-667 in *RET*-altered patients treated at the maximum tolerated dose (MTD)/recommended Phase 2 dose (RP2D) in various patient populations. Specifically, the study rationale and design have been updated (Section 1.3); the study's objectives (Section 2) and endpoints (Section 3) have been edited and split into Part 1 and Part 2 sub-sections for clarity and additional text has been added to justify the number of patients needed to test the efficacy hypothesis and the detection of adverse events (Section 11.3.2).
- For additional safety precautions the following exclusion criteria were added/edited: added exclusion criterion 2g for patients with Grade 2 or higher serum phosphorous at baseline; added an exclusion criterion 6 to exclude patients with the presence of clinically significant interstitial lung disease or interstitial pneumonitis; and clarified the washout period for immunotherapy or other antibody therapy should be 28 days in exclusion criterion 7.
- Divided the dose and administration section (Section 6.3) into subsections that apply to Part 1 (Dose Escalation) and those that apply to Part 2 (Expansion) to provide

additional detail and guidance on adverse event management and dose adjustments in Part 2 of the study.

- o In the dosing for Part 2 (Section 6.3.2) section edited and clarified the instructions for dosing and dose interruptions (Section 6.3.2.2), added dose modification tables for hematologic and non-hematologic adverse events (Table 7), and added dose modifications and management recommendations for specific treatment-related adverse events such as pneumonitis (Section 6.3.2.3.1), pneumonia/lung infections (Section 6.3.2.3.2), tumor lysis syndrome (Section 6.3.2.3.3), hyperphosphatemia (Section 6.3.2.3.4), and hypertension (Section 6.3.2.3.5).

- Made it clear that dose re-escalation after dose modification for adverse events is discouraged; however, clarified under what conditions it might be considered appropriate to re-escalate a patient's dose (no higher than the starting dose), after resolution of adverse events (Section 6.3.2.2)

- Created an additional Safety Review Committee (SRC) for Part 2 of the study and added a section to describe its membership and purpose (Section 12.2.2).

Minor changes include the following:

- Incorporated the updated Part 2 Groups re-organization (inclusion criterion 3); clarified that all patients must have been treated with prior standard therapy unless the Investigator has determined that treatment with standard therapy is not appropriate (inclusion criterion 4); clarified that groups 1, 2, 3, 4, and 6 need to have measurable disease by Response Evaluation Criteria in Solid Tumors (RECIST) v1.1 at baseline, but it is not required for Groups 5 and 7 (inclusion criterion 5).

- Updated Section 1.5 to include updated safety and efficacy data from the earlier part of the trial.

- Added sections to clarify RET Prescreening (Section 5.4 and Section 7.1)

- Updated the early stopping rule table (Appendix 8) to reflect the larger sample size

- Updated footnote "a" in the Schedule of Assessments (Section 6.6, Table 10) to indicate that certain labs and the physical exam required on C1D1 may be performed on D-1.

- Added a clarification that during screening and on study during physical exam assessments, MTC patients should be asked and have recorded information on bowel movements (Section 7.2 and 7.3.1).

- Added that immediate release tablets of BLU-667 may be used during the trial, if and when they become available (Section 8.1.1 and 8.1.2) and updated packaging information (Section 8.2).

- Clarified the definition of Response-Evaluable (RE) population (Section 11.2)
- Moved medications that are sensitive CYP2C9 substrates with Narrow Therapeutic Index's such as Warfarin from the list of prohibited medications (Section 19.2 Appendix 2) to the list of Medications to be used with Caution (Section 19.3 Appendix 3).
- Corrected formatting, grammatical, and typo corrections.

Details of the changes are presented in the "Protocol Amendment – Summary of changes" document dated 12 December 2018

AMENDMENT 6 RATIONALE

Amendment 6 was made for all investigative sites in France. Protocol Amendment 6 is not active outside of France.

AMENDMENT 5 RATIONALE

Amendment 5 was made for all investigative sites in the Republic of Korea. Protocol Amendment 5 is not active outside of the Republic of Korea.

AMENDMENT 4.1 RATIONALE

This amendment updates and supersedes Global Protocol BLU-667-1101 Amendment 2 dated 02 February 2018 and Republic of Korea Protocol Amendment 3 dated 08 February 2018.

The amended protocol includes 2 major changes as follows:

- First, based on the favorable tolerability and encouraging efficacy observed in Part 1 (dose escalation), the sample size of Part 2 (dose expansion) has been increased.
    - A summary of Part 1 including determination of the maximum tolerated dose (MTD), overall safety, and preliminary efficacy for the dose escalation has been added - see Section 1.5.
    - The overall enrollment for Part 2 (dose expansion) has been increased to further evaluate the safety, efficacy, and pharmacokinetics (PK) of BLU-667 in rearranged during transfection (RET)-altered patients treated at the MTD/recommended Phase 2 dose (RP2D). Specifically, the study rationale and design has been updated (Section 1.3); the stopping rules for potential drug-related toxicity have been updated (Section 6.2.1); a primary efficacy objective/endpoint and corresponding statistics have been added for Part 2 to evaluate the overall response rate (ORR) in all RET-altered patients treated at the MTD/RP2D (Section 2.1, Section 3.1.1, and Section 11.4); additional text has

been added to justify the number of patients needed to test the efficacy hypothesis and detection of adverse events (Section 11.3.2).

- Based on the observed efficacy in dose escalation, exploratory objectives regarding assessment of additional measures of clinical benefit (eg, PFS, OS) and correlation of antitumor activity with RET gene status have been moved from exploratory objectives to secondary objectives.

- Second, an expansion group has been added to assess BLU-667 safety and efficacy in patients previously treated with a selective RET inhibitor (Group 6). This is reflected in the updated study design (Section 4.1.2) and entry criteria (Section 5.2). In addition, the medullary thyroid cancer (MTC) expansion group has been split into 2: the first (Group 3) including patients with MTC previously treated with a multikinase inhibitor (MKI) and the second (Group 4) including patients with MTC not previously treated with an MKI.

Minor changes include the following:

- Section 1.2 was updated to include biochemical activity of vascular endothelial growth factor receptor (VEGFR) inhibition observed biochemically in MKIs as compared to BLU-667.

- Section 1.3, Section 4.1.1, and Section 6 were revised to reflect the MTD/RP2D as well as the completion of Part 1.

- Since a clear efficacy signal was observed in the Part 1 dose escalation, the secondary objectives/endpoints were updated to include the assessment of additional measurements of clinical benefit (eg, duration of response [DOR], disease control rate [DCR], progression-free survival [PFS], and overall survival [OS]) and determination of potential correlations between measures of clinical benefit and RET gene status in plasma and tumor tissue (Section 2.1.2).

- Entry criteria (Section 5) were revised to clarify oncogenic RET alterations required for Part 2 enrollment. An appendix was added to list common oncogenic RET alterations (Appendix 6).

- Intra-patient dose escalation criteria were revised based on the observed reversibility of adverse events (AE) and overall favorable tolerability of BLU-667. Specifically, to achieve higher BLU-667 exposure, which may enhance the possibility of antitumor activity, Investigator may re-escalate to a higher dose level (≤ MTD) after AE resolution (Section 6.3.1.3).

- The schedule of assessments (Section 6.6) was updated to limit serial PK sampling and remove the on-treatment biopsy for Part 2 patients, to include the quality of life (QoL) questionnaire administration, and to add the mandatory pretreatment biopsy for Part 2, Group 6 patients.

- Section 7.1 was updated to reflect the changes to required screening assessments
- Section 7.5 was updated to reflect the changes made to biopsy requirements in Part 2.
- Section 7.6 was revised to clarify baseline screening scans and later timepoints.
- As some patients have reported subjective improvement in cancer-related symptoms (eg, diarrhea), Section 7.7 (patient-reported outcomes) was added to reflect the exploratory objective of QoL assessment measures for Part 2 patients to assess potential improvements in patient symptoms.

Details of the changes are presented in the "Protocol Amendment – Summary of changes" document dated 25 July 2018.

AMENDMENT 3 RATIONALE

Amendment 3 was made for all investigative sites in the Republic of Korea. Protocol Amendment 3 is not active outside of the Republic of Korea.

AMENDMENT 2 RATIONALE

This amendment updates and supersedes Protocol BLU-667-1101 Amendment 1.0 dated 09 January 2017, as follows:

- Section 1.4, Rationale for the Starting Dose and Dosing Regimen; Section 4.1.1, Part 1 (Dose-escalation); and Section 6.3, Dose and Administration were updated to clarify language regarding implementation of twice daily (BID) dose escalation to occur in parallel with QD dose escalation
- Language in the footnote of Table 11 describing electrocardiogram (ECG) monitoring was updated to clarify that all patients should have a single 12-lead ECG for adverse event (AE) monitoring, regardless of participation in Holter monitoring
- As BLU-667-1101 is a global trial, the following changes were made to address feedback from the French National Agency for Medicines and Health Products Safety (ANSM):
    - Exclusion criterion 1 was revised to exclude non-small cell lung cancer (NSCLC) patients with known BRAF mutations
    - Exclusion criterion 6 was revised to remove "which ever is shorter" regarding the washout requirements for prior anticancer therapy
    - Table 5 symbols were updated to reflect ≤ or ≥
    - Pregnancy testing was increased from bimonthly to monthly in order to detect any early pregnancy while on study. Updates were reflected in both Table 10: Schedule of Assessments and Section 7.3.5 Clinical Laboratory Tests

Details of the changes are presented in the "Protocol Amendment – Summary of changes" document dated 02 February 2018.

AMENDMENT 1 RATIONALE

This amendment updates and supersedes Protocol BLU-667-1101 Version 1.0 dated 08 November 2016, as follows:

- The Inclusion Criteria were updated to include only patients with non-resectable disease.
- Inclusion #6 was modified to make it clear that although the on-treatment tumor biopsy is strongly encouraged, it is not required, and the patient may decline the procedure.
- The Exclusion Criteria were updated to exclude patients with NSCLC with a targetable mutation in EGFR, ALK, or ROS1.
- In order to further protect the safety of patients, the study was updated to incorporate an enrollment stopping rule that terminates further enrollment to the study if there is an excess of permanent treatment discontinuations due to study drug-related AEs.
- An electrocardiogram measurement was added at Day 1 and Day 15, 4 hours postdose, to provide heart monitoring around the anticipated maximal plasma concentrations after the first dose and at steady state.
- As a precaution, the list of prohibited medications was updated to include strong inhibitors of CYP1A2, CYP2D6, and CYP3A4, as well as inducers of CYP3A4. Strong dual P-gp and CYP3A4 inhibitors are also excluded. Appendices 2 and 3 have been updated accordingly.

Typographical errors were corrected, and minor administrative changes made. Details of the changes are presented in the "Protocol Amendment – Summary of changes" document dated 09 January 2017.

STUDY SUMMARY

Title:	A Phase 1/2 Study of the Highly-selective RET Inhibitor, BLU-667, in Patients with Thyroid Cancer, Non-Small Cell Lung Cancer (NSCLC) and Other Advanced Solid Tumors
Study Centers:	The study will be conducted at multiple centers in the United States (US), Asia, and Europe.
Rationale:	RET is a receptor tyrosine kinase expressed in several neural, neuroendocrine and genitourinary tissues types that normally requires ligand and co-receptor binding for activation. Oncogenic activation of rearranged during transfection (RET) kinase activity has been observed in thyroid cancers where activating RET fusions (papillary thyroid cancer [PTC]) and RET mutations (medullary thyroid cancer [MTC]) occur. RET fusions have been found in tumors including non-small cell lung carcinomas (NSCLC), colon, breast, ovary, salivary carcinomas, and inflammatory myofibroblastic tumors, suggesting that RET fusions may be oncogenic in many tumor types.<br>RET activating mutations are observed in 50% to 80% of MTC patients and to a lesser extent in other cancers. RET fusions have been identified in approximately 1-2% of patients with NSCLC. Patients with NSCLC, thyroid cancer, or other tumors, and whose disease has relapsed after initial treatment with chemotherapy or multikinase inhibitors (MKIs) have limited treatment options, and National Clinical Practice Guidelines in Oncology (NCCN) guidelines recommend evaluation for clinical trials.<br>Pralsetinib (previously known as BLU-667) is a potent and selective inhibitor of RET and oncogenic RET mutants. In vivo, dose dependent antitumor efficacy with pralsetinib was demonstrated in several RET-driven tumor models. Antitumor efficacy was correlated with pralsetinib exposures and pharmacodynamics modulation of tumor biomarkers, including direct inhibition of RET activity. Pralsetinib has been shown to be tolerable at active doses in in vivo pharmacology and safety pharmacology studies. Given the strong genetic and preclinical evidence that activated RET is an oncogenic disease driver and the poor prognosis of many patients with RET-driven tumors, the potential benefit/risk profile of pralsetinib suggests study in humans is warranted. This study is the first study to evaluate the maximum tolerated dose and investigate clinical activity of pralsetinib.
Number of Patients:	Approximately 647 patients will be enrolled in this study, including:<br>• A total of 62 patients were enrolled in Phase 1 (Dose Escalation)<br>• Approximately 585 patients in Phase 2 (Dose Expansion)
Objectives:	**Phase 1, Dose Escalation Objectives**<br><u>Primary Objectives</u><br>• To determine the maximum tolerated dose (MTD) and recommended Phase 2 dose (RP2D) of pralsetinib.<br>• To determine the safety and tolerability of pralsetinib.<br><u>Secondary Objectives</u><br>• To determine the overall response rate (ORR) by (Response Evaluation Criteria in Solid Tumors) RECIST v1.1 (or Response Assessment in Neuro-Oncology [RANO], if appropriate for tumor type) according to

patients' disease type, and/or RET-altered status if applicable, and/or prior treatment status, if appropriate.

- To assess baseline RET gene status in plasma and/or tumor tissue and correlate with measures of antineoplastic activity including, but not limited to ORR, clinical benefit rate (CBR), disease control rate (DCR), and duration of response (DOR).
- To characterize the pharmacokinetic (PK) profile of pralsetinib and correlate drug exposure with safety assessments.
- To characterize the pharmacodynamics of pralsetinib, including, but not limited to, changes in blood calcitonin and carcinoembryonic antigen (CEA) in MTC patients only, and changes in tumor dual specificity phosphatase 6 (DUSP6) and sprout receptor tyrosine kinase (RTK) signaling antagonist 4 (SPRY4) levels in all patients.

<u>Exploratory Objectives</u>

- To identify potential new blood and tumor tissue biomarkers (eg, deoxyribonucleic acid [DNA], ribonucleic acid [RNA], and/or protein markers) of pharmacodynamic activity, antineoplastic activity, and/or toxicity.

Phase 2, Dose Expansion Objectives

<u>Primary Objectives</u>

- To determine the ORR by RECIST v1.1 (or RANO, if appropriate for tumor type) according to patients' disease type, and/or RET-altered status if applicable, and/or prior treatment status if appropriate.
- To further define the safety and tolerability of pralsetinib.

<u>Secondary Objectives</u>

- To assess additional measures of clinical benefit including DOR, CBR, DCR, PFS, and overall survival (OS) in all patients according to patients' disease type, and/or RET-altered status if applicable, and/or prior treatment status if appropriate.
- To assess baseline RET gene status (ie, gene fusion partner or primary mutation and, for MTC, whether hereditary or sporadic) in plasma and/or tumor tissue and correlate with measures of antineoplastic activity including, but not limited to ORR, CBR, DOR, and DCR.
- To characterize the PK profile of pralsetinib and correlate drug exposure with safety assessments, including changes in ECG intervals, and efficacy.
- To characterize the pharmacodynamics of pralsetinib, including, but not limited to, changes in blood calcitonin and CEA in MTC patients only.
- To assess brain activity in patients with NSCLC.

<u>Exploratory Objectives</u>

- To identify potential new blood and tumor tissue biomarkers (eg, DNA, RNA, and/or protein markers) of pharmacodynamic activity, antineoplastic activity, and/or toxicity.
- To assess changes in quality of life (QoL) questionnaire.

- To explore disease-related symptoms, as measured by bowel movement history (MTC patients only).
- To explore clinical benefit including ORR, CBR, DCR, PFS for patients previously treated with a selective RET tyrosine kinase inhibitor.
- To assess brain activity in patients with tumor types other than NSCLC.

Study Design: This is a Phase 1/2, open-label, first-in-human (FIH) study designed to evaluate the safety, tolerability, PK, pharmacodynamics, and antineoplastic activity of pralsetinib, a potent and selective RET inhibitor, administered orally in patients with medullary thyroid cancer, RET-altered NSCLC and other RET-altered solid tumors.

The study consists of 2 parts, a dose-escalation part (Phase 1) and an expansion part (Phase 2). Both parts will enroll patients with advanced non-resectable NSCLC, advanced non-resectable thyroid cancer and other advanced non-resectable solid tumors. Prior to Amendment 9, all patients were required to have progressed following standard systemic therapy, have not adequately responded to standard systemic therapy, or be intolerant to or the Investigator has determined that treatment with standard therapy is not appropriate, or there must be no accepted standard therapy for their disease.

The dose-escalation portion of the study (Phase 1) completed on 03 April 2018. It employed the Bayesian optimal interval (BOIN) design to find the MTD of pralsetinib. Dose escalation continued to enroll in cohorts of 3-6 (1-3 for the first 3 dose levels) patients until 12 patients were treated and evaluable for dose-limiting toxicity (DLT) at one dose level, at which time the MTD or RP2D could be determined. The total number of patients to be enrolled during the dose escalation part of the study could vary depending on the toxicity profile of pralsetinib and the number of dose levels tested prior to reaching the MTD.

All patients treated at doses > 120 mg per day were required to have MTC, or a RET-altered solid tumor per local assessment of tumor tissue and/or blood. Additionally, these patients could be enrolled into an enrichment cohort, if it previously included less than 12 patients evaluable for DLT, was reviewed at a dose-escalation meeting, and did not exceed the MTD. Data from these patients allows for an improved assessment of safety, PK, and pharmacodynamics.

In Phase 2, patients will enroll into 1 of 9 groups based on their tumor type and prior therapy status (if applicable):

- Group 1: NSCLC with a RET fusion previously treated with a platinum-based chemotherapy (N ~80).
- Group 2: NSCLC with a RET fusion <u>not</u> previously treated with a platinum-based chemotherapy, including those who have not had any systemic therapy. Prior platinum chemotherapy in the neoadjuvant and adjuvant setting is permitted if the last dose of platinum was 4 months or more before the first dose of study drug (N~200, including approximately 30 patients with prior systemic therapy).
- Group 3: MTC previously treated with cabozantinib and/or vandetanib (N ~65).
- Group 4: MTC <u>not</u> previously treated with cabozantinib or vandetanib (N ~40)

- Group 5: Other solid tumors with a RET fusion not eligible for any of the other groups (N ~100). Patients should have previously received SOC appropriate for their tumor type, unless there is no accepted standard therapy for the tumor type or the Investigator has determined that treatment with standard therapy is not appropriate. As the intent of this cohort is to enroll a variety of RET-fusion tumor types, Blueprint will notify sites if/when sufficient data are available and accrual should cease for a particular tumor type.
- Group 6: Any solid tumors with a RET alteration (fusion or mutation) previously treated with a selective RET tyrosine kinase inhibitor (TKI) (N ~20).
- Group 7: Other solid tumors with a RET mutation previously treated with SOC appropriate for the tumor type (N ~20).
- Group 8: NSCLC with a RET fusion previously treated with a platinum-based chemotherapy (China only; N ~30)
- Group 9: MTC not previously treated with systemic therapy (except prior cytotoxic chemotherapy is allowed) for advanced or metastatic disease extension cohort (China only; N ~30)

Determination of RET status, as required for enrollment of all patients except those with MTC, is based on local assessment, or central assessment if local testing is not available. All patients enrolled in Phase 2 (all 9 groups) must submit tumor tissue (archived or new) for retrospective assessment of RET status and other pathway biomarkers. Patients enrolled into Group 6 (previous treatment with a selective RET-inhibitor) are required to have a new tumor biopsy prior to enrollment.

All study visits are intended to be conducted on an outpatient basis, but may be conducted on an inpatient basis, as needed. After provision of written informed consent (within 8 weeks before study drug administration), patients will be evaluated for study eligibility during the Screening period within 28 days before study drug administration on Cycle 1, Day 1 (C1D1).

On C1D1, eligible patients will present to the study center approximately 2 hours before the first dose of study drug and will remain at the study center for at least 8 hours for serial PK sampling, pharmacodynamic sample collection, vital signs measurement, electrocardiogram (ECG) monitoring, safety laboratory tests, and safety monitoring. In Phase 2, patients will complete a QoL assessment (European Organization for Research and Treatment of Cancer Core Quality of Life Questionnaire [EORTC QLQ-C30]). Additionally, in Phase 2 only, continuous ECG Holter monitoring will be performed approximately 1 hour before dosing until after collection of the 8-hour PK sample for approximately 20 evaluable patients at select study centers. At these study centers, replicate 12 lead ECGs will be extracted from the continuous recording at the same time points as the PK blood draws. Approximately 72 patients (including ~12 patients in Groups 8 and 9) in Phase 2 will complete the serial PK sampling during Cycle 1; otherwise, patients enrolled in Phase 2 will only have predose PK samples collected. See Table 10, Table 11, and Table 12 and Section 7 for a detailed description of study assessments.

	A treatment cycle is 28 days in duration. Initially, patients returned to the study center on C1D2 for PK sampling (24 hours after the C1D1 dose), C1D8 and C1D22 for safety monitoring, and on C1D15 and C1D16 for serial PK sampling and safety monitoring (including continuous Holter monitoring for 20 patients in Phase 2 at select study centers). Following Protocol Amendment 9, visits on C1D2 and C1D16 are no longer required, except for patients in Groups 8 and 9, who will undergo serial PK sampling in China according to the country-specific Protocol Amendment 10. Patients will also attend study center visits on C2D1 and C2D15, on Day 1 of C3 through C13, C15, C17, C21, and every 4 cycles thereafter for additional safety monitoring; PK and pharmacodynamic sampling; and disease response assessment by computed tomography (CT) or magnetic resonance imaging (MRI). In Phase 2 only, the EORTC QLQ-C30 will be administered on D1 of every cycle through C12. A tumor biopsy will be performed within 2 weeks (±) of C2D1 (Phase 1 only) and upon disease progression (both Parts), if the patient consents and the procedure is deemed to be safe and medically feasible by the Investigator.<br><br>All patients will attend an End-of-treatment (EOT) visit approximately 14 days (±7 days) after the last dose of study drug. A Follow-up telephone contact for resolution of any residual adverse event (AE) will be made on Day 30 (+7 days) after the last dose of study drug, or at the time the patient initiates another antineoplastic therapy. All patients will subsequently be contacted every 3-4 months for PFS (until progression or start of new therapy) and every 3 months for overall survival.<br><br>In the event that, due to unforeseen circumstances, a patient is unable to travel to the investigative site for a planned study visit, elements of that visit may be performed remotely (eg, phone/video calls) or at outside facilities with notification and agreement from the Sponsor. Appropriate guidance documents may be put in place to support these unforeseen circumstances. All efforts should be made to ensure the safety and wellbeing of the patient prior to continuation of study drug and altered study visits (as above) should be appropriately documented in the source.
Duration of Treatment:	It is anticipated that patients will receive at least 1 cycle of pralsetinib; no maximum treatment duration has been set. After C1, patients may continue to receive pralsetinib until precluded by toxicity, noncompliance, withdrawal of consent, death, or closure of the study by the Sponsor.
Duration of Patient Participation:	The minimum duration of patient participation is approximately 3 months, including a screening period to assess study eligibility up to 4 weeks (28 days); a treatment period of at least 1 cycle (28 days); an EOT visit at least 14 ± 7 days following the last dose of study drug; and a Follow-up telephone contact for resolution of any AEs 30 days (+ 7 days) after the last dose of study drug, or at the time the patient initiates another antineoplastic therapy; and PFS and OS follow-up until withdrawal of consent, death, or loss to follow-up.
Duration of Study:	The expected enrollment period is approximately 60 months and the expected duration of the study is approximately 84 months.
Target Population:	Key Inclusion Criteria:<br>• Patient is ≥ 18 years of age.

- Diagnosis during dose escalation (Phase 1) – Pathologically documented, definitively diagnosed non-resectable advanced solid tumor.
  - All patients treated at doses > 120 mg per day must have MTC, or a RET-altered solid tumor per local assessment of tumor tissue and/or blood.
  - Phase 1 enrichment patients must have MTC or a RET-altered solid tumor per local assessment of tumor tissue and/or blood.
- Diagnosis during dose expansion (Phase 2) – All patients (with the exception of patients with MTC enrolled in Groups 3, 4, and 9) must have an oncogenic RET fusion or mutation (excluding synonymous, frameshift, and nonsense mutations) solid tumor, as determined by local testing of tumor or circulating tumor nucleic acid in blood; as detailed below.
  - Group 1 – patients must have pathologically documented, definitively diagnosed locally advanced or metastatic NSCLC with a RET fusion previously treated with a platinum-based chemotherapy.
  - Group 2 – patients must have pathologically documented, definitively diagnosed locally advanced or metastatic NSCLC with a RET fusion not previously treated with a platinum-based chemotherapy, including those who have not had any systemic therapy. Prior platinum chemotherapy in the neoadjuvant and adjuvant setting is permitted if the last dose of platinum was 4 months or more before the first dose of study drug.
  - Group 3 – patients must have pathologically documented, definitively diagnosed advanced MTC that has progressed within 14 months prior to the Screening Visit and was previously treated with cabozantinib and/or vandetanib.
  - Group 4 – patients must have pathologically documented, definitively diagnosed advanced MTC that has progressed within 14 months prior to the Screening Visit and was not previously treated with cabozantinib or vandetanib.
  - Group 5 - patients must have a pathologically documented, definitively diagnosed advanced solid tumor with an oncogenic RET fusion, have previously received SOC appropriate for their tumor type (unless there is no accepted standard therapy for the tumor type or the Investigator has determined that treatment with standard therapy is not appropriate), and must not eligible for any of the other groups.
  - Group 6 - patients must have a pathologically documented, definitively diagnosed advanced solid tumor with an oncogenic RET fusion or mutation, previously treated with a selective TKI that inhibits RET, such as selpercatinib.
  - Group 7 - patients must have a pathologically documented, definitively diagnosed advanced solid tumor with an oncogenic RET mutation previously treated with SOC appropriate for the tumor type and not eligible for any of the other groups.
  - Group 8 - patients must have pathologically documented, definitively diagnosed locally advanced or metastatic NSCLC with a RET fusion that was previously treated with a platinum-based chemotherapy (China only).

- Group 9 - patients must have pathologically documented, definitively diagnosed advanced MTC that has progressed within 14 months prior to the Screening Visit, and was not previously treated with systemic therapy (except prior cytotoxic chemotherapy is allowed) for advanced or metastatic disease (China only).

- Patients must have non-resectable disease. Prior to Protocol Amendment 9, patients must have progressed following standard therapy or have not adequately responded to standard therapy, or the patient must be intolerant to or the Investigator has determined that treatment with standard therapy is not appropriate, or there must be no accepted standard therapy for their disease.
- Dose expansion (Phase 2) patients in all groups (except Group 7) must have measurable disease per RECIST v1.1 (or RANO if appropriate).
- Patient agrees to provide tumor tissue (archived, if available or a fresh biopsy) for RET status confirmation and is willing to consider an on-treatment tumor biopsy, if considered safe and medically feasible by the treating Investigator. For Phase 2, Group 6, patients are required to undergo a pretreatment biopsy to define baseline RET status in tumor tissue.
- Patient has Eastern Cooperative Oncology Group (ECOG) performance status (PS) of 0-1.

Primary Endpoints:

Phase 1, Dose Escalation

- MTD and RP2D of pralsetinib.
- Overall safety profile of pralsetinib, as assessed by the type, frequency, severity, timing, and relationship to study drug of any AEs, serious adverse events (SAEs), changes in vital signs, ECGs, and safety laboratory tests.

Phase 2, Dose Expansion

- ORR by disease type, and/or RET-altered status if applicable, and/or prior treatment status, if appropriate, including patients treated at the MTD/RP2D in Phase 1.
- Overall safety profile of pralsetinib, as assessed by the type, frequency, severity, timing, and relationship to study drug of any AEs, SAEs, changes in vital signs, ECGs, and safety laboratory tests.

Secondary Endpoints:

Phase 1, Dose Escalation

- ORR for all patients according to patients' disease type and/or RET-altered status if applicable, and/or prior treatment status, if appropriate.
- RET gene status and correlation between RET gene status and ORR, DOR, CBR, and DCR, and other measures of antineoplastic activity (in plasma and/or tissue) in all patients according to their disease type and/or RET-altered status, if applicable, and/or prior treatment status, if appropriate.
- PK parameters of pralsetinib: Pharmacokinetic parameters of interest will include, as appropriate, maximum plasma drug concentration (C_{max}), time to maximum plasma drug concentration (T_{max}), time of last quantifiable

plasma drug concentration (T_{last}), area under the plasma concentration versus time curve from time 0 to 24 hours postdose (AUC_{0-24}), plasma drug concentration at 24 hours postdose (C_{24}); apparent volume of distribution (V_z/F), terminal elimination half-life ($t_{½}$), apparent oral clearance (CL/F), and accumulation ratio (R).

- Pharmacodynamic parameters of pralsetinib: changes in tumor/blood including, but not limited to, changes in blood calcitonin and CEA (MTC patients); and changes in tumor biomarker levels (DUSP6 and SPRY4) levels (all patients).

Phase 2, Dose Expansion

- DOR, CBR, DCR, PFS, and OS in all patients, including patients treated at MTD/RP2D in Phase 1, according to their disease type and/or RET-altered status, if applicable, and/or prior treatment status, if appropriate.
- RET gene status (ie, gene fusion partner or primary mutation and, for MTC, whether hereditary or sporadic) and correlation between RET gene status and ORR, DOR, CBR, DCR, and other measures of antineoplastic activity (in plasma and/or tissue) in all patients, including patients treated at MTD/RP2D in Phase 1, according to their disease type and/or RET-altered status if applicable, and/or prior treatment status, if appropriate.
- PK parameters of pralsetinib: PK parameters of interest will include, as appropriate, maximum plasma drug concentration (C_{max}), time to maximum plasma drug concentration (T_{max}), time of last quantifiable plasma drug concentration (T_{last}), area under the plasma concentration versus time curve from time 0 to 24 hours postdose (AUC_{0-24}), plasma drug concentration at 24 hours postdose (C_{24}); apparent volume of distribution (V_z/F), terminal elimination half-life ($t_{½}$), apparent oral clearance (CL/F), and accumulation ratio (R).
- ECG Assessment: The effects of pralsetinib on ECG parameters will be evaluated for approximately 20 evaluable patients using 12-lead ECGs extracted from continuous recordings (12-lead Holter) on C1D1 and C1D15. Individual ECGs will be extracted in replicate from the 12-lead Holter recordings at specified time points and will be evaluated by a central laboratory. QT intervals will be measured from lead II and will be corrected for heart rate (QTc) using Fridericia's correction factors (QTcF). The primary QTc parameter will be QTcF. Secondary parameters (heart rate, PR, and QRS, and T-wave morphology) will also be evaluated. Potential effects of pralsetinib will be evaluated as change from predose baseline heart rate, PR, QRS, and QTcF by postdose time point. For the purpose of QT assessment, exposure-response analysis will be performed on the relationship between pralsetinib systemic levels and change in QTcF.
- Pharmacodynamic parameters of pralsetinib: changes in tumor/blood including, but not limited to, changes in blood calcitonin and CEA and biochemical response rate (MTC patients only).
- Intracranial response rate among patients with NSCLC who have measurable (target by RECIST v1.1) lesions in the brain, and time to intracranial progression among all patients with NSCLC.

Exploratory Endpoints	**Phase 1, Dose Escalation**<br>• Levels of exploratory blood and tumor markers (DNA, RNA and protein) as compared to antineoplastic activity, and/or toxicity.<br>**Phase 2, Dose Expansion**<br>• Changes in patient-reported outcomes as assessed by the EORTC QLQ-C30 instruments.<br>• Changes in disease-related symptoms as reported by bowel movement history for MTC patients.<br>• ORR, CBR, DCR, PFS for patients previously treated with a selective RET tyrosine kinase inhibitor.<br>• Intracranial response rate among patients with tumor types other than NSCLC who have measurable (target by RECIST v1.1) lesions in the brain, and time to intracranial progression among all patients with tumor types other than NSCLC.

STUDY SCHEMATIC

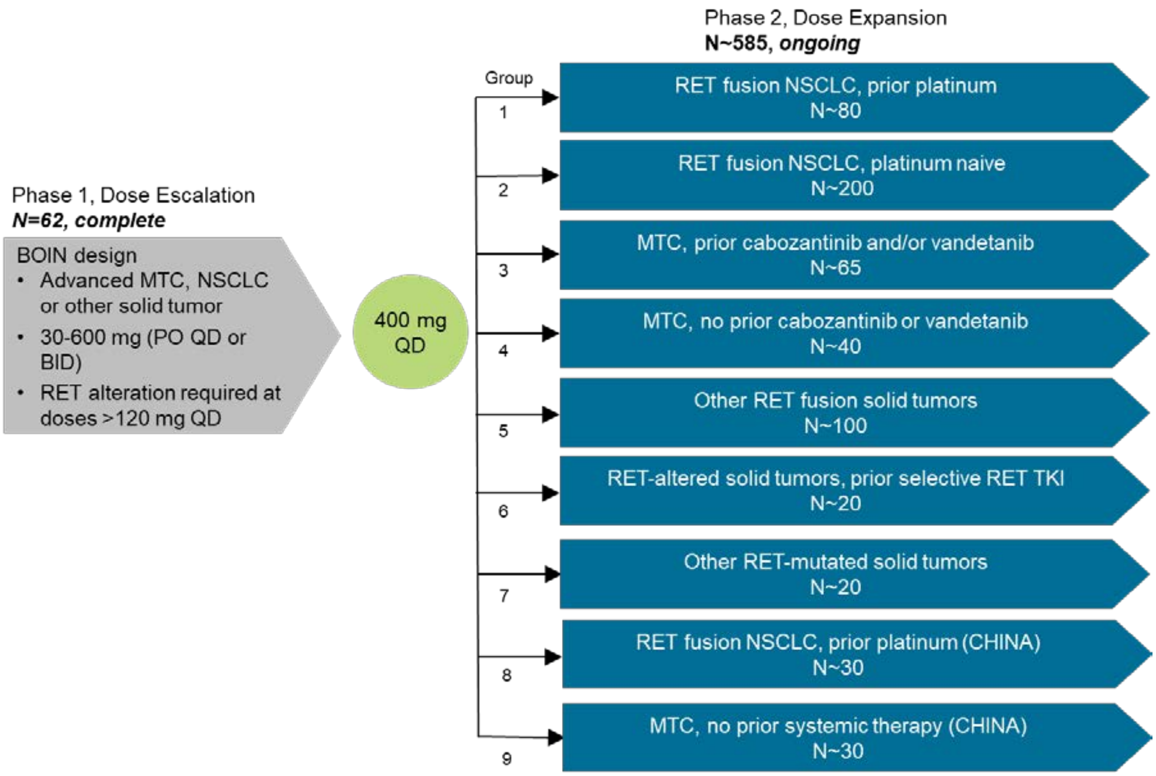

Abbreviations: BID = twice daily; BOIN = Bayesian optimal interval; MKI = multikinase inhibitor; MTD = Maximum tolerated dose; MTC = medullary thyroid cancer; NSCLC = non-small cell lung cancer; PO = per oral; QD = once daily; RET = rearranged during transfection; RP2D = recommended Phase 2 dose; TKI = tyrosine kinase inhibitor

TABLE OF CONTENTS

CLINICAL RESEARCH PROTOCOL 1
CLINICAL PROTOCOL....2
AMENDMENT 13 RATIONALE 3
AMENDMENT 12 RATIONALE 5
AMENDMENT 11 RATIONALE 6
AMENDMENT 10 RATIONALE 7
AMENDMENT 9 RATIONALE 8
AMENDMENT 8 RATIONALE 10
AMENDMENT 7 RATIONALE 11
AMENDMENT 6 RATIONALE 13
AMENDMENT 5 RATIONALE 13
AMENDMENT 4.1 RATIONALE 13
AMENDMENT 3 RATIONALE 15
AMENDMENT 2 RATIONALE 15
AMENDMENT 1 RATIONALE 16
STUDY SUMMARY....17
TABLE OF CONTENTS 27
LIST OF TABLES 30
LIST OF FIGURES 31
LIST OF ABBREVIATIONS 32
1 BACKGROUND AND RATIONALE 35
1.1 Background on RET and RET-driven Cancers....35
1.1.1 RET-altered Thyroid Cancer....36
1.1.2 RET-altered NSCLC 38
1.1.3 Other RET-driven Cancers....38
1.2 Pralsetinib (previously known as BLU-667)....39
1.2.1 Preclinical Pharmacology....39
1.2.2 Toxicology....40
1.3 Study Rationale and Design 42
1.4 Rationale for the Starting Dose and Dosing Regimen 44
1.5 BLU-667-1101 Study Update 44
1.5.1 Phase 1 Dose Escalation Results 45
1.5.2 Phase 2 Dose Expansion Preliminary Data 48
1.5.3 Overall Safety....50
1.5.4 Overall Benefit-Risk Assessment....52
2 STUDY OBJECTIVES 52
2.1 Phase 1 Objectives....52
2.1.1 Primary Objectives 52
2.1.2 Secondary Objectives....52
2.1.3 Exploratory Objectives....53
2.2 Phase 2 Objectives....53
2.2.1 Primary Objectives 53
2.2.2 Secondary Objectives....53
2.2.3 Exploratory Objectives....54
3 STUDY ENDPOINTS 54

3.1 Phase 1 Endpoints 54
3.1.1 Primary Endpoints 54
3.1.2 Secondary Endpoints 54
3.1.3 Exploratory Endpoints 55
3.2 Phase 2 Endpoints 55
3.2.1 Primary Endpoints 55
3.2.2 Secondary Endpoints 55
3.2.3 Exploratory Endpoints 56
4 STUDY PLAN 56
4.1 Study Design 56
4.1.1 Phase 1 (Dose-escalation) 59
4.1.2 Phase 2 (Expansion) 60
5 POPULATION 61
5.1 Number of Patients 61
5.2 Inclusion Criteria 61
5.3 Exclusion Criteria 63
5.4 RET Pre-screening 65
5.5 Patient Identification and Registration 65
5.6 Study Completion 66
5.6.1 Patient Withdrawal Criteria from Study Treatment 66
5.6.2 Patient Withdrawal Criteria from the Study 66
5.7 Replacement of Patients 67
6 STUDY CONDUCT 67
6.1 General Conduct 67
6.2 Early Termination 68
6.2.1 Rules for Early Termination of Enrollment 68
6.3 Dose and Administration 69
6.3.1 Dosing for Phase 1 (Dose Escalation) 70
6.3.2 Dosing for Phase 2 (Dose Expansion) 76
6.4 Prior and Concomitant Therapy 81
6.4.1 Prohibited Concomitant Therapy 81
6.4.2 Concomitant Therapy to be used with Caution 82
6.4.3 Permitted Concomitant Therapy 82
6.5 Additional precautions 82
6.6 Schedule of Assessments 83
7 DESCRIPTION OF STUDY PROCEDURES 89
7.1 RET pre-screening 89
7.2 Screening 89
7.3 Safety Assessments 90
7.3.1 Physical Examination 90
7.3.2 Eastern Cooperative Oncology Group Performance Status 90
7.3.3 Vital Signs 90
7.3.4 ECGs 90
7.3.5 Clinical Laboratory Tests 91
7.3.6 Adverse Events and Concomitant Medications 91
7.3.7 Thyroid Hormone Replacement in Patients with Thyroid Cancer 92

7.4 Pharmacokinetic Assessment 92
7.5 Pharmacodynamic and Biomarker Assessment 92
7.5.1 Blood Samples for Pharmacodynamic and Biomarker Assessment 93
7.5.2 Tumor Samples for RET Status and Biomarker Assessment 93
7.6 Disease Response Assessment 94
7.7 Patient-Reported Outcomes and Quality of Life Measurements 94
7.8 End-of-treatment and 30-Day Safety Follow-up 94
7.9 Progression Free Survival and Overall Survival Follow-up 95
7.10 Sample Processing, Storage, and Shipment 95
8 STUDY DRUG MANAGEMENT 95
8.1 Description 95
8.1.1 Formulation 95
8.1.2 Storage 95
8.2 Packaging and Shipment 96
8.3 Accountability 96
8.4 Compliance 96
9 ADVERSE EVENTS 97
9.1 Definitions 97
9.1.1 Definition of Adverse Event 97
9.1.2 Suspected Adverse Reaction 97
9.1.3 Unexpected Adverse Event 98
9.2 Documenting Adverse Events 98
9.3 Assessment of Intensity 98
9.4 Assessment of Causality 99
9.5 Pretreatment Events 99
9.6 Pregnancy Reporting 99
9.6.1 Contraception Requirements 100
9.6.2 Gamete and Embryo Banking 101
10 SERIOUS ADVERSE EVENTS 101
10.1 Definition of Serious Adverse Event 101
10.2 Reporting Serious Adverse Events 102
10.2.1 Overdose 103
11 STATISTICS 103
11.1 General Procedures 103
11.2 Analysis Populations 104
11.3 Sample Size 105
11.3.1 Phase 1 (Dose-escalation) 105
11.3.2 Phase 2 (Expansion) 106
11.4 Statistical Methods 107
11.4.1 Demographic and Baseline Characteristics 108
11.4.2 Primary Endpoints 108
11.4.3 Secondary Endpoints 112
11.4.4 Exploratory Endpoints 116
12 ETHICS AND RESPONSIBILITIES 116
12.1 Good Clinical Practice 116
12.2 Data and Safety Monitoring 117

12.2.1 Phase 1 Data and Safety Monitoring 117
12.2.2 Phase 2 Data and Safety Monitoring 117
12.3 Institutional Review Board/Independent Ethics Committee 119
12.4 Informed Consent 119
12.5 Records Management 120
12.6 Source Documentation 120
12.7 Study Files and Record Retention 120
12.8 Liability and Insurance 121
13 AUDITING AND MONITORING 121
14 AMENDMENTS 121
15 STUDY REPORT AND PUBLICATIONS 122
16 STUDY DISCONTINUATION 122
17 CONFIDENTIALITY 122
18 REFERENCES 123
19 APPENDICES 126
19.1 Appendix 1: Eastern Cooperative Oncology Group Performance Status 126
19.2 Appendix 2: List of Prohibited Medications and Food 127
19.3 Appendix 3: List of Medications and Food to be Used with Caution 128
19.4 Appendix 4: Operating Characteristics of the BOIN Design 129
19.5 Appendix 5: Investigator Signature Page 132
19.6 Appendix 6: Common Oncogenic RET Mutations 133
19.7 Appendix 7: EORTC QLQ-C30 Assessment 134
19.8 Appendix 8: Examples of Implementing the Early Stopping Rule 136

LIST OF TABLES

Table 1: Biochemical Activity of Cabozantinib, Vandetanib, Regorafenib, Ponatinib, and Pralsetinib against Wild Type RET, RET Mutants, and RET Fusion Proteins 39
Table 2: Pralsetinib Dose Levels and Number of Patients Enrolled 45
Table 3: AEs Occurring in ≥15% of Patients Regardless of Relationship in Study BLU-667-1101 (N=421) 51
Table 4: Serious Adverse Events Considered Related to Study Drug Occurring in >1 Patient (N=421) 51
Table 5: Dose Escalation/De-escalation Rule for the BOIN Design 71
Table 6: Provisional Dose-escalation Levels 74
Table 7: Dose Modification Guidelines for Pralsetinib-Related Toxicity 77
Table 8: Pralsetinib Dose Modification Recommendations for Treatment-related Pneumonitis 78
Table 9: Pralsetinib Dose Modification Recommendations for Treatment-related Hyperphosphatemia 80
Table 10: Schedule of Assessments 83
Table 11: Schedule for Pharmacokinetic Sample Collection and ECG Monitoring 87
Table 12: Schedule for Pharmacodynamic Biomarker Sample Collection 88

Table 13: Confidence Interval of Observed Response Rate for Group 1, N = 80 109
Table 14: Confidence Interval of Observed Response Rate for Group 2, N=170 109
Table 15: Confidence Interval of Observed Response Rate for Group 3, N = 65 110
Table 16: Confidence Interval of Observed Response Rate for Group 4, N = 40 110
Table 17: Probability of Observing Responses for Group 5, N = 100 110
Table 18: Confidence Interval of Observed Response Rate for Group 5, N = 100 111
Table 19: Probability of Observing Responses for Groups 6 and 7, N = 20 111
Table 20: Confidence Interval of Observed Response Rate for Groups 8 and 9, N = 30 112

LIST OF FIGURES

Figure 1: RET Signaling Pathway 35
Figure 2: Spectrum of Oncogenic RET Alterations 37
Figure 3: Efficacy and Body Weight Measurements with Pralsetinib (BLU-667) in a KIF5B-RET NSCLC Patient-Derived Xenograft Model 40
Figure 4: Mean (+ SD) Plasma Concentration Profiles of Pralsetinib following Pralsetinib on C1D1 (Single Dose) and at C1D15 (Steady State) Following Once Daily Oral Administration (Dose Escalation) 47
Figure 5: Antitumor Activity of Pralsetinib in RET fusion-positive NSCLC 49
Figure 6: Anti-tumor Activity of Pralsetinib in MTC Patients Irrespective of Prior Therapy 50
Figure 7: Study Schematic 58
Figure 8: Dose-Escalation Schematic 72
Figure 9: Dose-toxicity Scenarios 106

LIST OF ABBREVIATIONS

Abbreviation	Definition
AE	adverse event
ALK	anaplastic lymphoma kinase
ALT	alanine aminotransferase
ANC	absolute neutrophil count
AST	aspartate aminotransferase
AUC_{0-24}	area under the plasma concentration versus time curve from 0 to 24 hours
AUC_{inf}	area under the plasma concentration versus time curve from 0 to infinity
β-hCG	beta human chorionic gonadotropin
BID	twice daily
BOIN	Bayesian optimal interval
CBR	clinical benefit rate
CEA	carcinoembryonic antigen
CI	confidence interval
C_{max}	maximum plasma concentration
CNS	central nervous system
CR	complete response
CRM	continual reassessment method
CT	computed tomography
CTCAE	Common Terminology Criteria for Adverse Events
CTFG	Clinical Trial Facilitation Group
ctDNA	circulating tumor deoxyribonucleic acid
CxDx	Cycle x Day x
CYP	Cytochrome P450
DLT	dose-limiting toxicity
DNA	deoxyribonucleic acid
λ_e	dose escalation boundary
λ_d	dose de-escalation boundary
DCR	disease control rate
DOR	duration of response
DTC	differentiated thyroid cancer
DUSP6	dual specificity phosphatase 6

Abbreviation	Definition
ECG	electrocardiogram
ECOG	Eastern Cooperative Oncology Group
eCRF	electronic case report form
EORTC	European Organization for Research and Treatment of Cancer
EOT	End-of-treatment
FIH	first-in-human
FSH	follicle-stimulating hormone
GCP	Good Clinical Practice
GLP	Good Laboratory Practice
GDNF	glial cell line-derived neurotrophic factors
GFRα	GDNF family receptors-α
HNSTD	highest non-severely toxic dose
IB	Investigator’s Brochure
ICF	informed consent form
ICH	International Conference on Harmonization
IDMC	Independent Data Monitoring Committee
IEC	Independent Ethics Committee
IRB	Institutional Review Board
IV	intravenous
JAK2	Janus kinase 2
MKI	multikinase inhibitor
MRI	Magnetic resonance imaging
MTC	medullary thyroid cancer
MTD	maximum tolerated dose
NCCN	National Comprehensive Cancer Network
NCI	National Cancer Institute
NSCLC	non-small cell lung cancer
ORR	overall response rate
OS	overall survival
PDX	patient-derived xenograft
P-gp	P-glycoprotein
PFS	progression-free survival
PK	pharmacokinetic(s)
PR	partial response

**Abbreviation**	**Definition**
PS	performance status
QD	once daily
QTcF	QT interval corrected using Fridericia's formula
RAI	radioactive iodine
RANO	Response Assessment in Neuro-Oncology
RECIST	Response Evaluation Criteria in Solid Tumors
RET	rearranged during transfection
RNA	ribonucleic acid
RP2D	recommended Phase 2 dose
RTK	receptor tyrosine kinase
SAE	serious adverse event
SOC	standard of care
SPRY4	sprout RTK signaling antagonist 4
SRC	Safety Review Committee
STD_{10}	severely toxic dose in 10% of animals
$t_{1/2}$	terminal half-life
T_4	thyroxine
TKI	tyrosine kinase inhibitor
TSH	thyroid-stimulating hormone
TLS	tumor lysis syndrome
ULN	upper limit of normal
VEGFR	vascular endothelial growth factor receptor

1 BACKGROUND AND RATIONALE

1.1 Background on RET and RET-driven Cancers

Increasing evidence implicates aberrant activation of the receptor tyrosine kinase (RTK), RET (rearranged during transfection), as a critical driver of tumor growth and proliferation across a broad number of solid tumors (Mulligan, 2014). RET along with glial cell line-derived neurotrophic factors (GDNF) and GDNF family receptors-α (GFRα), normally mediates development, maturation and maintenance of several neural, neuroendocrine and genitourinary tissue types. The RET signaling pathway is shown below in Figure 1. In contrast, oncogenic RET activation occurs via gain of function mutation which constitutively activates RET and promotes ligand-independent tumor growth. Oncogenic RET activation was initially described in hereditary and sporadic thyroid cancers and subsequently in non-small cell lung cancer (NSCLC) adenocarcinoma. However, as next generation deoxyribonucleic acid (DNA) sequencing has become more prevalent, it has become apparent that oncogenic RET mutations occur in a variety of solid tumors.

Figure 1: RET Signaling Pathway

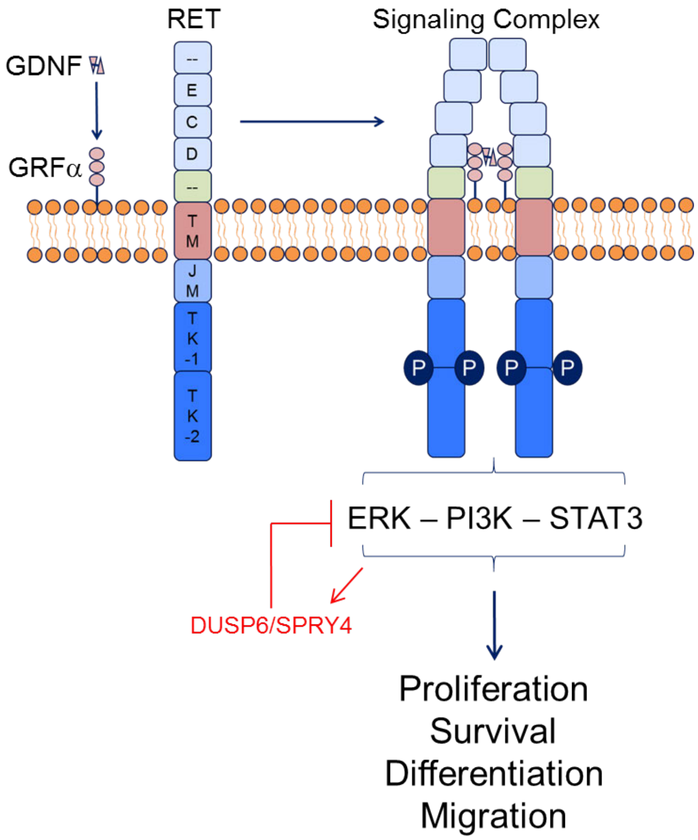

Abbreviations: GDNF = Glial cell line-derived neurotrophic, GRFα = Glial cell line-derived neurotrophic factor alpha. RET = Rearranged during transfection, ERK = Extracellular signal-related kinase, PI3K = phosphotidylinositol-3-kinase, STAT3 = Signal transducer and activator of transcription 3, DUSP6 = dual specificity phosphatase 6, SPRY4 = sprout RTK signaling antagonist 4

Binding of GDNF-GRFα to RET promotes receptor dimerization and activation of RET kinase activity, which initiates downstream signaling and phenotypic effects via the ERK, PI3K and STAT3 pathways. Feedback inhibition of ERK signaling occurs via DUSP6 and SPRY4.

1.1.1 RET-altered Thyroid Cancer

Thyroid cancer, which consists primarily of differentiated thyroid cancer (DTC; ~90% of cases), medullary thyroid cancer (MTC; ~5% of cases) and anaplastic thyroid cancer (< 5% of cases), is the most common endocrine malignancy with ~64,000 new cases per year and ~2,000 deaths in the United States (US) (SEER). DTC arises sporadically from thyroid follicular cells and consists of papillary thyroid cancer (PTC) (~85% of cases) and follicular thyroid cancer (FLC) (~5-10%). In contrast, MTC arises from parafollicular C cells and occurs in both hereditary and sporadic forms. Oncogenic RET activation has been implicated as a driver in both MTC and DTC.

Recurrent gene rearrangements involving RET and a dimerization domain-encoding gene have been identified in ~10% of sporadic papillary tumors in adults and ~50-80% of papillary tumors that occur after radiation exposure or arise in childhood (Figure 2). The dimerization domain provided by the fusion partner allows constitutive association of RET kinase domains, leading to aberrant kinase activation that promotes tumorigenesis. Kinase-activating RET alterations also occur in nearly all cases of hereditary and approximately 50% of sporadic MTC. RET alterations are found in about 90% of cases of unresectable or metastatic MTC (Romei et al, 2018). Hereditary MTC is associated with 3 subtypes of Multiple Endocrine Neoplasia (MEN) Syndrome Type 2 (MEN2), which are variably associated with other endocrine tumors (eg, MEN2A – MTC, pheochromocytoma, parathyroid hyperplasia; MEN2B – MTC, pheochromocytoma, marfinoid habitus, neurofibromatosis; FMTC – MTC without pheochromocytoma or parathyroid hyperplasia). RET alterations occur in > 95% of hereditary MTC and these include extracellular domain mutations (primarily at the C634 position) which promote ligand-independent activation of RET, and kinase domains mutations (primarily M918T, A883F or V804L/M) which promote RET auto-activation and consequent oncogenic signaling (Romei et al, 2016). RET mutations are also oncogenic drivers in approximately 50% of sporadic MTC, especially the M918T mutation as it accounts for ~70% of the RET alterations in sporadic MTC.

Figure 2: Spectrum of Oncogenic RET Alterations

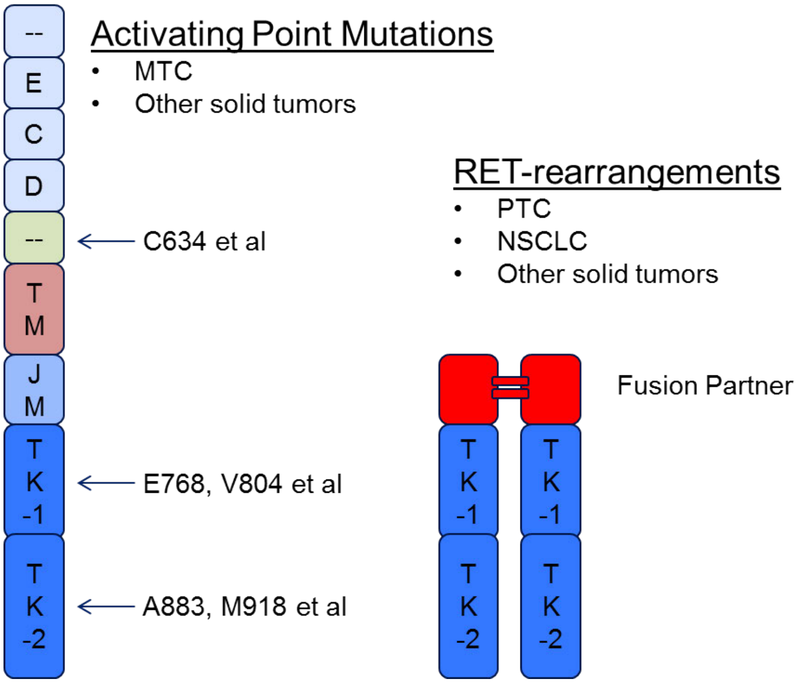

Abbreviations: MTC = medullary thyroid cancer, NCLC = non-small cell lung cancer, PTC = papillary thyroid cancer, RET = rearranged during transfection

Both DTC and MTC are treated with surgery when localized (Fagin and Wells, 2016). Ablative therapy with radioactive iodine (RAI) is effective in DTC patients with recurrence; however, patients eventually become refractory to RAI. As MTC arises from follicular C-cells, RAI is not effective. Once advanced, RAI-refractory DTC and MTC are poorly responsive to chemotherapy and systemic treatment with a small molecule multikinase inhibitor (MKI) is the standard of care (SOC) for both. Sorafenib and lenvatinib are approved MKIs for progressive and/or symptomatic RAI-refractory DTC. Cabozantinib and vandetanib are approved MKIs for advanced MTC. Note that there are no approved diagnostics for assessment of RET status and MKIs are used regardless of RET mutational status. Importantly, MKIs poorly inhibit RET with germline mutations at the 'gatekeeper' residue of the kinase domain (V804L/M) and these substitutions arise when cells are screened for drug resistance mutations to MKIs, such as vandetanib (Carlomagno et al, 2004). Multikinase inhibitors used to treat thyroid cancer have broad activity against many kinases (eg, RAF, MET, EGFR, vascular endothelial growth factor receptor [VEGFR]1-3, PDGFR, RET and others), and are associated with significant dermatologic, cardiovascular, and gastrointestinal side effects. Therefore, National Clinical Practice Guidelines in Oncology (NCCN, 2018) recommends careful monitoring and dose interruption and/or dose modification for drug-related side effects with these agents. For patients with disease progression on MKI therapy or MKI intolerance, there are no effective therapies available globally. However, in May 2020 the selective RET inhibitor selpercatinib received accelerated approval from the US Food and Drug Administration (FDA) for the treatment of adult and pediatric patients ≥12 years of age with advanced or metastatic RET-mutant MTC who require systemic therapy (Selpercatinib, US Prescribing Information).

1.1.2 RET-altered NSCLC

Oncogenic RET rearrangements have been identified in 1-2% of NSCLC (Lin et al, 2015). These rearrangements typically produce chimeric transcripts that encode a fusion protein consisting of the RET kinase domain coupled to a protein with a dimerization domain (eg, KIF5B, CCDC6, NCOA4). This generates a constitutively active kinase that promotes tumorigenesis (Figure 2). As with anaplastic lymphoma kinase (ALK) and ROS1-rearranged NSCLC, RET-rearranged NSCLC is typically adenocarcinoma histology (though occasionally squamous) and occurs in young, non-smoking patients. Because diagnostic testing for RET is not SOC, RET-rearranged patients with advanced NSCLC are treated per NCCN guidelines for EGFR and ALK negative adenocarcinoma. This usually includes first line chemotherapy with a platinum doublet and second line therapy with single-agent cytotoxic chemotherapy or a checkpoint inhibitor; however, clinical response and overall survival (OS) with these agents remains poor. Subsequent therapy for refractory patients per NCCN guidelines is best supportive care (BSC) or clinical trial.

Initial case reports and single-arm studies with the MKIs cabozantinib, vandetanib, sorafenib, and alectinib in patients with known RET-rearranged NSCLC have demonstrated clinical activity, suggesting that RET may be a valid target in NSCLC. Although encouraging response rates (~12-60%) (Gautschi et al, 2017; Horiike et al, 2016; Lin et al, 2016) have been observed in these early studies, duration of response (DOR) is typically less than a year. MKI treatment was associated with significant toxicity, requiring dose interruption and/or dose modification as similarly observed in thyroid cancer, which likely limit exposures required to effectively inhibit RET. Thus, RET-rearranged NSCLC remains an unmet need that requires new therapeutic approaches; particularly those that can selectively and potently inhibit RET in vivo (Drilon et al, 2018).

In May 2020, the selective RET inhibitor selpercatinib received accelerated approval from the US Food and Drug Administration (FDA) for the treatment of adult patients with metastatic RET-fusion positive NSCLC (Selpercatinib, US Prescribing Information).

1.1.3 Other RET-driven Cancers

Emerging genomic and functional data implicate RET as a potential driver in several cancers beyond thyroid cancer and NSCLC (Borrello et al, 2013; Mulligan, 2014). For example, RET rearrangements and potentially activating point mutations have been observed at low frequency in colorectal cancer, breast cancer, and other solid tumors, as well as in chronic myelomonocytic leukemia (Ballerini et al, 2012; Medico et al, 2015; Stransky et al, 2014). Additionally, upregulation of RET/GDNF/GFRαs has also been detected across solid tumor indications and this may promote RET activation, tumorigenesis and drug resistance (Nakashima et al, 2007; Platt et al, 2015; Yang and Horten, 2014). Thus, it is possible that other advanced solid tumors are dependent on RET activity and could respond to RET inhibition.

To date, clinical activity attributable to RET inhibition has been uncertain for these approved MKIs, likely due to insufficient inhibition of RET and off-target toxicities. There is a need for precision therapies that provide durable clinical benefit by selectively targeting RET alterations and anticipated resistance mutations.

1.2 Pralsetinib (previously known as BLU-667)

1.2.1 Preclinical Pharmacology

Pralsetinib (also known as BLU-667, BLU123244, or X581238) is a potent and selective inhibitor of RET and oncogenic RET mutants. In cellular systems, pralsetinib inhibits the kinase activity of RET oncogenic mutants and RET fusion proteins with low nanomolar potency. In vivo, dose dependent antitumor efficacy with pralsetinib was demonstrated in several RET-driven models including a RET C634W mutant-driven MTC xenograft, a CCDC6-RET fusion expressing colorectal cancer patient –derived xenograft (PDX) and a KIF5B-RET-fusion driven NSCLC PDX. Antitumor efficacy was correlated with pralsetinib exposures and pharmacodynamic modulation of tumor biomarkers, including direct inhibition of RET activity. Repeat dose administration to mice at doses ranging from 10 mg/kg twice daily (BID) to 30 mg/kg BID and 60 mg/kg once daily (QD) were well tolerated and resulted in complete tumor growth inhibition or tumor regression. At doses required for complete tumor growth inhibition in these models, a 90% decrease in RET kinase activity was observed.

Table 1: Biochemical Activity of Cabozantinib, Vandetanib, Regorafenib, Ponatinib, and Pralsetinib against Wild Type RET, RET Mutants, and RET Fusion Proteins

	Mean IC_{50} (nM)					
	**RET**	**RET V804L**	**RET V804M**	**RET M918T**	**CCDC6-RET**	**VEGFR-2**
Pralsetinib	0.43	0.33	0.38	0.40	0.45	35
Cabozantinib	11	45	162	8	34	2
Vandetanib	4	3597	726	7	20	4
Regorafenib	12	53	70	25	15	14
Ponatinib	0.6	4	2	0.8	0.8	2

Abbreviations: CCDC6 = coiled-coil domain-containing 6; IC_{50} = half-maximal inhibitory concentration of enzyme activity; RET = rearranged during transfection; VEGFR = vascular endothelial growth factor.
Source: BPM-0015

Figure 3: Efficacy and Body Weight Measurements with Pralsetinib (BLU-667) in a KIF5B-RET NSCLC Patient-Derived Xenograft Model

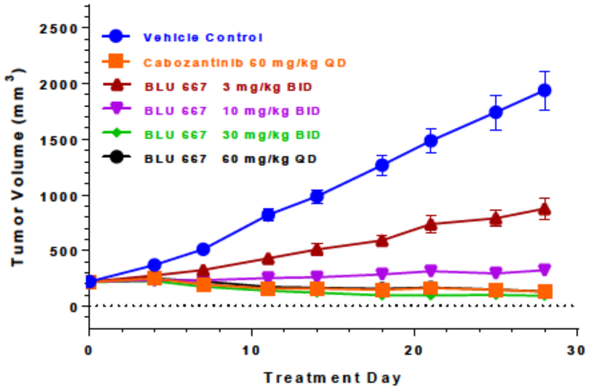

Abbreviations: QD, once daily; BID, twice daily; PDX, patient-derived xenograft
Mice bearing KIF5B-RET fusion lung adenocarcinoma PDXs were dosed orally, once or twice daily as indicated with 3, 10, 30 mg/kg BID or 60 mg/kg QD BLU-667 (pralsetinib) for 28 days. Tumor size was measured twice weekly for the 28 day dosing period. BLU-667 antitumor activity was highly statistically significant ($P \leq 0.01$ for 3 mg/kg BID BLU-667, $P \leq 0.001$ for 10 and 30 mg/kg BID, and 60 mg/kg QD dosing by one way analysis of variance followed by Dunnett's multiple comparison test). Data points represent group mean and error bars represent standard error of the mean for 9 mice per cohort.
Source: 1110-003

The selectivity of pralsetinib on RET versus other human targets was characterized. pralsetinib had a high degree of selectivity for RET over 450 kinases, including > 25-fold selectivity for RET over VEGFR2 and FGFR1, 2 kinases inhibited by several MKIs. pralsetinib also demonstrated high selectivity when tested in a panel of 55 transmembrane or soluble receptors, ion channels and monoamine transporters.

Safety pharmacology studies revealed that pralsetinib inhibits the human ether-à-go-go-related gene (hERG) channel activity in vitro with an average IC_{50} of 5.18 µM, suggesting low potential for prolonging the QT interval. In the cardiovascular safety pharmacology study in radiotelemetry-implanted rats, doses of 150 mg/m^2 (25 mg/kg) and higher were associated with reduced heart rate and increased systolic and diastolic blood pressure, all without clinical signs. However, no electrocardiogram (ECG) changes were noted in toxicology studies.

Additional information can be found in the Investigator's Brochure (IB) for pralsetinib.

1.2.2 Toxicology

Sprague Dawley rats and Cynomolgus monkeys were chosen as the nonclinical test species for assessing chemical structure- and pharmacology-mediated toxicity of pralsetinib on the basis of the following criteria: a) these species have historically been used to assess potential human adverse effects, and; b) these species are qualitatively similar in hepatocyte metabolism profiles to humans, and: c) these species contain identical or highly homologous amino acids surrounding the pralsetinib binding site when

compared against human RET, and: d) these species are pharmacologically responsive to the effects of pralsetinib.

In the 28-day Good Laboratory Practice (GLP)-compliant repeated dose toxicology study in Sprague Dawley rats the toxicities defining the severely toxic dose in 10% of animals (STD_{10}; 180 mg/m^2/day) were inanition and metabolic abnormalities which included reduced food consumption, reduced weight, dermal atonia, and chemistry alterations including increased blood phosphorus.

Adverse, non-dose-limiting toxicity (DLT) and non-severely toxic effects were considered secondary to off-target pharmacology. These effects were characterized by thickened physeal cartilage (femur), hyperphosphatemia with corresponding mineralization in the glandular stomach, incisor tooth degeneration, and decreased bone marrow cellularity with corresponding decrease in red blood cell counts. These effects were either resolving or resolved after the 14-day recovery period with the exception of incisor tooth degeneration. The primary vascular effects on the physeal cartilage in the femur and on the teeth are attributed to impaired angiogenesis secondary to VEGFR inhibition (Chen and Cleck, 2009; Fletcher et al, 2010; Patyna et al, 2008). The primary tissue mineralization effects are attributed to the pharmacologic effect of inhibiting FGFR signaling, which has been observed with other compounds in development (Brown et al, 2005; Yanochko et al, 2013). The primary cellular effects on the bone marrow and are attributed to Janus kinase 2 (JAK2) inhibition (Broxmeyer, 2013; Parganas et al, 1998; Quelle et al, 1994; Springuel et al, 2015).

In the 28-day GLP-compliant repeated dose toxicology study in Cynomolgus monkeys, the lethal dose (480 mg/m^2/day) was associated with erosion and ulceration of the gastrointestinal mucosa, and sepsis. The DLT defining the highest non-severely toxic dose (HNSTD; (90 mg/m^2/day) were inanition, reduced food consumption, reduced body weight, and alterations in chemistry and hematology values.

Other effects in monkeys included decreased bone marrow cellularity, physeal dysplasia, and decreased cellularity of lymphoid organs with decreased lymphocytes. All effects in the HNSTD group were resolving or resolved after the 14-day recovery period. No functional effects on the cardiovascular system were noted based on ECG analyses and in-life examinations. The gastrointestinal ulcerations and sepsis in monkeys are attributed to the pharmacologic effect of inhibiting VEGFR. Reduced bone marrow cellularity was attributed to the pharmacologic effect of inhibiting JAK2. Decreased cellularity of lymphoid organs corresponding with decreased lymphocytes with increased neutrophils and monocytes, were collectively attributed to stress.

Pralsetinib was negative for mutagenic activity in the GLP-compliant 5 strain Ames test, with and without metabolic activation. Pralsetinib was classified as non-phototoxic following in vitro phototoxicity evaluation in 3T3 fibroblasts using the neutral red uptake assay.

Results from the repeated dose GLP-compliant 28-day toxicology studies in rats and monkeys, the non-GLP cardiovascular safety pharmacology studies in radiotelemetry-

implanted rats, and other toxicology studies all inform the clinical program of potential risks to humans in the first-in-human (FIH) study. Overall, results from these studies indicate an acceptable benefit-risk profile for the clinical testing of pralsetinib.

Two 13-week GLP-compliant repeated dose toxicology studies were conducted in Sprague Dawley rats and Cynomolgus monkeys. In the 13-week rat study, adverse dose effects at 20 mg/kg/day (120 mg/m^2/day) in both males and females included tooth degeneration (dentin matrix alteration, ameloblast degeneration, odontoblast degeneration, and odontoblast necrosis), subsequent tooth fractures, decreased hematopoiesis in the bone marrow in the sternum and femur, and decreased lymphoid cellularity in the thymus that corresponded with lower mean thymus weights and gross observation of small thymus. Additional adverse effects at 20 mg/kg/day included tubular degeneration/atrophy in the testis with secondary cellular debris and reduced sperm in the lumen of the epididymis, which corresponded with lower mean testis and epididymis weights, respectively, and gross observations of soft and small testis, and degeneration of the corpus luteum in the ovary at 20 mg/kg/day. The NOAEL was 10 mg/kg/day. This dosage level corresponded to mean area under the concentration-time curve to the last measurable concentration ($AUC_{[0-t]}$) values of 33,300 and 42,300 ng·h/mL and mean C_{max} values of 2360 and 3580 ng/mL for males and females, respectively, on Day 91.

In the 13-week monkey study, pralsetinib was well-tolerated at all dosage levels (ie, 2, 5, and 10 mg/kg/day). Therefore, the NOAEL was considered to be 10 mg/kg/day. This dosage corresponded to mean $AUC_{(0-t)}$ values of 43,200 and 31,900 ng•hr/mL and mean C_{max} values of 2790 and 2850 ng/mL for males and females, respectively, on Day 91.

A GLP-compliant repeat-dose embryofetal toxicology study was conducted to characterize the toxicity of pralsetinib in pregnant female Sprague Dawley rats when dosed at 5, 10, 20, and 30 mg/kg/day (30, 60, 120, and 180 mg/m^2/day) via oral gavage once daily during Gestation Days 6–17. All females in the 20 and 30 mg/kg/day groups had 100% post implantation losses (all early resorptions). A higher mean litter proportion of post implantation loss and lower mean litter proportion of viable fetuses were noted in the 10 mg/kg/day group. Multiple visceral malformations and developmental variations (primarily of the kidney and ureter) and skeletal malformations (vertebral, rib, costal cartilage, and vertebral centra anomalies) and developmental variations (reduced ossification of ribs) were noted at 5 and/or 10 mg/kg/day. Based on the adverse effects on intrauterine survival and/or fetal morphology at all dosage levels, a NOAEL for embryo/fetal development could not be determined.

Additional information can be found in the IB for pralsetinib.

1.3 Study Rationale and Design

Pralsetinib potently and selectively targets RET fusions and RET activated mutants such as M918T and V804L/M, shows potent antitumor activity in RET-driven tumor models, and demonstrates tolerability at pharmacologically active doses in preclinical toxicology species. Together, these data provide strong rationale for exploring the clinical utility of pralsetinib in advanced cancer patients with RET alterations. The study employed a dose

escalation/maximum tolerated dose (MTD) expansion design to define the pralsetinib MTD/recommended Phase 2 dose (RP2D) and to assess the safety, tolerability, PK, pharmacodynamics, and preliminary antineoplastic activity of pralsetinib in RET-altered solid tumors. As pralsetinib demonstrates potent activity against RET fusions, activating mutations and resistance mutations, the overall response rate (ORR) for all RET-altered tumors will be assessed.

The dose escalation part of the trial (Phase 1) has been completed. It included patients with advanced non-resectable NSCLC, advanced non-resectable thyroid cancer and other advanced non-resectable solid tumors. The dose-escalation part employed the Bayesian optimal interval (BOIN) design to define the MTD of pralsetinib. The BOIN design is simple to implement, similar to the traditional 3+3 design, but is more flexible and possesses superior operating characteristics that are comparable to those of the more complex model-based designs, such as the continual reassessment method (CRM). The target toxicity rate for the MTD is 30%. At dose levels above 120 mg the patient must have had a diagnosis of MTC, or the tumor must carry a RET alteration, as per local assessment of tumor tissue and/or blood.

The MTD and RP2D were determined to be 400 mg QD in Phase 1, and enrollment to the dose expansion part of the trial (Phase 2) is ongoing. The dose expansion part will permit a more complete assessment of safety, pharmacokinetics (PK), pharmacodynamics, and antitumor activity in patients with different types of RET-driven malignancies treated with pralsetinib at the RP2D. During the expansion part patients will be enrolled into 9 groups:

- NSCLC patients with a RET fusion who have previously been treated with a platinum-based chemotherapy (Group 1).
- NSCLC patients with a RET fusion not previously treated with a platinum-based chemotherapy, including those who have not had any systemic treatment. Prior platinum chemotherapy in the neoadjuvant and adjuvant setting is permitted if the last dose of platinum was 4 months or more before the first dose of study drug (Group 2).
- MTC patients who have been previously treated with cabozantinib and/or vandetanib (Group 3).
- MTC patients not previously treated with cabozantinib or vandetanib (Group 4).
- RET-fusion solid tumors previously treated with SOC, or that have not adequately responded to standard therapy, or the patient must be intolerant to, or the Investigator has determined that treatment with, standard therapy is not appropriate, or there must be no accepted standard therapy for their disease and the patient is not eligible for any other group. As the intent of this cohort is to enroll a variety of RET-fusion tumor types, Blueprint will notify sites if/when sufficient data are available and accrual should cease for a particular tumor type (Group 5).

- RET-mutated or fusion solid tumors previously treated with a selective RET tyrosine kinase inhibitor, an example of a previous selective RET inhibitor is selpercatinib (TKI; Group 6).
- RET-mutated solid tumors treated with SOC appropriate for the tumor type (Group 7).
- NSCLC patients with a RET fusion who have previously been treated with a platinum-based chemotherapy extension cohort (Group 8, China only).
- MTC patients who were not previously treated with systemic therapy (except prior cytotoxic chemotherapy is allowed) for advanced or metastatic disease extension cohort (Group 9, China only).

1.4 Rationale for the Starting Dose and Dosing Regimen

Selection of the safe starting dose in clinical trials for anticancer small molecules is based on either one-tenth the allometrically scaled STD_{10} in the rat, the most sensitive species based on administered dose, or one-sixth the allometrically scaled HNSTD in monkeys if monkeys cannot tolerate one-tenth of the allometrically scaled rat STD_{10} value. The STD_{10} in the rat is 30 mg/kg/day or 180 mg/m^2/day, one-tenth of which equals 18 mg/m^2/day. The HNSTD in the monkey is 7.5 mg/kg/day or 90 mg/m^2/day, indicating that the monkey can tolerate one-tenth of the rat STD_{10}. Therefore, the safe starting dose for the proposed Phase 1 trial will be 18 mg/m^2 or a flat dose of 30 mg/day based on a 1.7 m^2 human.

The maximum plasma concentration (C_{max}) and area under the plasma concentration versus time curve from 0 to 24 hours (AUC_{0-24}) values at the proposed FIH starting dose of 30 mg QD are projected to be 270 ng/mL and 2300 ng*h/mL, respectively. The starting dose provides an acceptable margin of safety as the predicted C_{max} and AUC_{0-24} values are well below the values observed at the STD_{10} in the rat (C_{max} = 8150 ng/mL and AUC_{0-24} = 113000 ng/mL) and the HNSTD in the monkey (C_{max} = 3255 ng/mL and AUC_{0-24} = 27100 ng*h/mL).

Dosing commenced with QD administration, as in vivo efficacy studies in animal tumor models showed no advantage of BID over QD dosing of pralsetinib. However, based on the predicted pralsetinib half-life of approximately 4-5 hours in humans, BID dosing may be superior. Therefore, starting with the fourth dose level, or at a later dose level, a BID dosing schedule could be explored, as described in Section 6.3, if supported by PK and safety data from the prior dose levels.

1.5 BLU-667-1101 Study Update

Phase 1, Dose escalation was completed on 03Apr18. Phase 2, Dose expansion is currently enrolling patients.

1.5.1 Phase 1 Dose Escalation Results

1.5.1.1 Once Daily Schedule

The first patient, first visit of BLU-667-1101 occurred on 17 March 2017. A total of 53 patients (51 with RET-altered tumors) were enrolled into the QD dose escalation of pralsetinib at doses ranging from 30 mg to 600 mg as outlined in Table 2. Four patients were treated at 600 mg QD and 2 of the 4 patients experienced DLTs (Grade 3 hyponatremia and Grade 3 hypertension). While both DLTs were reversible, per the protocol guidelines, 600 mg QD exceeded the MTD and the MTD for pralsetinib was determined to be 400 mg QD.

Table 2: Pralsetinib Dose Levels and Number of Patients Enrolled

Cohort - Dose	# Patients Treated (N=53)	# Evaluable (N=51[a])	Dose-Limiting Toxicity (DLT)
1 - 30 mg QD	2	1	0
2 - 60 mg QD	6	6	0
3 - 100 mg QD	5	5	1 ALT increased
4 - 200 mg QD	5	5	0
5 - 300 mg QD	5	5	1 tumor lysis syndrome; 1 hypertension
6 - 200 mg QD	8	7	0
7 - 300 mg QD	6	6	0
8 – 400 mg QD	12	12	1 myositis[b]; 1 hypertension
9 – 600 mg QD	4	4	1 hyponatremia; 1 hypertension

Abbreviations: ALT = alanine aminotransferase; DLT = dose-limiting toxicity; QD = once daily.
[a] 2 patients were not evaluable for DLT (at least 75% of Cycle 1 doses not completed and did not experience a DLT)
[b] This event was formerly reported by the site as asthenia.

1.5.1.2 Twice Daily Schedule

A BID dosing schedule was also explored in Phase 1. The BID dosing schedule started at a 300 mg total daily dose (200 mg in the morning, 100 mg in the evening). A total of 9 patients were enrolled into the BID dose escalation: 4 at 300 mg total daily dose (200 mg in the morning, 100 mg in the evening) and 5 at 200 mg total daily dose (100 mg BID). Of the first 4 patients enrolled at the 300 mg total daily dose, 2 experienced DLTs of Grade 3 hypertension and the dose was subsequently de-escalated to 100 mg BID. Two of 5 patients at 100 mg BID experienced DLTs, including 1 patient with Grade 3 hypertension and 1 patient with tumor lysis syndrome. All patients resumed treatment after resolution of DLT to Grade 1 or baseline and continued on study. Based on overall safety, exposure, tolerability, QD was the preferred dosing schedule and chosen for the dose expansion, Phase 2. An MTD for BID dosing was not defined and further exploration is not planned.

1.5.1.3 Rationale for the RP2D

Two of the 4 patients treated with pralsetinib at 600 mg QD experienced DLTs (Grade 3 hyponatremia and Grade 3 hypertension). While both DLTs were reversible, per the protocol guidelines, 600 mg QD exceeded the MTD, and the MTD of pralsetinib was determined to be 400 mg QD. The overall safety across doses 60-400 mg QD was favorable. Most AEs were Grade 1; the most commonly reported AEs in Phase 1 of the study were: constipation (24%), ALT increased (22%), AST increased (20%), and hypertension (16%). Pralsetinib demonstrated rapid absorption, a long $t_{1/2}$, and a dose dependent increase in exposure and pathway inhibition. Exposure at the 400 mg MTD provided sustained coverage above the predicted tumor and brain RET IC_{90}. Based on this, 400 mg was selected as the recommended dose for expansion, which in now enrolling.

1.5.1.4 Pharmacokinetics

In Phase 1 (dose escalation), the mean pralsetinib concentration-time profiles following single oral dose administration, showed rapid absorption into the systemic circulation (median T_{max} of 2 to 3 h) followed by an apparent monophasic elimination. Results showed a comparable profile to that observed in healthy volunteer studies. Apparent oral clearance was comparable between C1D1 (mean of 6.4 to 30.9 L/h) and C1D15 (mean of 8.6 to 40.2 L/h), indicating no apparent time-dependent changes in the PK of pralsetinib. The mean t½ of pralsetinib on C1D1 ranged from 11.3 to 22.9 h (for the 200 to 400 mg dose range), supporting QD dosing. Additionally, the mean t½ was comparable to the values reported in Phase 1 healthy volunteer studies (BLU-667-0101; mean: 13.2 h and BLU-667-0102; mean: 16.9 h).Following repeated QD administration, steady-state was expected to be reached by D4 with < 3-fold accumulation (GeoMean accumulation ratio of 1.65 [CV 96.8%, 400 mg] and 2.75 [CV 70.9%, 400 mg] for C_{max} and AUC, respectively). The steady-state (C1D15) mean plasma pralsetinib concentration-time profiles for each dose level are shown in Figure 4. Furthermore, pralsetinib doses of ≥400 mg on C1D15 resulted in sustained plasma concentrations above both plasma and brain RET IC90 values in ~ 30% of all patients.

Figure 4: Mean (+ SD) Plasma Concentration Profiles of Pralsetinib following Pralsetinib on C1D1 (Single Dose) and at C1D15 (Steady State) Following Once Daily Oral Administration (Dose Escalation)

(A) C1D1; 0–24 hours

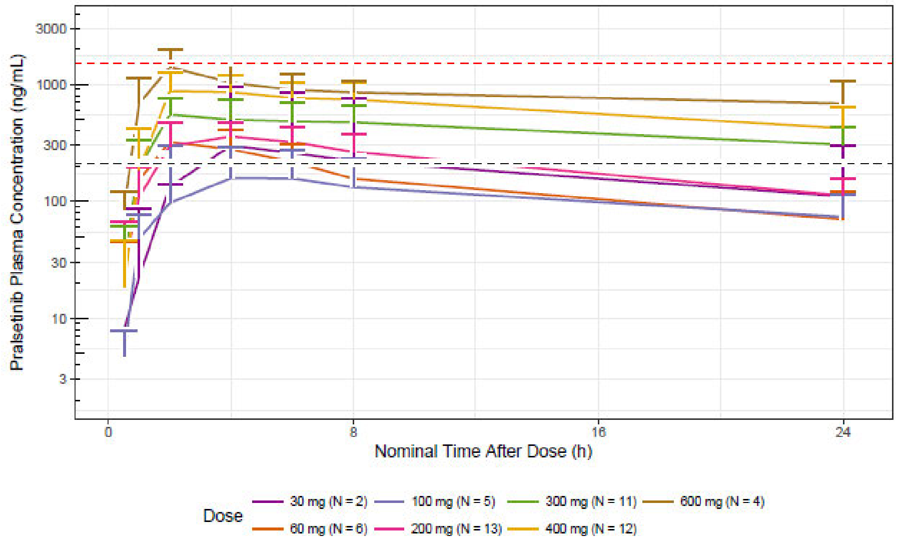

(B) C1D15; 0–24 hours

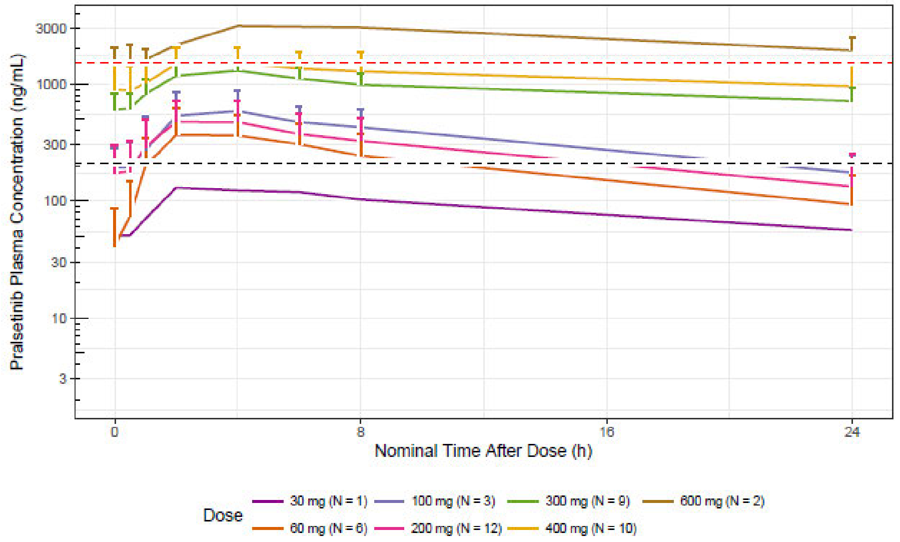

BLQ (< 2 ng/mL) data are excluded. Lines are coloured by dose level. Y-axis is on a log scale. N = number of subjects in each dose cohort.

Abbreviations: C=cycle; D=day; h=hour; IC_{90}=90% inhibitory concentration; PK=pharmacokinetic; RET=rearranged during transfection; SD = standard deviation.
RET IC_{90} measured in mouse tumor xenografts model

1.5.1.5 Pharmacodynamics

RET pathway inhibition was assessed by measuring expression of RET pathway genes DUSP6 and SPRY4 in pre- and post-treatment tumor tissue in a subset of Phase 1 patients. Pralsetinib treatment led to downregulation of DUSP6 and SPRY4 mRNAs in tumor tissue indicating RET pathway inhibition. Increased pathway inhibition was observed with increasing pralsetinib dose.

1.5.2 Phase 2 Dose Expansion Preliminary Data

Phase 2 is actively enrolling at the RP2D.

1.5.2.1 Efficacy

Pralsetinib has demonstrated broad and durable antitumor activity in patients with RET fusion-positive NSCLC and RET-altered MTC and PTC, regardless of treatment history, RET-fusion partner or mutation, or brain involvement.

1.5.2.1.1 Non-small Cell Lung Cancer

Efficacy data are reported as of the data cutoff of 18 November 2019 (Blueprint Medicines Corporation Press Release, 2020) for NSCLC patients treated with pralsetinib who were evaluable for response assessment per RECIST 1.1, as determined by a blinded independent central review. All patients received the proposed indicated dose of 400 mg QD in Study BLU-667-1101. In 80 patients with RET fusion-positive NSCLC previously treated with platinum-based chemotherapy, the ORR was 61% (95% CI: 50- 72%) (2 responses pending confirmation as of the data cutoff date) (Figure 5). Overall, 95% had tumor shrinkage, including 14% of patients with complete regression of target tumors. The median DOR was not reached (95% CI: 11.3 months, not estimable). In 26 patients with metastatic RET fusion-positive NSCLC that were systemic anticancer treatment-naïve, the ORR was 73% (95% CI: 52-88%) (all responses confirmed as of the data cutoff date), with 12% of patients achieving a complete response. All treatment-naïve patients had tumor shrinkage.

Figure 5: Antitumor Activity of Pralsetinib in RET fusion-positive NSCLC

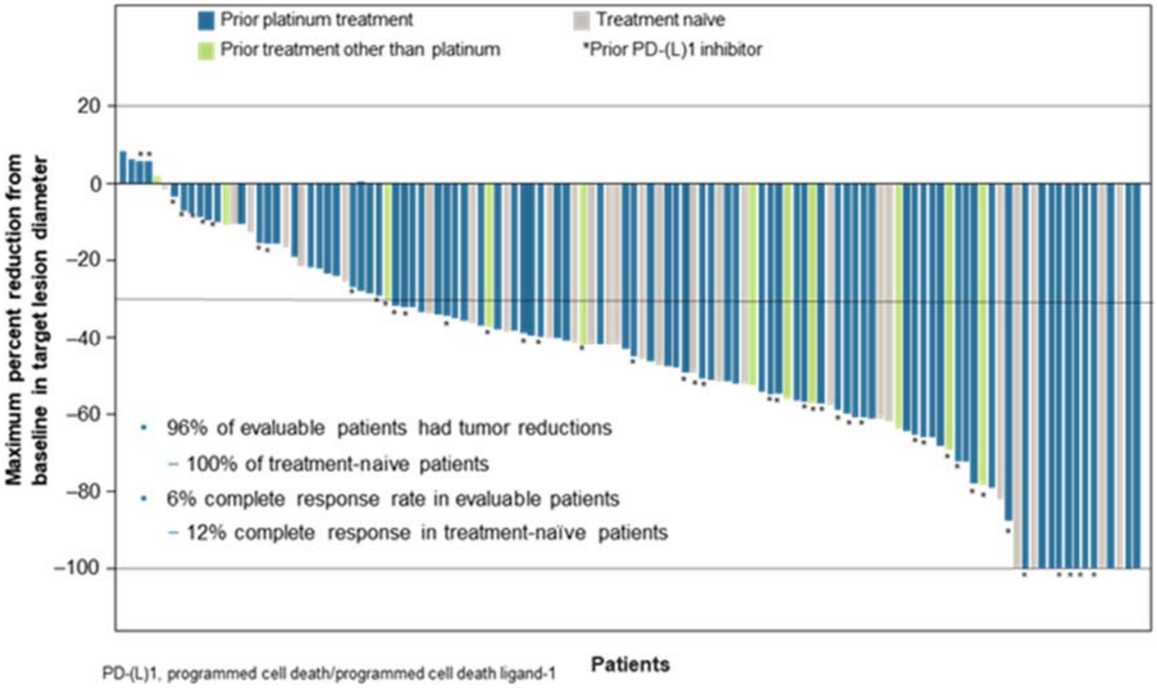

PD-(L)1, programmed cell death/programmed cell death ligand-1

1.5.2.1.2 Thyroid Cancer

Efficacy data are reported as of the data cutoff of 13 Feb 2020 (Blueprint Medicines Corporation Press Release, April 2020). Top-line efficacy data were reported for patients with thyroid cancer treated with pralsetinib who were evaluable for response assessment per RECIST v1.1, as determined by blinded independent central review. All patients received the proposed indicated dose of 400 mg once daily (QD).

In 53 patients with RET-mutant MTC previously treated with cabozantinib or vandetanib, the ORR was 60% (95% CI: 46- 74%) with one response pending confirmation. Nearly all patients (98%) had tumor shrinkage. The median DOR was not reached (95% CI: not estimable, not estimable), and the 18-month DOR rate was 90% (95% CI: 77-100%).

In addition, the top-line data showed robust clinical activity in treatment-naïve patients, supporting the potential of pralsetinib across lines of therapy. In 19 patients with RET-mutant MTC who had not received prior systemic treatment, the confirmed ORR was 74% (95% CI: 49-91%), and all patients had tumor shrinkage. The median DOR was not reached (95% CI: 7 months, not estimable), with 12 of 14 responders remaining in response for up to 15 months as of the data cutoff date.

In nine patients with RET fusion-positive thyroid cancer, the confirmed ORR was 89% (95% CI: 52-100%), and all patients had tumor shrinkage. The median DOR was not reached (95% CI: 8 months, not estimable), with seven of eight responders remaining in response for up to 20 months as of the data cutoff date.

Figure 6: Anti-tumor Activity of Pralsetinib in MTC Patients Irrespective of Prior Therapy

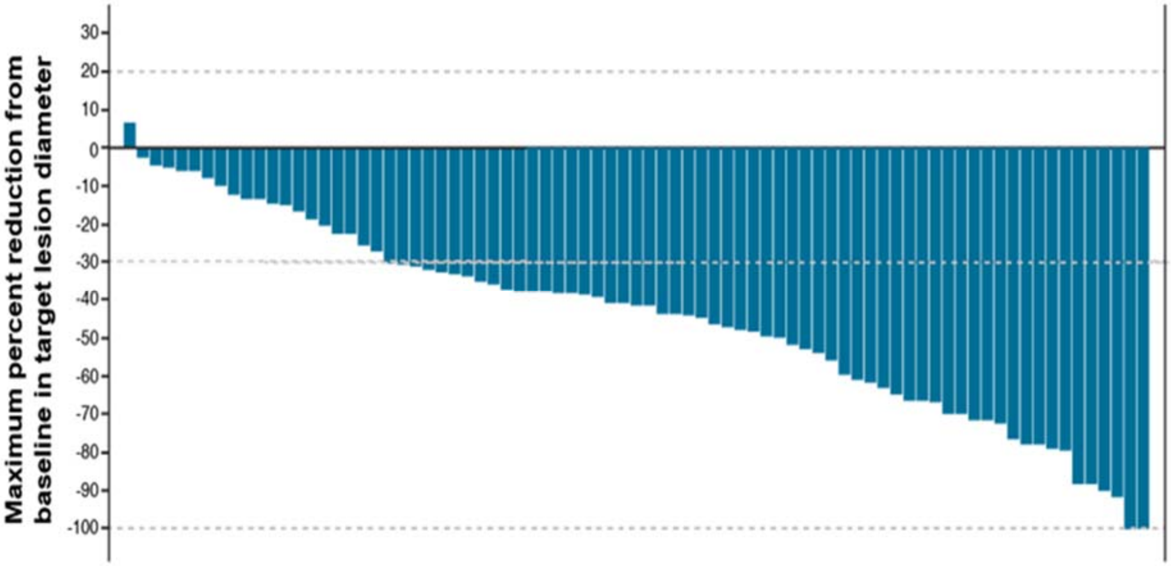

Abbreviations: MTC = medullary thyroid cancer.

Additionally, pralsetinib has FDA breakthrough therapy designation in RET fusion+ NSCLC that progressed following platinum-based chemotherapy and RET-mutated MTC requiring systemic treatment and for which there are no acceptable alternative treatments.

1.5.3 Overall Safety

Overall, pralsetinib QD dosing schedule at dose levels ≤400 mg has demonstrated acceptable tolerability with most AEs being low-grade and reversible. All AEs that occurred in ≥15% of patients, as of the data cutoff date of 18 December 2019, including relationship, are listed in Table 3, and serious AEs considered related to Pralsetinib and occurring in > 1 patient are reported in Table 4.

At the time of the 18 December 2019 data cutoff, 272 of 421 patients (64.6%) remained on treatment. Overall, 149 (35.4%) had discontinued treatment with pralsetinib, including 82 patients who discontinued due to disease progression, 48 patients who discontinued due to AEs (includes 20 patients due to related AEs), 14 patients who discontinued due to withdrawal of consent, 3 patients who discontinued due to Investigator's decision , and 2 patients who discontinued due administrative or other reasons.

Table 3: AEs Occurring in ≥15% of Patients Regardless of Relationship in Study BLU-667-1101 (N=421)

Preferred Term	Any Events n (%)	Severity n (%) Grade ≥3	Related Any Grade n (%)
Aspartate aminotransferase increased	168 (39.9)	22 (5.2)	134 (31.8)
Anaemia	140 (33.3)	53 (12.6)	87 (20.7)
Constipation	140 (33.3)	3 (0.7)	81 (19.2)
Alanine aminotransferase increased	120 (28.5)	14 (3.3)	93 (22.1)
Hypertension	118 (28.0)	58 (13.8)	88 (20.9)
Diarrhoea	116 (27.6)	16 (3.8)	59 (14.0)
Fatigue	93 (22.1)	12 (2.9)	47 (11.2)
Blood creatinine increased	85 (20.2)	1 (0.2)	50 (11.9)
Cough	82 (19.5)	3 (0.7)	10 (2.4)
Pyrexia	82 (19.5)	4 (1.0)	16 (3.8)
Neutropenia	81 (19.2)	44 (10.5)	74 (17.6)
White blood cell count decreased	79 (18.8)	17 (4.0)	68 (16.2)
Dyspnoea	63 (15.0)	11 (2.6)	11 (2.6)

Abbreviation: AE = adverse event.
Source: IB Table 17
Data cutoff date: 18 December 2019.

Table 4: Serious Adverse Events Considered Related to Study Drug Occurring in >1 Patient (N=421)

Preferred Term	SAE n (%)	Severity n (%) Grade ≥3
Pneumonitis	15 (3.6)	10 (2.4)
Pneumonia	8 (1.9)	6 (1.4)
Anaemia	4 (1.0)	3 (0.7)
Hypertension	4 (1.0)	4 (1.0)
Neutropenia	4 (1.0)	3 (0.7)
Blood creatine phosphokinase increased	3 (0.7)	3 (0.7)
Pneumocystis jirovecii pneumonia	3 (0.7)	3 (0.7)
Sepsis	3 (0.7)	3 (0.7)
Thrombocytopenia	3 (0.7)	3 (0.7)
Constipation	2 (0.5)	2 (0.5)
Febrile neutropenia	2 (0.5)	2 (0.5)

Hyponatraemia	2 (0.5)	2 (0.5)
Stomatitis	2 (0.5)	1 (0.2)
Tumour lysis syndrome	2 (0.5)	2 (0.5)
Urinary tract infection	2 (0.5)	1 (0.2)

Abbreviations: SAE = serious adverse event.
Source: IB Table 19
Data cutoff date: 18 December 2019

1.5.4 Overall Benefit-Risk Assessment

Conclusions regarding the benefit/risk of pralsetinib should be taken in the context of its stage of development. Thus far, pralsetinib has demonstrated broad and durable antitumor activity in patients with RET fusion-positive NSCLC and RET-mutated MTC, regardless of treatment history, RET fusion partner or mutation, or CNS involvement. The ORRs in these unmet need populations ranged from 58% up to 83%. The overall safety experience suggests that pralsetinib has a favorable tolerability profile in advanced cancer patients and that most AEs/SAEs are readily detected and managed with current safety monitoring procedures. Taken together, available information indicates that the benefit/risk ratio for pralsetinib therapy is acceptable for further development.

2 STUDY OBJECTIVES

2.1 Phase 1 Objectives

2.1.1 Primary Objectives

1. To determine the MTD and RP2D of pralsetinib.

2. To determine the safety and tolerability of pralsetinib.

2.1.2 Secondary Objectives

1. To determine the ORR by RECIST v1.1 (or Response Assessment in Neuro-Oncology [RANO], if appropriate for tumor type) for all patients treated with BLU-667 according to their disease type, and/or RET-altered status if applicable, and/or prior treatment status, if appropriate. Patients initially treated with MTD/RP2D will be pooled with Phase 2 patients.

2. To assess Baseline RET gene status in plasma and/or tumor tissue and correlate with measures of antineoplastic activity including, but not limited to ORR, clinical benefit rate (CBR), disease control rate (DCR), and DOR.

3. To characterize the PK profile of pralsetinib and correlate drug exposure with safety assessments.

4. To characterize the pharmacodynamics of pralsetinib, including, but not limited to, changes in blood calcitonin and carcinoembryonic antigen (CEA) in MTC patients

only, and changes in tumor dual specificity phosphatase (DUSP6) and sprout RTK signaling antagonist 4 (SPRY4) levels in all patients.

2.1.3 Exploratory Objectives

1. To identify potential new blood and tumor tissue biomarkers (eg, DNA, ribonucleic acid [RNA], and/or protein markers) of pharmacodynamic activity, antineoplastic activity, and/or toxicity.

2.2 Phase 2 Objectives

Phase 1 patients treated at the MTD/RP2D will be pooled with Phase 2 patients for Phase 2 objectives.

2.2.1 Primary Objectives

1. To determine the ORR by RECIST v1.1 (or RANO, if appropriate for tumor type) by disease type, and/or RET-altered status (including patients treated at MTD/RP2D in Phase 1), and/or prior treatment status specified in enrollment group definition, if appropriate.

2. To further define the safety and tolerability of pralsetinib.

2.2.2 Secondary Objectives

1. To assess additional measures of clinical benefit including DOR, CBR, DCR, PFS, and OS in all patients by disease type and/or RET-altered status, and/or prior treatment status explained in enrollment group definition, if appropriate. Patients treated at MTD/RP2D in Phase 1 will be pooled with Phase 2 patients for this analysis.

2. To assess baseline RET gene status (ie, gene fusion partner or primary mutation and, for MTC, whether hereditary or sporadic) in plasma and/or tumor tissue and correlate with measures of antineoplastic activity including, but not limited to ORR, CBR, DOR, and DCR in all patients, including patients treated at MTD/RP2D in Phase 1, by disease type, and/or RET-altered status if applicable, and/or prior specified treatment status if appropriate.

3. To characterize the pharmacokinetic (PK) profile of pralsetinib and correlate drug exposure with safety assessments, including changes in ECG intervals, and efficacy.

4. To characterize the pharmacodynamics of pralsetinib, including, but not limited to, changes in blood calcitonin and CEA in MTC patients only.

5. To assess brain activity in patients with NSCLC.

2.2.3 Exploratory Objectives

1. To identify potential new blood and tumor tissue biomarkers (eg, DNA, RNA, and/or protein markers) of pharmacodynamic activity, antineoplastic activity, and/or toxicity.

2. To assess changes in quality of life (QoL) questionnaire.

3. To explore disease-related symptoms, as measured by bowel movement history (MTC patients only).

4. To explore clinical benefit including ORR, CBR, DCR, PFS for patients previously treated with a selective RET tyrosine kinase inhibitor.

5. To assess brain activity in patients with tumor types other than NSCLC.

3 STUDY ENDPOINTS

3.1 Phase 1 Endpoints

3.1.1 Primary Endpoints

1. MTD and RP2D of pralsetinib.

2. Overall safety profile of pralsetinib, as assessed by the type, frequency, severity, timing, and relationship to study drug of any AEs, SAEs, changes in vital signs, ECGs, and safety laboratory tests.

3.1.2 Secondary Endpoints

1. ORR for all pralsetinib treated patients according to patients' disease type and/or RET-altered status if applicable, and/or prior treatment status if appropriate. Patients initially treated with MTD/RP2D in Phase 1 will be pooled with Phase 2 patients accordingly.

2. RET gene status and correlation between RET gene status and ORR, CBR, DOR, and DCR, and other measures of antineoplastic activity (in plasma and/or tissue) in all patients according to their disease type and/or RET-altered status, if applicable, and/or prior treatment status, if appropriate.

3. PK parameters of pralsetinib: Pharmacokinetic parameters of interest will include, as appropriate, maximum plasma drug concentration (C_{max}), time to maximum plasma drug concentration (T_{max}), time of last quantifiable plasma drug concentration (T_{last}), area under the plasma concentration versus time curve from time 0 to 24 hours postdose (AUC_{0-24}), plasma drug concentration at 24 hours postdose (C_{24}); apparent volume of distribution (V_z/F), terminal elimination half-life ($t_{½}$), apparent oral clearance (CL/F), and accumulation ratio (R).

4. Pharmacodynamic parameters of pralsetinib: changes in tumor/blood including, but not limited to, changes in blood calcitonin and CEA (MTC patients); and changes in tumor biomarker levels (DUSP6 and SPRY4) levels (all patients).

3.1.3 Exploratory Endpoints

1. Levels of exploratory blood and tumor markers (DNA, RNA and protein) as compared to antineoplastic activity, and/or toxicity.

3.2 Phase 2 Endpoints

Phase 1 patients treated at MTD/RP2D will be pooled with Phase 2 patients for Phase 2 endpoints accordingly.

3.2.1 Primary Endpoints

1. ORR by patients' disease type, and/or RET-altered status if applicable, and/or prior treatment status if appropriate.

2. Overall safety profile of pralsetinib, as assessed by the type, frequency, severity, timing, and relationship to study drug of any AEs, SAEs, changes in vital signs, ECGs, and safety laboratory tests.

3.2.2 Secondary Endpoints

- DOR, CBR, DCR, PFS, and OS in all patients by disease type and/or RET-altered status, if applicable, and/or prior treatment status, if appropriate.

- RET gene status (ie, gene fusion partner or primary mutation and, for MTC, whether hereditary or sporadic) and correlation between RET gene status and ORR, CBR, DOR, DCR, and other measures of antineoplastic activity (in plasma and/or tissue) in all patients according to their disease type and/or RET-altered status, if applicable, and/or prior treatment status, if appropriate.

- PK parameters of pralsetinib: Pharmacokinetic parameters of interest will include, as appropriate, maximum plasma drug concentration (C_{max}), time to maximum plasma drug concentration (T_{max}), time of last quantifiable plasma drug concentration (T_{last}), area under the plasma concentration versus time curve from time 0 to 24 hours postdose (AUC_{0-24}), plasma drug concentration at 24 hours postdose (C_{24}); apparent volume of distribution (V_z/F), terminal elimination half-life ($t_{½}$), apparent oral clearance (CL/F), and accumulation ratio (R).

- ECG Assessment: In Phase 2, the effects of pralsetinib on ECG parameters will be evaluated for approximately 20 patients using 12-lead ECGs extracted from continuous recordings (12-lead Holter) on C1D1 and C1D15. Individual ECGs will be extracted in replicate from the 12-lead Holter recordings at specified time points and will be evaluated by a central laboratory. QT intervals will be measured from lead II and will be corrected for heart rate (QTc) using Fridericia's correction factors

(QTcF). The primary QTc parameter will be QTcF. Secondary parameters (heart rate, PR, and QRS, and T-wave morphology) will also be evaluated. Potential effects of pralsetinib will be evaluated as change from predose baseline heart rate, PR, QRS, and QTcF by postdose time point. For the purpose of QT assessment, exposure-response analysis will be performed on the relationship between pralsetinib systemic levels and change in QTcF.

- Pharmacodynamic parameters of pralsetinib: changes in tumor/blood including, but not limited to, changes in blood calcitonin and CEA and biochemical response rate (MTC patients only).
- Intracranial response rate among patients with NSCLC who have measurable (target by RECIST v1.1) lesions in the brain, and time to intracranial progression among all patients with NSCLC.

3.2.3 Exploratory Endpoints

1. Changes in patient-reported outcomes as assessed by the European Organization for Research and Treatment of Cancer Core QoL Questionnaire (EORTC QLQ-C30) instruments.
2. Changes in disease-related symptoms as reported by bowel movement history for MTC patients.
3. ORR, CBR, DCR, PFS for patients previously treated with a selective RET tyrosine kinase inhibitor.
4. Intracranial response rate among patients with tumor types other than NSCLC who have measurable (target by RECIST v1.1) lesions in the brain, and time to intracranial progression among all patients with tumor types other than NSCLC.

4 STUDY PLAN

4.1 Study Design

This is a Phase 1/2, open-label, FIH study designed to evaluate the safety, tolerability, PK, pharmacodynamics, and antineoplastic activity of pralsetinib, a potent and selective RET inhibitor, administered orally in patients with medullary thyroid cancer, RET-altered NSCLC, and other RET-altered solid tumors (Figure 7).

The study consists of 2 parts, a dose-escalation part (Phase 1) and an expansion part (Phase 2). Both parts will enroll patients with advanced non-resectable NSCLC, advanced non-resectable thyroid cancer and other advanced non-resectable solid tumors. Prior to Amendment 9, all patients were required to have progressed following standard systemic therapy, have not adequately responded to standard systemic therapy, or be intolerant to or the Investigator has determined that treatment with standard therapy is not appropriate, or there must be no accepted standard therapy for their disease. RET gene status is assessed locally before enrollment and tissue submission.

All study visits are intended to be conducted on an outpatient basis, but may be conducted on an inpatient basis, as needed. After provision of written informed consent (within 8 weeks before study drug administration), patients will be evaluated for study eligibility during the Screening period within 28 days before study drug administration on Cycle 1, Day 1 (C1D1).

On C1D1, eligible patients will present to the study center approximately 2 hours before the first dose of study drug and will remain at the study center for 8 hours for serial PK sampling, pharmacodynamic sample collection, vital signs measurement, ECG monitoring, safety laboratory tests, and safety monitoring. In Phase 2, patients will complete a QoL assessments (EORTC QLQ-C30). Additionally, in Phase 2 only, continuous ECG Holter monitoring will be performed approximately 1 hour before dosing until after collection of the 8-hour PK sample for 20 patients at select study centers. At these study centers, replicate 12-lead ECGs will be extracted from the continuous recording at the same time points as the PK blood draws. Approximately 72 patients (including ~12 patients in groups 8 and 9) in Phase 2 will complete the serial PK sampling during Cycle 1; otherwise, patients enrolled in Phase 2 will only have predose PK samples collected. See Table 10, Table 11, and Table 12 and Section 7 for a detailed description of study assessments.

A treatment cycle is 28 days in duration. Initially, patients returned to the study center on C1D2 for PK sampling (24 hours after the C1D1 dose), C1D8 and C1D22 for safety monitoring, and on C1D15 and C1D16 for serial PK sampling and safety monitoring (including continuous Holter monitoring for 20 patients in Phase 2 at select study centers). Following Protocol Amendment 9, visits on C1D2 and C1D16 are no longer required, except for patients in Groups 8 and 9, who will undergo serial PK sampling in China according to the country-specific Protocol Amendment 10. Patients will also attend study center visits on C2D1 and C2D15, on Day 1 of C3 through C13, C15, C17, C21, and every 4 cycles thereafter for additional safety monitoring; PK and pharmacodynamic sampling; and disease response assessment by computed tomography (CT) or magnetic resonance imaging (MRI). In Phase 2 only, the EORTC QLQ-C30 will be administered on D1 of every cycle through C12. A tumor biopsy will be performed within 2 weeks (±) of C2D1 (Phase 1 only) and upon disease progression (both Parts), if the patient consents and the procedure is deemed to be safe and medically feasible by the Investigator.

All patients will attend an End-of-treatment (EOT) visit approximately 14 days (±7 days) after the last dose of study drug. A Follow-up telephone contact for resolution of any residual AE will be made on Day 30 (+7 days) after the last dose of study drug, or at the time the patient initiates another antineoplastic therapy. All patients will subsequently be contacted every 3-4 months for PFS (until progression or start of new therapy) and every 3 months for OS follow-up until closure of the study by the Sponsor.

In the event that, due to unforeseen circumstances, a patient is unable to travel to the investigative site for a planned study visit, elements of that visit may be performed remotely (e.g. phone/video calls) or at outside facilities with notification and agreement from the Sponsor. Appropriate guidance documents may be put in place to support these unforeseen circumstances. All efforts should be made to ensure the safety and wellbeing

of the patient prior to continuation of study drug and altered study visits (as above) should be appropriately documented in the source.

Figure 7: Study Schematic

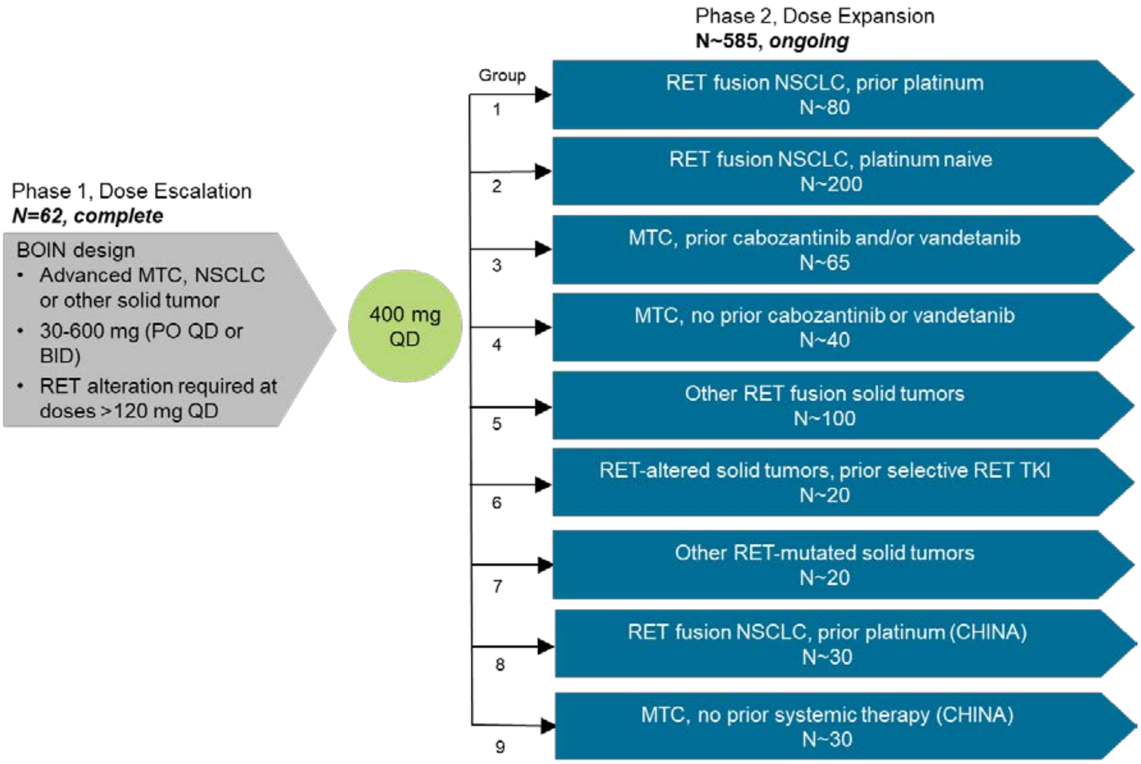

Abbreviations: BOIN = Bayesian optimal interval, BID = twice daily, MTC = medullary thyroid cancer, NSCLC = non-small cell lung cancer, PO = oral, QD = once daily; RET = rearranged during transfection, TKI = tyrosine kinase inhibitor

4.1.1 Phase 1 (Dose-escalation)

The dose escalation (Phase 1) portion of the study completed on 03 April 2018. It enrolled patients with a pathologically documented, definitively diagnosed advanced non-resectable NSCLC, advanced non-resectable thyroid cancer and other advanced non-resectable solid tumors that had progressed following standard systemic therapy, had not adequately responded to standard systemic therapy, or the patient must be intolerant to or the Investigator has determined that treatment with standard therapy is not appropriate, or there must be no accepted standard therapy for their disease. Patients treated at doses > 120 mg per day were required to have MTC or a RET-altered solid tumor per local assessment of tumor tissue and/or blood. Additionally, these patients could be enrolled into an enrichment cohort, if it previously included less than 12 patients evaluable for DLT, was reviewed at a dose-escalation meeting, and did not exceed the MTD. Data from these patients allowed for an improved assessment of safety, PK, and pharmacodynamics.

The dose-escalation part of the study employed the BOIN design (Liu and Yuan, 2015) to find the MTD of pralsetinib. The BOIN design is simple to implement, similar to the traditional 3+3 design, but is more flexible and possesses superior operating characteristics that are comparable to those of the more complex model-based designs, such as the CRM. The target toxicity rate for the MTD is 30%. Dose escalation continued to enroll in cohorts of 3-6 (1-3 for the first 3 dose levels) patients until 12 patients were treated and evaluable for DLT at one dose level. The total number of patients to be enrolled during the dose escalation part of the study could vary depending on the toxicity profile of pralsetinib and the number of dose levels tested prior to reaching the MTD. Dose escalation could also be stopped before reaching the MTD, based on safety findings (such as toxicities beyond C1 that preclude continued dosing), PK data (such as a plateau in drug exposure despite increasing dose), or pharmacodynamic data (such as persistent elimination of circulating tumor DNA). It was originally estimated that dose escalation would require approximately 35 patients.

Pralsetinib was administered QD to patients enrolled at the first 3 dose levels. Starting with the fourth dose level, or at a later dose level, a BID dosing schedule was allowed to be explored, if supported by PK and safety data, as described in Section 6.3. In addition, based on emerging data, other alternative dosing regimens, such as intermittent dosing (eg, 3 weeks on, 1 week off) could be explored. The protocol was to be amended prior to evaluating new dosing schedules that increase dosing frequency, other than BID dosing.

During Phase 1 patient safety was reviewed on an ongoing basis (at least monthly) at a teleconference that includes the study investigators and the Sponsor Clinical Study Team. Available safety data was to be reviewed to confirm that no unexpected, significant, delayed, or unacceptable risks have been discovered. Available PK, pharmacodynamic, and tumor response data was also to be reviewed. Dose escalation decisions, the dose for subsequent dose levels, and MTD/RP2D determination were made at a teleconference, as described above, that was designated in advance to be a dose-escalation teleconference.

Additional details on the dose-escalation process, safety monitoring, estimation of the MTD/RP2D, and DLTs are provided in Section 6.3.1.2 and Section 11.3.

4.1.2 Phase 2 (Expansion)

In Phase 2, patients will enroll into 1 of 7 groups based on their tumor type and prior therapy status (if applicable). Patients will be treated with pralsetinib at the MTD/ RP2D as determined in Phase 1 and must have at least 1 target lesion evaluable by RECIST v1.1 or RANO, with the exception of patients enrolled in Group 7:

- Group 1: NSCLC with a RET fusion previously treated with a platinum-based chemotherapy (N ~80).
- Group 2: NSCLC with a RET fusion not previously treated with a platinum-based chemotherapy, including those who have not had any systemic therapy. Prior platinum chemotherapy in the neoadjuvant and adjuvant setting is permitted if the last dose of platinum was 4 months or more before the first dose of study drug (N~200, including approximately 30 patients with prior systemic therapy).
- Group 3: MTC previously treated with cabozantinib and/or vandetanib (N ~65).
- Group 4: MTC not previously treated with cabozantinib or vandetanib (N ~40).
- Group 5: Other solid tumors with a RET fusion not eligible for any of the other groups (N ~100). Patients should have previously received SOC appropriate for their tumor type, unless there is no accepted standard therapy for the tumor type or the Investigator has determined that treatment with standard therapy is not appropriate. As the intent of this cohort is to enroll a variety of RET-fusion tumor types, Blueprint will notify sites if/when sufficient data are available and accrual should cease for a particular tumor type.
- Group 6: Any solid tumor with a RET alteration (fusion or mutation) previously treated with a selective RET inhibitor (N~20).
- Group 7: Other solid tumors with a RET mutation previously treated with SOC appropriate for the tumor type (N~20).
- Group 8: NSCLC with a RET fusion previously treated with a platinum-based chemotherapy (China only; N ~30)
- Group 9: MTC not previously treated with systemic therapy (except prior cytotoxic chemotherapy is allowed) for advanced or metastatic disease extension cohort (China only; N ~30)

Determination of RET status, as required for enrollment of all patients except those with MTC, is based on local assessment or central assessment; however, patients with MTC are not required to have RET results before enrollment. All patients enrolled in Phase 2

must submit tumor tissue (archived or new) for retrospective assessment of RET status and other pathway biomarkers (see Section 7.5.2). Patients enrolled into Group 6 (previous treatment with a selective RET-inhibitor) are required to have a new tumor biopsy prior to enrollment.

During Phase 2, patient safety will be reviewed on an ongoing basis through various safety teams. Specific details regarding safety monitoring are described in Section 12.2.

5 POPULATION

5.1 Number of Patients

Approximately 647 patients will be enrolled in this study, including:

- A total of 62 patients were enrolled in Phase 1 (Dose Escalation).
- Approximately 585 patients in Phase 2 (Expansion).

The total number of patients enrolled in Phase 1 was dependent upon the observed safety profile, which determined the number of patients per dose cohort, as well as the number of dose escalations required to achieve the MTD.

5.2 Inclusion Criteria

Patients meeting the following criteria will be eligible for participation in the study:

1. Patient is ≥ 18 years of age.
2. Diagnosis during dose escalation (Phase 1) – Pathologically documented, definitively diagnosed non-resectable advanced solid tumor.
    - All patients treated at doses > 120 mg per day must have MTC, or a RET-altered solid tumor per local assessment of tumor tissue and/or blood.
    - Phase 1 enrichment patients must have MTC or a RET-altered solid tumor per local assessment of tumor tissue and/or blood.
3. Diagnosis during dose expansion (Phase 2) – All patients (with the exception of patients with MTC enrolled in Groups 3, 4, and 9) must have an oncogenic RET fusion or mutation (excluding synonymous, frameshift, and nonsense mutations) solid tumor, as determined by local testing of tumor or circulating tumor nucleic acid in blood; as detailed below. For a list of common oncogenic mutation, see Appendix 6.
    - Group 1 – patients must have pathologically documented, definitively diagnosed locally advanced or metastatic NSCLC with a RET fusion previously treated with a platinum-based chemotherapy.

- Group 2 – patients must have pathologically documented, definitively diagnosed locally advanced or metastatic NSCLC with a RET fusion not previously treated with a platinum-based chemotherapy, including those who have not had any systemic therapy. Prior platinum chemotherapy in the neoadjuvant and adjuvant setting is permitted if the last dose of platinum was 4 months or more before the first dose of study drug.

- Group 3 – patients must have pathologically documented, definitively diagnosed advanced MTC that has progressed within 14 months prior to the Screening Visit and was previously treated with cabozantinib and/or vandetanib.

- Group 4 – patients must have pathologically documented, definitively diagnosed advanced MTC that has progressed within 14 months prior to the Screening Visit and was not previously treated with cabozantinib or vandetanib.

- Group 5 – patients must have a pathologically documented, definitively diagnosed advanced solid tumor with an oncogenic RET fusion, have previously received SOC appropriate for their tumor type (unless there is no accepted standard therapy for the tumor type or the Investigator has determined that treatment with standard therapy is not appropriate), and must not eligible for any of the other groups.

- Group 6 – patients must have a pathologically documented, definitively diagnosed advanced solid tumor with an oncogenic RET fusion or mutation, previously treated with a selective TKI that inhibits RET, such as selpercatinib.

- Group 7: patients must have a pathologically documented, definitively diagnosed advanced solid tumor with an oncogenic RET mutation previously treated with SOC appropriate for the tumor type and not eligible for any of the other groups.

- Group 8: patients must have pathologically documented, definitively diagnosed locally advanced or metastatic NSCLC with a RET fusion that was previously treated with a platinum-based chemotherapy (China only).

- Group 9: patients must have pathologically documented, definitively diagnosed advanced MTC that has progressed within 14 months prior to the Screening Visit and was not previously treated with systemic therapy (except prior cytotoxic chemotherapy is allowed) for advanced or metastatic disease (China only).

4. Patients must have non-resectable disease. Prior to Protocol Amendment 9, patients must have progressed following standard therapy or have not adequately responded to standard therapy, or the patient must be intolerant to, or the Investigator has determined that treatment with standard therapy is not appropriate, or there must be no accepted standard therapy for their disease.

5. Dose expansion (Phase 2) patients in all groups (except Group 7) must have measurable disease per RECIST v1.1 (or RANO, if appropriate for tumor type).

6. Patient agrees to provide tumor tissue (archived, if available or a fresh biopsy) for RET status confirmation and is willing to consider an on-treatment tumor biopsy, if considered safe and medically feasible by the treating Investigator. For Phase 2, Group 6, patients are required to undergo a pretreatment biopsy to define baseline RET status in tumor tissue.

7. Patient has Eastern Cooperative Oncology Group (ECOG) performance status (PS) of 0-1.

8. Patient provides informed consent to participate in the study.

5.3 Exclusion Criteria

Patients meeting any of the following criteria will not be eligible for participation in the study:

1. Patient's cancer has a known primary driver alteration other than RET. For example, NSCLC with a targetable mutation in EGFR, ALK, ROS1, or BRAF; colorectal with an oncogenic KRAS, NRAS, or BRAF mutation. Investigators should discuss enrollment with Sponsor regarding co-mutations.

2. Patient has any of the following within 14 days prior to the first dose of study drug:

   a. Platelet count $< 75 \times 10^9$/L.

   b. Absolute neutrophil count (ANC) $< 1.0 \times 10^9$/L.

   c. Hemoglobin < 9.0 g/dL (red blood cell transfusion and erythropoietin may be used to reach at least 9.0 g/dL, but must have been administered at least 2 weeks prior to the first dose of study drug.

   d. Aspartate aminotransferase (AST) or alanine aminotransferase (ALT) > 3 × the upper limit of normal (ULN) if no hepatic metastases are present; > 5 × ULN if hepatic metastases are present.

   e. Total bilirubin > 1.5 × ULN; > 3 × ULN with direct bilirubin > 1.5 × ULN in presence of Gilbert's disease.

   f. Estimated (Cockroft-Gault formula) or measured creatinine clearance < 40 mL/min.

   g. Total serum phosphorous > 5.5 mg/dL.

3. Patient has a QTcF > 470 msec. Patient has a history of prolonged QT syndrome or Torsades de pointes. Patient has a familial history of prolonged QT syndrome.

4. Patient has clinically significant, uncontrolled, cardiovascular disease including congestive heart failure Grade III or IV according to the New York Heart Association

(NYHA) classification; myocardial infarction or unstable angina within the previous 6 months, uncontrolled hypertension, or clinically significant, uncontrolled arrhythmias, including bradyarrhythmias that may cause QT prolongation (eg, Type II second degree heart block or third degree heart block).

5. Patient has central nervous system (CNS) metastases or a primary CNS tumor that is associated with progressive neurological symptoms or requires increasing doses of corticosteroids to control the CNS disease. If a patient requires corticosteroids for management of CNS disease, the dose must have been stable for the 2 weeks preceding C1D1.

6. Presence of clinically symptomatic interstitial lung disease or interstitial pneumonitis, including radiation pneumonitis (ie, affecting activities of daily living or requiring therapeutic intervention).

7. Patient received the following anticancer therapy:

   a. Any systemic anticancer therapy (except for immunotherapy or other antibody therapies) and all forms of radiotherapy, within 14 days or 5 half-lives prior to the first dose of study drug. Pralsetinib may be started within these washout periods if considered by the Investigator to be safe and within the best interest of the patient, with prior Sponsor approval.

   b. Any immunotherapy or other antibody therapy within 28 days prior to the first dose of study drug (immune related toxicities must have resolved to < Grade 2 prior to starting pralsetinib).

8. Dose expansion patients in Groups 1-5 and 7 (Phase 2): patient has previously received treatment with a selective RET inhibitor such as selpercatinib.

9. Patient received neutrophil growth factor support within 14 days of the first dose of study drug.

10. Patient requires treatment with a prohibited medication or herbal remedy (as specified in Appendix 2) that cannot be discontinued at least 2 weeks before the start of study drug administration. pralsetinib may be started within 14 days or 5 half-lives of prior therapy if considered by the Investigator to be safe and within the best interest of the patient, with prior Sponsor approval.

11. Patient has had a major surgical procedure within 14 days of the first dose of study drug (procedures such as central venous catheter placement, tumor needle biopsy, and feeding tube placement are not considered major surgical procedures).

12. Patient has a history of another primary malignancy that has been diagnosed or required therapy (except maintenance anti-hormonal therapy) within the past year. The following prior malignancies are not exclusionary: completely resected basal cell and squamous cell skin cancer, curatively treated localized prostate cancer, curatively

treated localized thyroid cancer, and completely resected carcinoma in situ of any site.

13. Patient is unwilling or unable to comply with scheduled visits, drug administration plan, laboratory tests, or other study procedures and study restrictions.

14. Women who are unwilling, if not postmenopausal or surgically sterile, to abstain from sexual intercourse or employ highly effective contraception during the study drug administration period and for at least 30 days after the last dose of study drug. Men who are unwilling, if not surgically sterile, to abstain from sexual intercourse or employ highly effective contraception during the study drug administration period and for at least 90 days after the last dose of study drug. Refer to Section 9.6.1 for acceptable methods of contraception.

15. Pregnant females, as documented by a serum beta human chorionic gonadotropin (β-hCG) pregnancy test consistent with pregnancy, obtained within 7 days prior to the first dose of study drug. Females with β-hCG values that are within the range for pregnancy but are not pregnant (false-positives) may be enrolled with written consent of the Sponsor, after pregnancy has been ruled out. Females of non-childbearing potential (postmenopausal for more than 1 year; bilateral tubal ligation; bilateral oophorectomy; hysterectomy) do not require a serum β-hCG test.

16. If female, patient is breastfeeding.

17. Patient has prior or ongoing clinically significant illness, medical condition, surgical history, physical finding, or laboratory abnormality that, in the Investigator's or Sponsor's opinion, could affect the safety of the patient; alter the absorption, distribution, metabolism, or excretion of the study drug; or impair the assessment of study results.

5.4 RET Pre-screening

Sites may identify patients with a RET-mutated or RET-fusion solid tumor using local identification methods.

5.5 Patient Identification and Registration

Patients who are candidates for enrollment into the study will be evaluated for eligibility by the Investigator to ensure that the inclusion and exclusion criteria (see Section 5.2 and Section 5.3) have been satisfied.

During both Parts 1 and 2, upon identification of an eligible patient, study centers submitted a request to the Sponsor or designee to register each patient for enrollment. For Phase 1, enrollment was granted based on availability in each escalation cohort.

For Phase 2, enrollment will be closed to a particular group when the target enrollment has been reached or if the Sponsor has decided to close the group once a sufficient number of patients have been enrolled. Patients who have already consented for the study

will be permitted to enroll, within a reasonable period of time, even if the group that they qualify for has been filled.

The Medical Monitor or designee will confirm eligibility for all patients prior to receipt of the first dose of pralsetinib.

Patients who have signed the main ICF and did not meet eligibility criteria should be entered into the clinical database as a screen failure.

5.6 Study Completion

It is anticipated that patients will receive at least 1 cycle of pralsetinib; no maximum treatment duration has been set. After C1, patients may continue to receive pralsetinib until precluded by toxicity, noncompliance, withdrawal of consent, death, or closure of the study by the Sponsor. Patients with progressive disease may remain on treatment if in the opinion of the Investigator the patient has benefited from pralsetinib therapy, and it is clearly in the best medical interest of the patient to remain on treatment.

A patient will be considered to have completed the study if he/she has completed all required visits shown in schedule of assessments in Section 6.6.

5.6.1 Patient Withdrawal Criteria from Study Treatment

Patients have the right to withdraw from the study at any time for any reason.

Patients must discontinue from study treatments for any of the following reasons:

- Withdrawal of consent to continue study treatment
- Pregnancy
- Death

Patients may discontinue from study treatments for any of the following reasons:

- AE
- Disease progression
- Protocol deviation
- Investigator decision

5.6.2 Patient Withdrawal Criteria from the Study

A participant must be withdrawn from the study for any of the following reasons:

- The participant withdraws consent for the study

- Participant death
- Loss to follow-up

When a patient discontinues study drug or withdraws from the PFS/OS follow-up part of the study, the primary reason(s) for discontinuation or withdrawal must be recorded in the appropriate sections of the electronic case report form (eCRF) and all efforts will be made to complete and report final study observations as thoroughly as possible. Withdrawal of consent for continued study treatment should be differentiated from withdrawal of consent for study follow-up, and every effort should be made within the bounds of safety and subject choice to have each subject complete the study follow-up. Patients who discontinue study drug should be seen for an End-of-Treatment Visit, and should undergo additional post-treatment follow-up for safety, disease progression, and survival, as described in Sections 7.8 and 7.9, when feasible.

Following discontinuation of study drug, all efforts will be made to complete and report the protocol-defined study observations as completely as possible and to determine the reason for withdrawal.

All related AEs should be monitored until they are resolved, stabilized, have returned to pre-exposure baseline, determined to be due to another illness, or until a subsequent therapy is initiated. For AEs considered not related to study drug, similar monitoring guidelines will only be required through 30 days after the last dose of study drug. If the patient withdrew from treatment because of an AE, every effort must be made to perform protocol-specified safety follow-up procedures, as outlined in Section 7.8.

If there is a medical reason for withdrawal, the patient will remain under the supervision of the Investigator or designee until the condition has returned to baseline or stabilized.

5.7 Replacement of Patients

Phase 1: To be evaluable for DLT assessment, at least one patient in a cohort must have received at least 75% of their prescribed pralsetinib doses in C1 (ie, ≥ 21 doses) and completed follow-up safety evaluations through C1D28 or experienced a DLT. Patients not evaluable for DLT assessment will be replaced if necessary to ensure that data are available from at least 1 evaluable patient for each cohort prior to the dose-escalation meeting.

6 STUDY CONDUCT

6.1 General Conduct

The study will be conducted at multiple centers in the United States, Asia, and Europe.

This study will be conducted in compliance with the protocol, Good Clinical Practice (GCP), and the applicable regulatory requirements.

The schedule of assessments for the study is provided in Table 10, Table 11, and Table 12.

The minimum duration of patient participation is approximately 3 months, including a screening period to assess study eligibility up to 4 weeks (28 days); a treatment period of at least 1 cycle (28 days); an EOT visit at least 14 ± 7 days following the last dose of study drug; and a Follow-up telephone contact for resolution of any AEs 30 days (+ 7 days) after the last dose of study drug, or at the time the patient initiates another antineoplastic therapy, and PFS and OS follow-up until withdrawal of consent, death, or loss to follow-up.

The end of the study is defined as the time that the last patient completes his/her last visit (LPLV), including assessments performed as part of OS or PFS follow-up. See Section 7 for details of all study assessments.

The expected enrollment period is approximately 60 months and the expected duration of the study from first the subject screened to data base lock is approximately 84 months.

6.2 Early Termination

The study may be terminated early at the discretion of the Sponsor, if there is sufficiently reasonable cause. In the event of such action, written notification documenting the reason for study termination will be provided to each Investigator.

Circumstances that may warrant early termination include, but are not limited to:

- Determination of unexpected, significant, or unacceptable risk to patients (see details in Section 6.2.1).
- Insufficient adherence to protocol requirements.
- Plans to modify, suspend, or discontinue the development of study drug.
- Other administrative reasons.

Should the study be terminated prematurely, all study materials must be returned to the Sponsor or Sponsor's designee.

6.2.1 Rules for Early Termination of Enrollment

In order to further protect the safety of patients, the study incorporates an enrollment stopping rule that terminates further enrollment to the study if there is an excess of permanent treatment discontinuations due to study drug-related AEs.

After 10 patients have received at least 1 dose of pralsetinib, the rate of permanent treatment discontinuations due to drug-related AEs will be assessed at each dose-escalation teleconference during Phase 1 of the study, and at least every 3 months during Phase 2 of the study.

Adverse events meeting all of the following criteria will be included in the assessment:

- The AE is considered drug-related, and is the reason for permanent discontinuation of treatment;
- The AE is Grade ≥ 3, or meets the criteria for DLT (including events that meet the criteria for DLT but occur after C1 or during any cycle in Phase 2);
- The AE occurred after the first dose of study drug, but prior to initiation of a subsequent anticancer therapy;
- The AE occurred while the patient was being treated at a dose that does not exceed the MTD.

Further enrollment to this study will be terminated if the lower bound of the 1-sided 70% exact binomial confidence interval (CI) of the discontinuation rate due to AE is > 15%.

Appendix 7 presents the operating characteristics for the early study termination assessment.

6.3 Dose and Administration

Patients will be dispensed the appropriate number of Sponsor-packaged, labeled bottles on D1 of each cycle to allow dosing for 28 days; alternatively, patients may be dispensed the appropriate bottle(s) until the next scheduled visit. Patients must return all unused study drug (or the empty bottles) at each scheduled visit.

Pralsetinib doses should be administered with a glass of water in a fasted state, with no food intake from 2 hours before until 1 hour after study drug administration. Each dose should be administered at approximately the same time each day. Patients following the QD schedule should take pralsetinib in the morning. Patients following the BID schedule should take one dose in the morning and one dose in the evening, with approximately 10-14 hours between doses. Doses should always be separated by at least 8 hours. Patients should be instructed to swallow study drug whole and to not chew the study drug.

If a patient on the QD schedule forgets to take a dose in the morning, he/she should take pralsetinib until 4 pm that day. If the dose has not been taken by 4 pm, then that dose should be omitted and the patient should resume treatment with the next scheduled dose. If a patient on the BID schedule forgets to take a morning dose, he/she should take pralsetinib until 2 pm that day and at least 8 hours before the next dose, and if he/she forgets to take an evening dose he/she should take pralsetinib until 12 am (midnight) that day, and at least 8 hours before the next dose. Pralsetinib dose should always be administered with a glass of water in a fasted state, with no food intake from 2 hours before until 1 hour after study drug administration. If the dose has not been taken by the specified time, that dose should be omitted and the patient should resume treatment with the next scheduled dose.

If a patient vomits during or after taking pralsetinib, re-dosing is not permitted until the next scheduled dose.

6.3.1 Dosing for Phase 1 (Dose Escalation)

Pralsetinib was administered QD to patients enrolled at the first 3 dose levels. Starting with the fourth dose level, or at a later dose level, a BID dosing schedule could be explored, if supported by PK and safety data. The highest permitted BID dose for the first cohort treated on a BID schedule was 50% of the QD dose at the last highest dose level deemed safe and did not exceed the MTD. Thus, the total daily dose at the first BID dose level did not exceed the total daily dose at the highest, safe QD dose level.

A temporary discontinuation (up to 2 weeks) in pralsetinib dosing is allowed for patients who require an interruption (eg, for surgery or another procedure) during the treatment period. Pralsetinib should be discontinued 48 hours before the procedure and resumed 48 hours after the procedure is completed.

6.3.1.1 Alternate Dosing Schedule

Alternate dosing schedules, such as intermittent dosing (eg, 3 weeks on, 1 week off) were permitted for evaluation if supported by PK, pharmacodynamic, safety, or efficacy data. A decision to change the treatment schedule, including the choice of a starting dose for the new schedule, could only occur after agreement was reached among the study Investigators and the Sponsor Clinical Study Team. A protocol amendment was required prior to evaluating dosing schedules that increase dosing frequency, other than BID dosing, as described above. Enrollment to Phase 1 is complete and alternate schedules (other than BID) were not evaluated.

6.3.1.2 Guidelines for Dose-escalation

Dose-escalation decisions will be made by the study Investigators and Clinical Study Team.

The dose-escalation process is performed following the BOIN design, as follows:

1. Patients in the first cohort are treated at dose level 1.

2. To assign a dose to the next cohort of patients the dose escalation/de-escalation decision is made according to the rules displayed in Table 5. When using Table 5 “Eliminate” means the removal of the current and higher doses from the trial to prevent treating any future patients at these doses because they are overly toxic.

- If a dose is eliminated, automatically de-escalate the dose to the next lower level. Patients being treated at higher doses may continue at the higher dose if they are tolerating it. If the lowest dose is eliminated, the trial will be stopped for safety. In this case, no dose should be selected as the MTD.

- If none of the actions (ie, escalation, de-escalation or elimination) is triggered, new patients will be treated at the current dose.

- If the current dose is the lowest dose and the rule indicates dose de-escalation, new patients will be treated at the lowest dose unless the number of DLTs reaches the elimination boundary, at which point the trial will be terminated for safety.

3. Repeat step 2 and stop the trial when the number of patients treated at the current dose reach 12 (Table 5).

Table 5: Dose Escalation/De-escalation Rule for the BOIN Design

	The number of patients treated at the current dose:											
Action	1	2	3	4	5	6	7	8	9	10	11	12
Escalate if # of DLT ≤	0	0	0	0	1	1	1	1	2	2	2	2
De-escalate if # of DLT ≥	1	1	2	2	2	3	3	3	4	4	4	5
Eliminate if # of DLT ≥	NA	NA	3	3	4	4	5	5	5	6	6	7

Abbreviations: BOIN = Bayesian optimal interval, DLT = dose-limiting toxicity, NA = not applicable.

Figure 8: Dose-Escalation Schematic

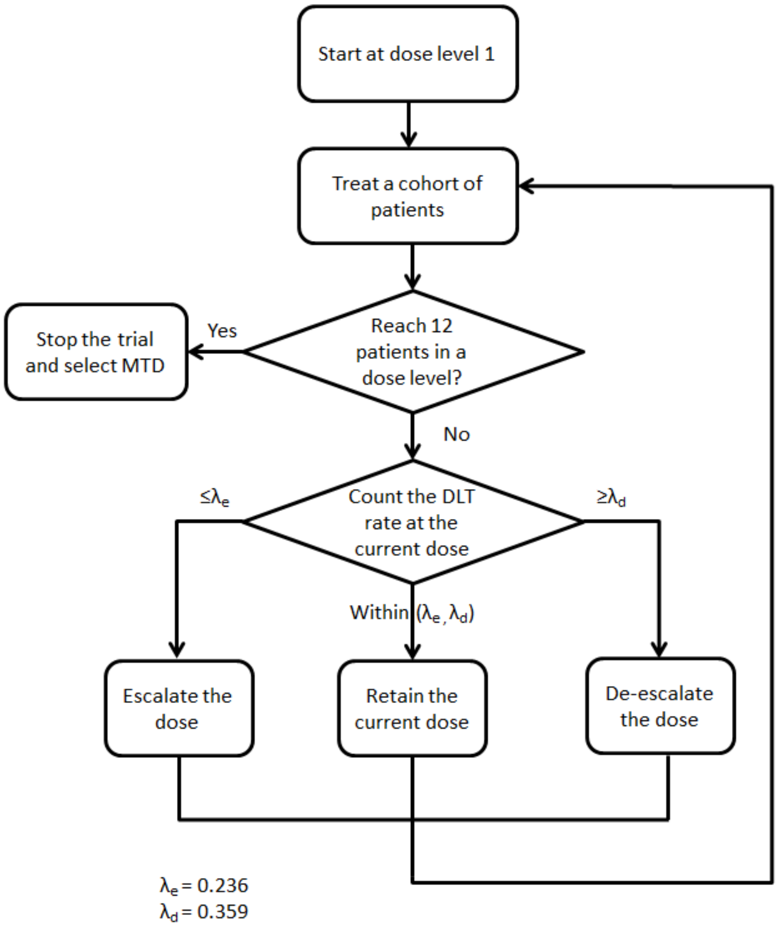

Abbreviations: DLT = dose-limiting toxicity, λ_e = dose escalation boundary, λ_d =dose de-escalation boundary, MTD = maximum tolerated dose.

After Phase 1 of the trial is completed, the MTD is determined based on isotonic regression as specified by Liu and Yuan (Liu and Yuan, 2015). A lower RP2D may also be selected based on clinical data, including safety, PK, pharmacodynamics and antitumor activity. See protocol Section 11.3 for details.

To limit the number of patients treated at a subtherapeutic dose; the study uses a cohort size of 1-3 patients for the first 3 dose levels, and then transitions to a cohort size of 3-6 patients for all subsequent dose levels.

The first cohort of patients will receive pralsetinib at a starting dose of 30 mg QD. The increment in dose between cohorts may be a maximum of 100% until 1 patient in any dose level experiences a DLT, or any 2 patients in any dose level experience a Grade ≥ 2 AE considered related to pralsetinib during C1. All subsequent dose escalation increments will be a maximum of approximately 50%. The dose may be rounded down or up to accommodate available dosage strengths (Table 6). Actual dose levels explored in Phase 1 are outlined in Table 2.

Three to 6 patients will be enrolled initially in each cohort (1 to 3 patients for the first 3 dose levels). Additional patients may be enrolled into an enrichment cohort at a lower dose, if it previously included less than 12 patients evaluable for DLT, was reviewed at a dose-escalation meeting, and does not exceed the MTD. The total number of patients evaluable for DLT for any given dose level should not exceed 12. Any DLT observed in enrichment patients contributes to the dose escalation/de-escalation decision following the same rules as outlined in Table 5. Data from these patients will allow for an improved assessment of safety, PK, and pharmacodynamics. See Figure 8 for the Dose-escalation Schematic.

A dose escalation meeting will be held by teleconference prior to opening each subsequent dose cohort. The dose escalation meeting will include the study investigators and the Sponsor Clinical Study Team. The meeting will occur after all patients in the current cohort have completed at least 28 days of observation after their first dose of pralsetinib or experienced a DLT. Patients enrolled into enrichment cohorts at lower dose levels need not have completed 28 days of observation for a dose-escalation meeting to occur. All available safety data from all patients, across all cycles of therapy, and including all prior dose cohorts will be reviewed to confirm that no unexpected, significant, delayed, or unacceptable AEs have been discovered that may affect the decision to escalate the dose, or the specific dose that should be chosen. Available PK, pharmacodynamic, and tumor response data will also be reviewed in order to support the dose escalation decision. The decision to open the subsequent cohort, the specific dose that is chosen, and MTD/RP2D determination must be agreed to by the study investigator (or their physician designee) at each study center and the Sponsor Clinical Study Team.

Table 6: Provisional Dose-escalation Levels

Dose Level Cohort	Dose (mg)	Schedule	Total Daily Dose (mg)	%Change from Previous Dose Level
-1	20	QD	20	-29%
1 (Starting Dose Level)	30	QD	30	-
2	60	QD	60	100%
3	120	QD	120	100%
4[a]	60	BID	120	0% (change to BID)
5	120	BID	240	100%
6	240	BID	480	100%
7[b]	360	BID	720	50%
8	500	BID	1000	39%[c]
9	750[d]	BID	1500	50%

Abbreviations: BID = twice daily, QD = once daily

[a] Dose schedule may change from QD to BID at dose level 4, as shown here or at a higher dose level, or dosing may remain QD.

[b] Dose interval is shown changing from 100% to 50% at dose level 7; however, this change is dependent on the observed safety profile, and may occur earlier, or potentially later during dose escalation.

[c] An escalation less than 50% is shown as an example, when it may make the dose choice more convenient.

[d] Further dose escalation will be up to approximately 1.5 times the previous dose, rounded up or down to accommodate available dosage strength.

Note: intermediate and/or additional dose levels may be considered based on the observed safety profile of pralsetinib.

6.3.1.3 Intra-patient Dose-escalation

To minimize the number of patients treated at potentially inactive doses, intra-patient dose escalation is permitted after a patient has completed ≥ 2 cycles of treatment without experiencing Grade 3/4 AE related to study drug or a DLT. With approval from the Sponsor, a patient may be allowed to dose escalate after a Grade 3/4 related AE or a DLT if the AE has resolved to ≤ Grade 1 or the patient's baseline, and the Investigator believes it is in the patient's best medical interest to resume pralsetinib at a higher dose. The patient's dose may then be escalated to a dose that has been reviewed at a dose-escalation meeting and does not exceed the MTD. Additionally, patients enrolled to a BID schedule may transition to the QD MTD. Any intra-patient dose-escalation requires written approval by the Sponsor and should be done at the start of the next cycle.

6.3.1.4 Dose-limiting Toxicity

DLT is defined as any AE of Grade ≥ 3 occurring during C1 during Phase 1 (dose escalation) that is not clearly caused by something other than pralsetinib with the following exceptions:

- Thrombocytopenia is a DLT only if it is Grade 4.
- Neutropenia is a DLT only if it is Grade 4.
- Lymphopenia is a DLT only if it is Grade 4.
- Anemia is a DLT only if it is Grade 4.
- Abnormalities in serum calcium, magnesium, phosphorus and glucose are DLTs only if they are Grade 4; or if they are Grade 3 and the patient is clinically symptomatic, requires hospitalization, or if the specific abnormality does not return to baseline or Grade ≤ 2 within 7 days.
- Nausea is a DLT if it is Grade 3 and persists > 3 days despite antiemetic treatment.
- Vomiting is a DLT if it is Grade 4, or if it is Grade 3 and persists > 3 days despite antiemetic treatment.
- Diarrhea is a DLT if it is Grade 4 or if it is Grade 3 and persists > 3 days despite antidiarrheal treatment.
- Alkaline phosphatase increase is a DLT only if it is Grade 4.
- Total bilirubin Grade 2 is a DLT if associated with ALT Grade ≥ 2.
- Infection or fever in the absence of neutropenia are DLTs if they are Grade 4 or if they are Grade 3 and persist > 7 days.
- Rash is a DLT if it is Grade 4 or if it is Grade 3 and persists > 7 days.
- Fatigue is a DLT only if it is Grade 3 and persists > 7 days.
- Other clinically important AEs that do not meet the specified DLT criteria may be considered to be a DLT upon review at a Dose-escalation meeting.

6.3.1.5 Dose Reduction for Adverse Events

Patients who experience a DLT will have pralsetinib interrupted and be followed until the DLT resolves to Grade ≤ 1 or until the patient's baseline value is achieved, if higher.

If the DLT was thrombocytopenia or neutropenia, blood counts must have recovered to the following: platelet count ≥ 75 × 10^9/L, and ANC ≥ 1 × 10^9/L, respectively.

After resolution of the AE, with a maximum of 2 weeks' dose interruption, the patient may resume therapy with a reduction of 1 dose level. If the Investigator considers it to be in the patient's best interest, treatment may resume without dose reduction, with the written approval of the Sponsor. If the AE recurs with the same or worse intensity, dosing must be interrupted as described above, and the dose must be reduced if the patient resumes therapy. A maximum of 3 dose reductions are permitted for any patient.

6.3.1.6 *Maximum Tolerated Dose*

The MTD was determined to be 400 mg QD based on isotonic regression as specified by Liu and Yuan (Liu and Yuan, 2015). Specifically, the MTD is the dose for which the isotonic estimate of the toxicity rate is closest to the target toxicity rate (30%). If there are ties, the MTD is the higher dose level when the isotonic estimate is lower than the target toxicity rate, and the MTD is the lower dose level when the isotonic estimate is greater than the target toxicity rate.

6.3.2 *Dosing for Phase 2 (Dose Expansion)*

6.3.2.1 *Recommended Phase 2 Dose*

The MTD and RP2D was determined to be 400 mg QD.

All patients enrolled in Phase 2 (Dose Expansion) will begin treatment with pralsetinib at 400 mg QD.

6.3.2.2 *Dose Modification Recommendations for Adverse Events*

Dose modification guidelines for pralsetinib are summarized in Table 7. These guidelines should be followed by clinical Investigators; however, for an individual patient, dose interruptions, reductions, and treatment discontinuation should also be based on the clinical circumstance. Deviation from these guidelines must be documented and communicated with the Sponsor.

Adverse events are to be graded according to NCI CTCAE version 4.03. Guidance for re-escalation after resolution of adverse drug reactions is provided in Section 6.3.2.4.

Pralsetinib dose reductions to below 100 mg QD are not permitted (see Table 7). If a patient requires dose reduction below these dose levels, study treatment should be discontinued.

Doses may be interrupted for study-drug related toxicities for up to 28 days (4 weeks). In general, if a study drug-related toxicity does not resolve to ≤ Grade 2 or has not returned to baseline after dose interruption for more than 28 days, the patient should be discontinued from study treatment unless after discussion with the medical monitor, resumption of treatment is considered to be in the best medical interest of the patient, and this is documented in writing. Additionally, the Sponsor's medical monitor must be contacted if an AE deemed unrelated to treatment requires a dose interruption for more 28 days; a longer recovery period is permitted with written approval by the Sponsor. If a

patient resumes treatment after a dose hold >28 days, an unscheduled disease assessment should occur prior to restarting pralsetinib, to assess status of disease after the dose hold.

For patients who require an interruption due to surgery or another major procedure, pralsetinib should be discontinued 48 hours before the procedure and resumed 48 hours after the procedure is completed.

Table 7: Dose Modification Guidelines for Pralsetinib-Related Toxicity

Toxicity	Modification
Non-Hematologic Toxicity (except for selected AEs - see specific advice below for pneumonitis, hyperphosphatemia, and hypertension)	
Grade 1	• No dose interruption or modification required
Grade 2	• No dose interruption or modification required, if dose interruption is necessary, on improvement resume dosing without dose reduction
Grade 3	• Occurrence: Hold until event is ≤ Grade 2, or has returned to baseline, and then resume by reducing the dose by 100 mg less than the current dose<br>• Occurrence at 100 mg: Discontinue pralsetinib
Grade 4	• Occurrence: Hold until event is ≤ Grade 2, or has returned to baseline, and then resume by reducing the dose by 100 mg less than the current dose<br>• If same AE recurs at Grade 3 or higher, discontinue pralsetinib
Hematologic Toxicity– Anemia, Neutropenia, Thrombocytopenia	
Grade 1 or Grade 2	• No dose interruption or modification required
Grade 3 or 4	• Occurrence: Hold until event is ≤ Grade 2, or has returned to baseline, and then resume by reducing the dose by 100 mg less than the current dose<br>• Occurrence at 100 mg: Discontinue pralsetinib
Hematologic Toxicity – Lymphopenia	
Grade 1 or Grade 2	• No dose interruption or modification required
Grade 3	• Occurrence: Reduce the dose by 100 mg less than the current dose. Interruption of dosing can be done based on clinical circumstances but is not required.<br>• Occurrence at 100 mg: Discontinue pralsetinib
Grade 4	• Occurrence: Hold until event is ≤ Grade 3, or has returned to baseline, and then resume by reducing the dose by 100 mg less than the current dose<br>• Occurrence at 100 mg: Discontinue pralsetinib

6.3.2.3 Dose Modification and Management of Specific Treatment-related Adverse Events

Comprehensive assessments of any study drug-related AEs (adverse drug reactions) experienced by the patient will be performed throughout the course of the study. The severity of the event, as well as clinical judgment will be utilized to determine

appropriate management of the patient for any AE experienced while participating in this study.

Any medication, including those administered for therapy of symptoms considered to be associated with study drug administration, should be reported on the appropriate concomitant medication page of the patient's eCRF. The symptoms should be reported on the AE page.

6.3.2.3.1 Pneumonitis

Pneumonitis, including interstitial lung disease, has been reported with patients treated with pralsetinib, including fatal events. Pneumonitis is a known side effect of TKIs, particularly TKIs used in NSCLC, and has also been observed with pralsetinib. Drug-related pneumonitis may be associated with signs and symptoms such as dyspnea, hypoxia, cough, hemoptysis, and fever as well as radiologic evidence of parenchymal or interstitial changes.

The diagnosis of pneumonitis and determination of causal relationship to the drug is often confounded by the underlying disease (especially lymphangitic carcinomatosis), previous therapies and other factors such as lung infection and radiation effect due to non-specific signs and symptoms as well as similar radiological appearance. Most of the patients with the diagnosis of pneumonitis that was assessed as related to pralsetinib had received prior therapies (before initiation of pralsetinib) that are known to cause pneumonitis (eg, pemetrexed, pembrolizumab, vandetanib, docetaxel, osimertinib). Pneumonitis should be suspected when such signs and symptoms develop or in asymptomatic patients when a new ground glass opacity or interstitial infiltration is noted in imaging studies. If a patient is considered to have the potential diagnosis of drug-related pneumonitis, physical examination, assessment of O_2 saturation, and evaluation for infectious etiologies, and thoracentesis, bronchoscopy, or open lung biopsy should be considered to reach a diagnosis. Dose modifications including dose interruption, potential dose reduction or discontinuation should be considered as noted in Table 8. Based on the clinical picture, if pneumonitis cannot be ruled out, immediate dose interruption and treatment should not be delayed. Treatment of pneumonitis may involve the need for corticosteroids and supportive oxygen and should be based on the clinical judgment and follow standard best practice guidelines.

Table 8: Pralsetinib Dose Modification Recommendations for Treatment-related Pneumonitis

Toxicity Grade	Dose Modification
Grade 1	Interrupt dosing for a minimum of 7 days.<br>After dose interruption if pneumonitis is improved or stable, dosing may be resumed without a dose reduction<br>If pneumonitis recurs, interrupt dosing until it has resolved, then dosing may be resumed with a dose reduction of 100 mg less than the previous dose.

Toxicity Grade	Dose Modification
	If pneumonitis recurs again, consider permanently discontinuing pralsetinib. Further dose interruption and dose reduction is at the discretion of the Investigator and risk should be balanced with the need to treat the underlying disease.
Grade 2	Interrupt dosing for a minimum of 7 days.<br>Resume dosing with a dose reduction of 100 mg when the pneumonitis has improved to Grade 1 or has resolved.<br>If pneumonitis recurs, consider permanently discontinuing pralsetinib. Further dose interruption and dose reduction is at the discretion of the Investigator and the risk should be balanced with the need to treat the underlying disease.
Grade 3	Permanently discontinue treatment, unless the Investigator discusses with the Sponsor and believes the risk-benefit justifies retreatment, and even then, the pneumonitis must be completely resolved, and the dose must be reduced by 100 mg less than the current dose.<br>If restarted on a reduced dose and pneumonitis recurs, then permanently discontinue treatment.
Grade 4	Permanently discontinue treatment.

6.3.2.3.2 Pneumonia/Lung Infections

The diagnosis of pneumonia and lung infections (including opportunistic infections), some of which have been fatal, have been reported with patients treated with pralsetinib. The determination of causal relationship to the drug is often confounded by immune suppression related to the patients underlying disease, prior therapy (such as immunotherapy and steroids) and other risk factors for infection such as myelosuppression. Pneumonias and other lung infections should be managed with systemic antibiotics and other supportive management. If the causality is at least possibly related to the study drug, dose interruption and potential dose reduction and/or discontinuation should follow the guidelines in Table 7, dose modification for non-hematologic toxicity.

6.3.2.3.3 Tumor Lysis Syndrome

Tumor lysis syndrome (TLS) has been observed following pralsetinib treatment. Investigators should monitor electrolyte status and renal function via laboratory testing. Patients with TLS may require supportive care with intravenous fluids and correction of electrolyte imbalances.

Patients that are at risk for TLS at study entry, such as those with high tumor burden, should be well hydrated before initiation of pralsetinib and avoid dehydration during the first cycle. If TLS is suspected, Investigators should manage the adverse event(s) according to standard institutional practices or accepted oncology management guidelines (Coiffier et al, 2008; Klastersky et al, 2016).

6.3.2.3.4 Hyperphosphatemia

As hyperphosphatemia was characterized in the pralsetinib GLP toxicology study in rats (Section 1.2.2), dose modification guidelines have been included in Table 9 for the management of hyperphosphatemia (Dienstmann et al, 2014).

Table 9: Pralsetinib Dose Modification Recommendations for Treatment-related Hyperphosphatemia

Serum Phosphate	Dose Modification
≥ 5.5-7 mg/dL	Continue pralsetinib<br>Dietary phosphate intake restriction<br>Phosphate binder<br>• Sevelamer 1 tablet (800 mg) per meal (or comparable phosphate binder); ie, every 8 hours<br>• Increase dose of Sevelamer up to 1200 mg (or comparable phosphate binder) every 8 hours if needed (phosphate levels still increasing after 1 week)
> 7-9 mg/dL	Continue pralsetinib<br>If serum phosphate levels continue > 7-9 mg/dL despite 2 weeks of phosphorus lowering therapy, the dose of pralsetinib should be reduced. If serum phosphate levels continue > 7-9 mg/dL despite dose reduction and optimal phosphorus lowering therapy for 2 weeks, temporarily discontinue pralsetinib. Restart at reduced dose level when serum phosphate < 7 mg/dL.<br>Dietary phosphate intake restriction<br>Phosphate binder<br>• Sevelamer 2 tablets (1600 mg) per meal (or comparable phosphate binder); ie, every 8 hours<br>• Phosphaturic agents – Acetazolamide 1 tablet (250 mg) 2-3 times per day (or comparable agent)
> 9 mg/dL	Discontinue pralsetinib<br>Restart at reduced dose level when serum phosphate < 7 mg/dL<br>Dietary phosphate intake restriction<br>Phosphate binder<br>• Sevelamer 2 tablets (1600 mg) per meal (or comparable phosphate binder); ie, every 8 hours<br>• Phosphaturic agents – Acetazolamide 1 tablet (250 mg) 2-3 times per day (or comparable agent)
Repeated episodes > 9 mg/dL or simultaneous alteration in renal function	Permanently discontinue pralsetinib

6.3.2.3.5 Hypertension

Treat with standard-of-care antihypertension therapy. Dose modifications not required if hypertension is able to be managed with supportive medications. If dose modification is necessary, please use dose medication for non-hematologic toxicity (Table 7).

6.3.2.4 Dose Re-escalation After Resolution of Adverse Drug Reactions

Re-escalation after dose modification for AEs is discouraged. However, if in the opinion of the treating investigator re-escalation is warranted, this must be undertaken after consultation with the sponsor. To be a candidate for re-escalation, the AE that led to dose modification must not have recurred, and no other AEs of Grade 3 or 4 must have been observed during the preceding 28 days.

If the above criteria are met, the daily dosing of pralsetinib may be re-escalated from the reduced dose level to the immediate previously administered dose level.

Patients may receive step-wise pralsetinib dose re-escalations up to 400 mg QD (eg, 100 mg QD to 200 mg QD to 300 mg QD to 400 mg QD) if the above criteria continue to be met.

A patient should be treated and tolerate therapy well for at least 1 cycle at each higher dose level before the dose is escalated again. In no circumstances should a patient receive a dose higher than 400 mg QD.

6.4 Prior and Concomitant Therapy

All medications administered and procedures conducted within 28 days prior to C1D1 should be recorded on the eCRF. In addition, all prior treatments for the underlying malignancy should be recorded.

6.4.1 Prohibited Concomitant Therapy

The following medications and procedures are prohibited during the study:

- In vitro metabolism studies indicate that pralsetinib oxidation is primarily mediated by CYP3A4 and to a lesser extent by CYP2D6 and CYP2C9, and glucuronidation by UGT1A4. As a precaution, strong inhibitors as well as inducers of CYP3A4 are prohibited. If coadministration of pralsetinib with a strong CYP3A inhibitor cannot be avoided, the dose of pralsetinib should be reduced. Drug transporter studies in cells overexpressing P-glycoprotein (P-gp) indicate that pralsetinib is likely also a P-gp substrate. Medications that are strong dual inhibitors of P-gp and CYP3A4 are prohibited.

  Please refer to Appendix 2 for a list of prohibited medications and foods.

- Any investigational agent or device other than pralsetinib, including commercially available agents that are investigational for the treatment of the patient's underlying malignancy.
- Any antineoplastic treatment other than study drug.
- Neutrophil growth factor support is prohibited within 14 days before C1D1 and throughout C1, unless the patient experiences a DLT of neutropenia.

If radiation therapy or surgical excision of target lesion is determined to be of best clinical interest for the patient and cannot be avoided, the medical monitor should be consulted. Note that radiation or surgical excision of target lesions may/will render patients unevaluable for further response assessments.

6.4.2 *Concomitant Therapy to be used with Caution*

In vitro metabolism studies in human liver microsomes have demonstrated that pralsetinib is a direct moderate inhibitor and inducer of multiple P450 enzymes (CYP2C8, CYP3A4 and CYP2C9). Pralsetinib is also a time-dependent inhibitor of CYP3A4/5. Pralsetinib is an inhibitor of P-gp, BCRP, OATP1B1, OATP1B3, OAT1, MATE1, and MATE2-K at clinically-relevant concentrations. In patients, pralsetinib may alter or increase the plasma concentration of co-administered sensitive CYP2C8, CYP3A4, CYP2C9, P-gp, BCRP, OATP1B1, OATP1B3, OAT1, MATE1, and MATE2-K substrates. Hence, medications that are sensitive CYP2C8, CYP3A4, CYP2C9, P-gp, BCRP, OATP1B1, OATP1B3, OAT1, MATE1, and MATE2-K substrates with known drug-drug interaction potential should be used with caution. Lists of drugs and foods to be used with caution are provided in Appendix 3.

6.4.3 *Permitted Concomitant Therapy*

Medications and treatments other than those specified in Section 6.4.1 and Section 6.4.2, including palliative and supportive care for disease-related symptoms, are permitted during the study.

Antiemetic and antidiarrheal treatments may be used at the Investigator's discretion and in accordance with the local guidelines after documented nausea, vomiting or diarrhea has occurred without medications having been used. Prophylaxis for nausea, vomiting or diarrhea may be instituted beginning on C2D1 in patients who experience these toxicities during C1, and during C1 for patients who have experienced nausea, vomiting or diarrhea.

6.5 Additional precautions

None.

6.6 Schedule of Assessments

Table 10: Schedule of Assessments

**Study Activities[a]**	**Screening**	**Study Treatment**													**OS Follow-up**
**Cycle**		**C1**						**C2**		**C3-C13, C15, C17[b]**	**C21 and every 4 cycles thereafter[b]**	**EOT[c]**	**Follow-up**	**PFS Follow-up[d]**	
**Study Day**	**-28 to -1**	**D1**	**D2[s]**	**D8**	**D15**	**D16[s]**	**D22**	**D1**	**D15**	**D1**	**D1**	**14 days post last dose**	**30 days post last dose**	**Every 3 to 4 months post EOT**	**Every 3 months post EOT**
**Window (days)**				**±1**	**+2**	**+2**	**+2**	**+4**	**±3**	**±4**	**±7**	**±7**	**+7**	**±14**	**±14**
Informed Consent[e]	X														
Inclusion/Exclusion Criteria	X														
Demographics	X														
Medical History[f]	X	X													
Physical Examination[g]	X	X		X	X		X	X	X	X	X	X			
Vital Signs	X	X		X	X		X	X	X	X	X	X			
Serum or Urine Pregnancy Test[h]	X							X		X	X	X			
ECOG PS	X	X						X		X	X	X			
12-lead ECG/ECG Extraction from Holter Monitor[i]	Refer to Table 11 for ECG schedule														
Hematology[j]	X	X		X	X		X	X	X	X	X	X			
Coagulation[j]	X	X						X				X			

**Study Activities[a]**	**Screening**	**Study Treatment**													
**Cycle**		**C1**						**C2**		**C3-C13, C15, C17[b]**	**C21 and every 4 cycles thereafter[b]**	**EOT[c]**	**Follow-up**	**PFS Follow-up[d]**	**OS Follow-up**
**Study Day**	**-28 to -1**	**D1**	**D2[s]**	**D8**	**D15**	**D16[s]**	**D22**	**D1**	**D15**	**D1**	**D1**	**14 days post last dose**	**30 days post last dose**	**Every 3 to 4 months post EOT**	**Every 3 months post EOT**
**Window (days)**				**±1**	**+2**	**+2**	**+2**	**+4**	**±3**	**±4**	**±7**	**±7**	**+7**	**±14**	**±14**
Serum Chemistry[j,k]	X	X		X	X		X	X	X	X	X	X			
Urinalysis[j]	X	X										X			
pralsetinib Administration[l]		X													
PK Blood Sample(s)[m]		X	X		X	X		X		X	X				
Plasma Sample for RET Allele Measurement & Exploratory Markers[n]		X			X			X		X	X	X			
Blood Sample CEA, Calcitonin (Pts with MTC only); Central Lab		X						X		X	X	X			
Blood Sample for TSH and free T4 (Pts with thyroid cancer only); Local Lab		X						X		X	X	X			
Tumor Sample[o]	X					X						X			
Tumor Imaging[p]	X									X	X	X		X	
Telephone Contact													X		X
EORTC QLQ-C30[q]		X						X		X	X				
AE Monitoring[r]		X													

**Study Activities[a]**	**Screening**	**Study Treatment**										**EOT[c]**	**Follow-up**	**PFS Follow-up[d]**	**OS Follow-up**
**Cycle**		**C1**						**C2**		**C3-C13, C15, C17[b]**	**C21 and every 4 cycles thereafter[b]**				
**Study Day**	**-28 to -1**	**D1**	**D2[s]**	**D8**	**D15**	**D16[s]**	**D22**	**D1**	**D15**	**D1**	**D1**	**14 days post last dose**	**30 days post last dose**	**Every 3 to 4 months post EOT**	**Every 3 months post EOT**
**Window (days)**				**±1**	**+2**	**+2**	**+2**	**+4**	**±3**	**±4**	**±7**	**±7**	**+7**	**±14**	**±14**
SAE Monitoring/ Pretreatment Event	X														
Concomitant Medications	X														

Abbreviations: AE = adverse event; C = cycle; CEA = carcinoembryonic antigen; CT = computed tomography; D = day; ECG = electrocardiogram; ECOG PS = Eastern Cooperative Oncology Group Performance Status; EORTC QLQ-C30 = European Organization for Research and Treatment of Cancer Core Quality of Life Questionnaire; EOT = end-of-treatment; MTC = medullary thyroid cancer; MRI = magnetic resonance imaging; PFS = progression-free survival; PK = pharmacokinetic(s); Pts = patients; SAE = serious adverse event; TSH = thyroid-stimulating hormone; T_4.= thyroxine.

Note: Every effort should be made to keep the schedule of assessments on time for each patient.

[a] In C1 and C2, all visits should be performed as noted in Table 10. Laboratory tests, urinalysis, and physical exam performed in screening on D-1 can count as C1D1 laboratory tests and do not need to be repeated on C1D1. After C1, D1 of each subsequent cycle may be delayed up to 28 days to allow for resolution of toxicities. Additional safety tests (eg, hematology, chemistry, ECG) may be performed whenever clinically indicated, at the Investigator's discretion. Unless otherwise indicated, all tests and procedures must be performed predose at each visit. Whenever a test result is questionable, it should be repeated immediately.

[b] Study visits will occur from C3 to C13 at 4-week intervals. After C13, study visits will occur every 2 cycles ±4 days (every 8 weeks: C15 and C17). Following C17, visit will occur every 4 cycles ±7 days (every 16 weeks: C21 ±7 days, C25 ±7 days etc.) to coincide with tumor imaging. Additional unscheduled visits and/or laboratory assessments should be performed as clinically indicated between formal study visits.

[c] If an alternate treatment is started within 14 days of the last dose of study drug, the EOT visit should be conducted prior to the first dose of alternate therapy. EOT procedures do not need to be repeated if they were completed within 7 days (or within 28 days for disease response assessments).

[d] After completion of the EOT visit, all patients without documented progressive disease will be followed up for PFS every 3 to 4 months (±14 days; as per the schedule at the time of treatment discontinuation) until documentation of progressive disease and, subsequently, all patients will be followed for OS every 3 months (±14 days).

[e] Informed consent may be obtained up to 56 days (8 weeks) before C1D1.

[f] A complete medical history will be obtained at the Screening visit. Only disease-related symptoms and changes from the previous visit need to be collected on C1D1.

[g] A complete physical examination will be performed at the Screening visit, including height. Subsequent physical examinations will be disease- and AE-focused.

[h] To be performed for women of childbearing potential within 7 days of C1D1. A serum pregnancy test should be performed at Screening; thereafter, a serum or urine pregnancy test should be performed monthly.

[i] A single 12-lead ECG will be performed at the Screening visit. Extensive ECG sampling via Holter monitoring will be performed at select study centers for 20 patients in Phase 2. See Table 11 for the ECG schedule.

[j] If the Screening visit tests are performed within 7 days of C1D1, clinical laboratory tests do not need to be repeated on C1D1.

[k] On C1D1 serum chemistry testing must be performed under fasting conditions, prior to pralsetinib dosing.

[l] Pralsetinib will be administered QD in the morning or BID in the morning and evening (see Section 6.3) with no food intake from 2 hours before until 1 hour after study drug administration. On study visit days when PK samples are collected, patients will take their morning pralsetinib dose at the study center.

[m] Blood samples for PK assessment will be collected on C1D1, C1D2 (24 hours post C1D1 dose), C1D15, C1D16 (24 hours post C1D15 dose), and predose on D1 of C2 to C4. Refer to Table 11 for PK sampling time points.

[n] Blood samples will be collected for RET allele measurement & exploratory biomarker analyses for first 13 cycles of treatment, as detailed in Table 12 and in the laboratory manual.

[o] Refer to Table 12. Tumor biopsy at Screening, if performed, may be obtained up to 56 days (8 weeks) prior to C1D1

[p] Disease response assessment (per RECIST v1.1 or RANO, if appropriate for tumor type) will be based on local imaging scans, to be performed at Screening, on D1 of every odd numbered cycle from C3 ±4 days to C17 ±4 days, thereafter every 4 cycles ±7 days (16 weeks; ie, C21 ±7 days, C25 ±7 days, etc.) and at EOT. Computed tomography with IV contrast of the chest (CT of the chest is preferred; however, MRI may be substituted), and CT or MRI with IV contrast of the abdomen, brain (MRI is preferred), and pelvis, will be performed. In addition, for patients with thyroid cancer, CT with IV contrast or MRI of the neck will be performed. At subsequent time points, all body regions that contained sites of disease (target or non-target) at screening will be imaged. For each patient, the same method for tumor imaging used at baseline should be used throughout the study. Disease response assessment will also be performed every 3 to 4 months (±14 days; as per the schedule at the time of treatment discontinuation) after EOT in patients without documented progressive disease.

[q] Only during Phase 2, EORTC QLQ-C30 will be administered on D1 of each cycle for C1 through C12.

[r] SAEs that are assessed as possibly or probably related to study treatment that occur > 30 days post-treatment will also be reported.

[s] Cycle 1 Days 2 and 16 are no longer required for patients enrolled under Protocol Amendment 9, except for ~12 patients in Groups 8 and 9 (China only) who will undergo serial PK sampling under Protocol Amendment 10 (China-specific protocol amendment).

Table 11: Schedule for Pharmacokinetic Sample Collection and ECG Monitoring

**Visit/Cycle**	**Screen**	**C1**																**C2-C4**	**EOT**
**Day**	**-28 to -1**	**D1**							**D2[g]**	**D15**							**D16 [g]**	**D1**	
**Time (hours)[a]**		**Pre-dose**	**0.5 ±5 min**	**1 ±5 min**	**2 ±5 min**	**4 ±10 min**	**6 ±10 min**	**8 ± 10 min**	**24 Pre-dose**	**Pre-dose**	**0.5 ±5 min**	**1 ±5 min**	**2 ±5 min**	**4 ±10 min**	**6 ±10 min**	**8 ±10 min**	**24 Pre-dose**	**Pre-dose**	
**Parts 1 and 2[b]**: PK Sampling		X	X	X	X	X	X	X	X[c]	X[c]	X	X	X	X	X	X	X[c]	X[c]	
**Parts 1 and 2**: 12-lead ECG	X	X[d]				X[d]			X[d]	X[d]				X[d]				X	X
**Phase 2 only[e]**: ECG Extraction from Holter Monitor		X[f]	X	X	X	X	X	X	X	X[f]	X	X	X	X	X	X	X		

Abbreviations: C = cycle, D = day, ECG = electrocardiogram, EOT = end-of-treatment, PK = pharmacokinetic(s).

[a] The sampling window is 4 hours for the predose samples, ±5 minutes for the 0.5, 1, and 2-hour time points, and ±10 minutes for the 4, 6, and 8-hour time points. Refer to Table 10 for visit windows allowances.

[b] Approximately 72 patients (including ~12 patients in Groups 8 and 9) enrolled in Phase 2 will undergo serial PK sampling at C1D1 and C1D15. The remaining patients enrolled in Phase 2 will only have predose samples collected.

[c] Record time of day that patient took their pralsetinib dose the prior day.

[d] For all patients in Phase 1 and Phase 2, including those participating in continuous Holter monitoring. Single 12-lead ECGs will be obtained.

[e] For 20 patients in study centers participating in continuous Holter monitoring. Twelve-lead ECGs are to be conducted/extracted after 5 minutes of recumbency or semi-recumbency.

[f] Three time points will be extracted within 1 hour prior to dosing (eg, 45 minutes, 30 minutes, and 15 minutes prior to dosing).

[g] Timepoints not required at Cycle 1 Days 2 and 16 for patients enrolled as of Protocol Amendment 9, except for the patients in Groups 8 and 9 (China only) who will undergo serial PK sampling under Protocol Amendment 10 (China-specific protocol amendment).

Table 12: Schedule for Pharmacodynamic Biomarker Sample Collection

**Cycle**	**Screening**	**C1**		**≥ C2**	**EOT**
**Day**		**D1**	**D15**	**D1**	
**Time (hours)**		**Predose**	**Predose**	**Predose**	
Plasma sample for Exploratory Markers		X		X[a]	X
Plasma sample for RET allele measurement in ctDNA[a]		X	X	X	X
Tumor sample[b,c]	X[e]		X[c]		X[d]

Abbreviations: C = cycle, D = day, EOT = end-of-treatment, ICF = informed consent form, RET = rearranged during transfection, ctDNA = circulating tumor deoxyribonucleic acid.

[a] RET allele measurement in ctDNA and exploratory marker collection will be performed D1 of C1, C2, C3, and every other cycle thereafter (to correspond with RECIST/RANO assessments) through the first 13 cycles of treatment.

[b] Tumor tissue obtained outside the 28-day screening window may be submitted for the Screening time point upon patient consent via tissue screening ICF. Pretreatment tumor tissue (archived or new tissue) will be analyzed centrally for assessment of tumor RET gene status.

[c] In Phase 1 only, tumor biopsy to be obtained on the same day between C2D1 ± 2 weeks if safe and medically feasible, within 2 to 8 hours after pralsetinib administration. A plasma sample for exploratory marker should also be collected as close as possible to the time of the biopsy.

[d] Optional tumor biopsy upon disease progression, if safe and medically feasible.

[e] Screening biopsy is required for all Phase 2, Group 6 patients.

7 DESCRIPTION OF STUDY PROCEDURES

7.1 RET pre-screening

Any patient that will undergo RET testing outside of current SOC should sign the pre-screening ICF prior to acquisition of tumor sample. Pre-screening may occur any time prior to screening. Patients should wait until the RET results are known before signing the main study informed consent form.

7.2 Screening

Following informed consent, all patients will undergo screening procedures within 28 days prior to dosing on C1D1 to determine eligibility. Consent may be obtained up to 56 days (8 weeks) prior to C1D1.

During Screening, either archival or new tumor biopsy samples are to be collected for patients for retrospective determination of the baseline RET gene alteration. Note that a centrally confirmed RET gene alteration is not required for study entry; however, in the event that local RET testing is not available, then enrollment may be based on the central laboratory results.

The following procedures will also be performed at the Screening visit:

- Obtain demographic data, including gender, date of birth/age, race, and ethnicity;
- Complete medical history, including a cancer history, and other malignancies, concurrent illnesses, prior treatments and the response to each treatment if available. For MTC patients, information on bowel movements will also be requested. The medical history obtained at C1D1, however, will be focused on disease-related symptoms and changes from the previous visit;
- Complete physical examination, including ECOG PS;
- Obtain tumor sample (fresh biopsy or archival tumor);
- Tumor imaging (MRI or CT);
- Vital signs including weight, temperature, pulse, and systolic/diastolic blood pressure. Height will also be measured at Screening;
- Single 12-lead ECG;
- Clinical laboratory assessment (hematology, coagulation, serum chemistry, urinalysis;
- Serum pregnancy test within 7 days prior to dosing on C1D1 (women of childbearing potential only);

- Recording of concomitant medications and SAEs. Concomitant medications are recorded from 28 days before start of drug, while SAEs are collected from the time Informed Consent is signed.

Patients that fail screening are allowed to re-screen, if the Investigator feels that it is in the best interest of the patient.

7.3 Safety Assessments

The schedule of safety assessments is described in Table 10. Additional safety assessments may be performed when clinically indicated, at the Investigator's discretion.

7.3.1 Physical Examination

A complete physical examination will be performed at the Screening visit. Subsequent physical examinations will be performed as outlined in Table 10, and will be disease- and AE-focused.

For MTC patients, information on bowel movements will also be requested. Information should only be collected during study at subsequent physical exams if there is bowel movement information available for the patient at baseline.

7.3.2 Eastern Cooperative Oncology Group Performance Status

Determination of ECOG PS will be performed at the time points outlined in Table 10. Please refer to Appendix 1 for ECOG PS scoring.

7.3.3 Vital Signs

Vital sign measurement will include temperature, systolic/diastolic blood pressure, pulse, and weight, and will be performed at the time points outlined in Table 10. Height will be measured at the Screening visit only.

Blood pressure and pulse assessments should be conducted while the patient is seated or supine.

7.3.4 ECGs

Single 12-lead ECG will be obtained for all patients at the time points outlined in Table 10 and Table 11.

In Phase 2 of the study only, continuous ECG Holter monitoring will be performed for 20 patients at select study centers. Replicate 12-lead ECGs will be extracted from the continuous recording at the time points outlined in Table 11.

Twelve-lead ECGs are to be conducted/extracted after 5 minutes of recumbency or semi-recumbency.

Instructions for acquisition of Holter ECG data will be provided in a separate ECG manual.

7.3.5 Clinical Laboratory Tests

Clinical laboratory evaluations for safety will be performed at a local laboratory. Prior to starting the study, the Investigator will provide the Sponsor (or its designee) copies of all laboratory certifications and normal ranges for all laboratory assessments to be performed by that laboratory.

Clinical laboratory evaluations will be conducted at the time points outlined in Table 10. In addition, all clinically significant laboratory abnormalities noted on testing will be followed by repeat testing and further investigated according to the judgment of the Investigator.

The following safety laboratory tests are to be evaluated by the Investigator:

**Hematology:**	Hemoglobin, white blood cell count (WBC) with differential (including ANC), and platelet count
**Coagulation:**	Prothrombin time (PT), international normalized ratio (INR), and activated partial thromboplastin time (aPTT)
**Serum chemistry[a]:**	Sodium, potassium, blood urea nitrogen (BUN) or urea, bicarbonate (venous) or CO_2 level in blood (China only), creatinine, calcium, chloride, magnesium, phosphorus, glucose (screening and C1D1 only), albumin, AST, ALT, alkaline phosphatase (ALP), total bilirubin (direct bilirubin if total > ULN)
**Thyroid tests**	Thyroid-stimulating hormone (TSH) and free thyroxine (T4) (both only in patients with thyroid cancer [MTC and DTC])
**Urinalysis (dipstick):**	pH, specific gravity, bilirubin, blood, glucose, ketones, leukocyte esterase or white blood cells (China only), nitrite, protein, and urobilinogen
**Serum or urine pregnancy[b]:**	β-hCG

[a] On C1D1 patients must have fasted overnight prior to serum chemistry testing, all serum chemistry tests should be performed prior to pralsetinib administration.
[b] A serum pregnancy test should be performed for women of childbearing potential within 7 days prior to C1D1; thereafter, a serum or urine pregnancy test should be performed monthly.

7.3.6 Adverse Events and Concomitant Medications

Each patient must be carefully monitored for the development of any AEs throughout the study from C1D1 (or from the time of signing informed consent, for SAEs) to 30 days

after the last dose. In addition, SAEs that are assessed as related to study treatment that occur > 30 days post-treatment also are to be reported.

Complete details on AE and SAE monitoring are provided in Section 9 and Section 10.

Concomitant medications will be recorded from the time of signing informed consent to 30 days after the last dose.

7.3.7 Thyroid Hormone Replacement in Patients with Thyroid Cancer

Increases in TSH have been reported in studies of multitargeted kinase inhibitors in patients with thyroid cancer (Brose et al, 2014). Therefore, the need to increase thyroid hormone replacement to maintain TSH in the target range will be recorded as an AE.

For patients with MTC, thyroid hormone supplementation should be used as needed to provide physiological replacement, and maintain TSH in the normal range for the institution, typically 0.4-4.0 mIU/L. For patients with DTC, thyroid hormone supplementation should be used as needed to maintain TSH suppression as defined by the institution or Investigator, typically TSH < 0.1 mIU/L.

If an increase in thyroid hormone dose is required to maintain TSH in the target range, this should be recorded as “Grade 1 TSH Increase”.

7.4 Pharmacokinetic Assessment

Serial blood samples will be collected at the time points outlined in Table 11 to determine circulating plasma concentrations of pralsetinib.

When the timing of a blood sample coincides with the timing of a 12-lead ECG measurement or extraction, the ECG will be completed within 1 hour timeframe to the collection of the blood sample. When the timing of a blood sample coincides with the timing of an ECG extraction from a Holter monitor, the extraction ECG will be completed within 15 minutes prior to the collection of the blood sample.

The timing of blood samples drawn for pralsetinib concentration determination may be changed if the emerging data indicate that an alternate sampling scheme is needed for better characterization of the pralsetinib PK profile. Moreover, the total number of samples may be decreased if the initial sampling scheme is considered unnecessarily intensive. Should the number of required samples increase, the protocol and informed consent form (ICF) will be amended.

7.5 Pharmacodynamic and Biomarker Assessment

The RET proto-oncogene is relevant to the pathogenesis of several cancers, with thyroid cancer and NSCLC being the most prominent. RET activation mechanisms vary between cancer indications, with activating mutations being predominant in MTC and RET fusions predominant in PTC and NSCLC. Target engagement will be monitored through RET pathway specific biomarkers, such as quantification of DUSP6 and SPRY4

expression in tumors, RET allele burden in blood, as well as disease-specific secreted proteins (Calcitonin and CEA) in blood in patients with MTC.

7.5.1 Blood Samples for Pharmacodynamic and Biomarker Assessment

Blood samples will be collected at the time points outlined in Table 12 to characterize the mutant allele fraction in plasma circulating tumor deoxyribonucleic acid (ctDNA) of RET at baseline and during treatment with pralsetinib, and upon disease progression to measure changes from baseline in the levels of RET mutant allele fraction in all patients.

For patients with MTC, blood samples will be collected for measurement of calcitonin and CEA (performed by a central laboratory) as outlined in Table 12.

A blood sample will be collected for assessment of exploratory plasma markers: blood samples will be collected at the time points outlined in Table 12 and assayed at a central laboratory to identify potential markers (DNA, RNA, and/or protein) of pralsetinib activity. Additional exploratory biomarker research may be performed using residual blood samples and material derived from these samples in view of developing new genetic and mechanistic biomarkers of pralsetinib in patients with cancer.

7.5.2 Tumor Samples for RET Status and Biomarker Assessment

To meet the secondary objectives, tumor tissue will be obtained at the time points detailed in Table 12 to assess RET gene status, pharmacodynamics effects, and potential mechanisms of resistance.

Pretreatment tumor tissue (archived or new tissue) will be analyzed centrally for RET gene status. Patients enrolled in Phase 2, Group 6, will be required to have a pre-treatment biopsy to determine baseline RET gene status in tumor tissue. RET gene status will be assessed retrospectively in all patients.

Investigators should obtain new tumor tissue via core needle biopsy according to local institutional practices. Any new tumor biopsies done purely to obtain tissue for participation in this study should be performed using techniques and involving anatomical locations that are not a significant risk to the patient. If feasible, 2 cores should be obtained.

In Phase 1 only, the on-treatment tumor biopsy will be obtained to assess pharmacodynamic biomarker levels (eg, DUSP6 and SPRY4). On-treatment biopsy is strongly encouraged if it is safe and medically feasible.

An optional tumor biopsy obtained upon disease progression will be tested for potential mechanisms of resistance to pralsetinib.

Additional exploratory biomarker research may be performed using residual tumor tissue samples and material derived from these samples in view of developing new genetic and mechanistic biomarkers of pralsetinib in patients with cancer.

7.6 Disease Response Assessment

Investigator-assessed disease response (per RECIST v1.1 or RANO, if appropriate for tumor type) will be based on local imaging scans performed at the time points outlined in Table 10. Computed tomography with intravenous (IV) contrast of the chest (CT of the chest is preferred to avoid respiratory artifacts; however, MRI may be substituted if necessary), and CT or MRI with IV contrast of the abdomen, brain (MRI is preferred for brain imaging), and pelvis, will be performed. In addition, for patients with thyroid cancer, CT with IV contrast or MRI of the neck will be performed. At subsequent time points, all body regions that contained sites of disease (target or non-target) at screening will be imaged; therefore, regions such as the pelvis and brain do not need subsequent imaging if there is no disease at baseline.

If a patient is not tolerant to IV contrast, non-contrast CT or MRI may be performed. The CT portion of a PET/CT scan may be used as long as it is of diagnostic quality and with IV contrast (if the patient is tolerant of IV contrast). For each patient, the same method of tumor imaging used at baseline should be used.

CT scans and MRI will be reviewed locally at the study center, ideally by the same individual at each time point. CT scans and MRI will also be collected and reviewed centrally, to provide a central assessment of response and progression.

7.7 Patient-Reported Outcomes and Quality of Life Measurements

The EORTC QLQ-C30 is a 30-item questionnaire used to evaluate quality of life; it includes 5 functional domains (physical, cognitive, role, emotional, and social) and a global health status scale. Each subscale is evaluated on a standardized scale of 0 to 100 (Aaronson et al, 1993). Patients will complete the EORTC QLQ-C30 on D1 of Cycles 1 through 12. If the patient does not complete the questionnaire at Cycle 1 Day 1 (ie, for a baseline), then it should not be completed at subsequent cycles. A copy of the EORTC QLQ-C30 is provided in Appendix 7.

7.8 End-of-treatment and 30-Day Safety Follow-up

All patients will attend an End-of-treatment visit approximately 14 days (± 7 days) after the last dose of study drug (see Table 10 for a description of EOT assessments). If an alternate treatment is started within 14 days of the last dose of study drug, the EOT visit should be conducted prior to the first dose of alternate therapy. End-of-treatment procedures do not need to be repeated if they were completed within 7 days (or within 28 days for disease response assessments).

A Follow-up telephone contact for resolution of any residual AE will be made 30 days (+ 7 days) after the last dose of study drug, or at the time the patient initiates another antineoplastic therapy.

7.9 Progression Free Survival and Overall Survival Follow-up

Upon discontinuation of study treatment, patients without documented progressive disease will be followed for PFS, which will include tumor imaging every 3 to 4 months (±14 days; as per the schedule at the time of treatment discontinuation from the EOT visit until documentation of progressive disease, initiation of another antineoplastic therapy, or death.

Patients with documented progression will be followed for survival, which will include subsequent antineoplastic therapy (at the least, reporting of the first therapy received after discontinuation of pralsetinib) and survival status approximately every 3 months (from the EOT visit) until death, withdrawal of consent, or closure of the study by the Sponsor.

Patients who come off PFS follow-up due to progression will move on to survival follow-up which can be completed via telephone.

7.10 Sample Processing, Storage, and Shipment

Instructions for the processing, storage, and shipment of all study samples for central analysis will be provided in a separate study manual.

Samples will be stored until analysis and remaining samples and/or tissue will be retained until 10 years after completion of the study, or until the research is discontinued, whichever occurs first.

8 STUDY DRUG MANAGEMENT

8.1 Description

8.1.1 Formulation

Pralsetinib will be provided as capsules or immediate release tablets (if available) for oral administration. The drug produced is manufactured and formulated following current Good Manufacturing Practices (cGMP).

Each capsule contains 100 mg of the active drug substance, formulated in white, orange, and light blue opaque hard-gelatin capsules. Each tablet contains 100 mg of the active drug substance.

All study drugs are for investigational use only and should only be used within the context of this study.

8.1.2 Storage

Pralsetinib capsules or tablets (if available) must be stored in the container provided at room temperature (15-30°C; 59-86°F), out of direct light.

All study drug products must be stored in a secure, limited-access location and may be dispensed only by the Investigator or by a member of the staff specifically authorized by the Investigator.

8.2 Packaging and Shipment

Capsules and/or tablets will be supplied in high-density polyethylene (HDPE) bottles containing a desiccant and a child-resistant closure.

Packaging will meet all regulatory requirements.

8.3 Accountability

Accountability for the study drug at the study center is the responsibility of the Investigator. The Investigator will ensure that the study drug is used only in accordance with this protocol. Where allowed, the Investigator may choose to assign drug accountability responsibilities to a pharmacist or other appropriate individual. Study drug dispensation will occur at the study center during each applicable visit; however, in the event that the patient cannot travel to the study center for resupply, shipment of study drug to the patient may be allowed, upon Sponsor approval. Direct to patient shipment of study drug from the study center should be appropriately documented by the site.

The Investigator or delegate will maintain accurate drug accountability records indicating the drug's delivery date to the site, inventory at the study center, use by each patient, and return to the Sponsor or its designee (or disposal of the drug, if approved by the Sponsor). These records will adequately document that the patients were provided the doses as specified in the protocol and should reconcile all study drug received from the Sponsor. Accountability records will include dates, quantities, batch/serial numbers, expiration dates (if applicable), and patient numbers. The Sponsor or its designee will review drug accountability at the study center on an ongoing basis during monitoring visits.

Study drug must not be used for any purpose other than the present study. Study drug that has been dispensed to a patient and returned unused must not be re-dispensed to a different patient.

Patients will receive instructions for home administration of pralsetinib.

All unused and used study drug should be retained at the center until they are inventoried by the study monitor. All used, unused, or expired study drug will be returned to the Sponsor or its designee, or if authorized, disposed of at the study center per the center's Standard Operating Procedures and documented. All material containing pralsetinib will be treated and disposed of as hazardous waste in accordance with governing regulations.

8.4 Compliance

Patients will be dispensed the appropriate number of study drug bottles to allow for dosing for at least a full cycle, or until the next scheduled visit. Patients are to return all unused study drug (or the empty bottles) on D1 of each treatment cycle or at the next

scheduled visit. Compliance with the dosing regimen will be assessed based on return of unused drug (or empty bottles).

9 ADVERSE EVENTS

Monitoring of AEs will be conducted throughout the study. Adverse events will be recorded in the eCRF from the time of first study drug dose through 30 days after the last study drug dose. Serious AEs and serious pretreatment events (see Section 9.5) will be recorded in the eCRF from the time of signing informed consent (for the full study, not the tissue screening ICF) through 30 days after the last study drug dose. In addition, SAEs that are assessed as related to study treatment and that occur > 30 days post-treatment will also be reported. All related AEs should be monitored until they are resolved, stabilized, have returned to pre-exposure baseline, determined to be due to another illness, or until a subsequent therapy is initiated. For AEs considered not related to study drug, similar monitoring guidelines will only be required through 30 days after the last dose of study drug.

9.1 Definitions

9.1.1 Definition of Adverse Event

An **adverse event (AE)** is any untoward medical occurrence associated with the use of a drug in humans, whether or not considered drug-related. An AE (also referred to as an adverse experience) can be any unfavorable and unintended sign (eg, an abnormal laboratory finding), symptom, or disease temporally associated with the use of a drug, without any judgment about causality. An AE can arise from any use of the drug (eg, off-label use, use in combination with another drug) and from any route of administration, formulation, or dose, including an overdose. Overdose includes only clinically symptomatic doses that are at least twice the intended dose.

An abnormal laboratory value will not be assessed as an AE unless that value leads to discontinuation or delay in treatment, dose modification, therapeutic intervention, or is considered by the Investigator to be clinically significant.

Clinical signs/symptoms or diagnoses assessed as due to disease progression should not be reported as an AE/SAE or cause of death. Instead, “disease progression” as an event term can be reported as an AE/SAE (if meeting the definition of AE/SAE as described above) or cause of death. In situations when signs/symptoms or a diagnosis cannot be clearly or only attributed to disease progression, it can be reported as an AE or SAE.

9.1.2 Suspected Adverse Reaction

A suspected adverse reaction is any AE that is not clearly caused by something other than pralsetinib.

9.1.3 Unexpected Adverse Event

An unexpected AE is one for which the nature or severity of the event is not consistent with the applicable product information (eg, the IB).

9.2 Documenting Adverse Events

Each patient must be carefully monitored for the development of any AEs. This information should be obtained in the form of non-leading questions (eg, “How are you feeling?”) and from signs and symptoms detected during each examination, observations of study personnel, and spontaneous reports from patients.

All AEs (serious and non-serious) spontaneously reported by the patient and/or in response to an open question from study personnel or revealed by observation, physical examination, or other diagnostic procedures will be recorded in the appropriate section of the eCRF. Any clinically significant value in laboratory assessments or other clinical findings is considered an AE and must be recorded on the appropriate pages of the eCRF. A laboratory value may also be considered an AE when it qualifies as an SAE, when it requires medical intervention, or when it affects dosing with study drug. When possible, signs and symptoms indicating a common underlying pathology should be noted as one comprehensive event reflecting the final clinical diagnosis.

9.3 Assessment of Intensity

Intensity of all AEs, including clinically significant treatment-emergent laboratory abnormalities, will be graded according to the National Cancer Institute (NCI) Common Terminology Criteria for Adverse Events (CTCAE), version 4.03. Adverse events not listed in the CTCAE will be graded as follows:

- Grade 1: Mild, the event is noticeable to the patient but does not interfere with routine activity.
- Grade 2: Moderate, the event interferes with routine activity but responds to symptomatic therapy or rest.
- Grade 3: Severe, the event significantly limits the patient’s ability to perform routine activities despite symptomatic therapy.
- Grade 4: Life-threatening, an event in which the patient was at risk of death at the time of the event.
- Grade 5: Fatal, an event that results in the death of the patient.

9.4 Assessment of Causality

Relationship to study drug administration will be determined by the Investigator according to the following criteria:

- Not Related: Exposure to the study treatment did not occur, or the occurrence of the AE is not reasonably related in time, or the AE is clearly caused by something other than pralsetinib.

- Related: The AE does not meet the criteria for “Not Related”.

At the time of the initial report, based on the available information and medical judgment, causality must be reported as either related or not related, even if there is insufficient information. Once additional information is available, Investigator’s causality assessment can be updated.

9.5 Pretreatment Events

A pretreatment event is any untoward occurrence in a patient who has signed informed consent to participate in a study but before administration of any study drug. It does not necessarily have a causal relationship with study participation. A serious pretreatment event meets the criterion of a pretreatment event and satisfies any of the 6 criteria specified for an SAE as described in Section 10.

9.6 Pregnancy Reporting

Pregnancy is neither an AE nor an SAE, unless a complication relating to the pregnancy occurs (eg, spontaneous abortion, which may qualify as an SAE).

Pregnancies and suspected pregnancies (including a positive pregnancy test regardless of age or disease state) of a female patient or partner of a male patient occurring while the patient is on study drug, or within 30 days of the patient’s last dose of study drug, are considered immediately reportable events. If a female partner of a male patient taking investigational product becomes pregnant, the male patient taking pralsetinib should notify the Investigator, and the pregnant female partner should be advised to call her healthcare provider immediately. In pregnant female patients, study drug is to be discontinued immediately and the patient instructed to return any unused pralsetinib to the Investigator. The pregnancy, suspected pregnancy, or positive pregnancy test must be reported immediately using the Pregnancy Report Form. The Investigator must follow up and document the course and outcome of all pregnancies even if the patient was discontinued from the study or if the study has finished. The female patient or partner of a male patient should receive any necessary counseling regarding the risks of continuing the pregnancy and the possible effects on the fetus. Monitoring should continue until conclusion of the pregnancy.

All outcomes of pregnancy must be reported by the Investigator to the Sponsor or Medical Monitor on a Pregnancy Outcome Report form within 30 days after he/she has gained knowledge of the delivery or elective abortion.

Any SAE that occurs during pregnancy must be recorded on the SAE report form (eg, maternal serious complications, spontaneous or therapeutic abortion, ectopic pregnancy, stillbirth, neonatal death, congenital anomaly, or birth defect) and reported within 24 hours in accordance with the procedure for reporting SAEs.

9.6.1 Contraception Requirements

Women of childbearing potential must agree to use a highly effective method of contraception from the time of giving informed consent until at least 30 days after the last dose of pralsetinib. Women are considered to be of childbearing potential following menarche until becoming postmenopausal (defined as no menses for at least 12 months without an alternative medical cause) unless permanently sterile. A high follicle-stimulating hormone (FSH) level in the postmenopausal range may be used to confirm a postmenopausal state in women not using hormonal contraception or hormonal replacement therapy. However, in the absence of 12 months of amenorrhea, a single FSH measurement is insufficient. Permanent sterilization methods include hysterectomy, bilateral salpingectomy and bilateral oophorectomy (CTFG).

Male patients with partners who are female of reproductive potential must agree that they will use condoms, and their partners will use a highly effective contraceptive method throughout the study, and for 90 days after the last dose of pralsetinib.

Highly effective forms of contraception are defined as the following (CTFG):

- Combined (estrogen and progestogen containing) hormonal contraceptives that inhibit ovulation, including oral, intravaginal and transdermal products.
- Progestogen-only hormonal contraceptives that inhibit ovulation, including oral, injectable, and implantable products.
- Intrauterine devices (IUD) and intrauterine hormone-releasing system (IUS).
- Bilateral tubal occlusion (women).
- Male partner vasectomy or other method of surgical sterilization provided that the partner is the sole sexual partner of trial participant and the vasectomized partner has received medical assessment of the surgical success.
- Sexual abstinence (men and women), when this is the preferred and usual lifestyle of the patient. Periodic abstinence (such as calendar, symptothermal and post-ovulation methods), withdrawal (coitus interruptus), and the lactational amenorrhea method are not acceptable methods of contraception.

The following methods of contraception are not considered highly effective (CTFG):

- Progesterone-only oral hormonal contraception that do not inhibit ovulation.
- Barrier methods with or without spermicide, or spermicide alone.

9.6.2 Gamete and Embryo Banking

Formal reproductive toxicology experiments have not been performed. Patients should be informed of the possibility of gamete and embryo banking.

10 SERIOUS ADVERSE EVENTS

10.1 Definition of Serious Adverse Event

An SAE is any event that meets any of the following criteria:

- Death.
- Life-threatening.

  An AE is life-threatening if the patient was at immediate risk of death from the event as it occurred (ie, it does not include a reaction that if it had occurred in a more serious form might have caused death). For example, drug-induced hepatitis that resolved without evidence of hepatic failure would not be considered life-threatening even though drug-induced hepatitis can be fatal.

- Inpatient hospitalization or prolongation of existing hospitalization.

  Adverse events requiring hospitalization should be considered SAEs. Hospitalization for elective surgery or routine clinical procedures that are not the result of AE (eg, elective surgery for a pre-existing condition that has not worsened) need not be considered AEs or SAEs. If anything untoward is reported during the procedure, that occurrence must be reported as an AE, either 'serious' or 'non-serious' according to the usual criteria.

  In general, hospitalization signifies that the patient has been admitted (usually involving at least an overnight stay) to the hospital or kept in the emergency room for observation and/or treatment that would not have been appropriate in the physician’s office or outpatient setting. When in doubt as to whether 'hospitalization' occurred or was necessary, the AE should be considered serious.

- Persistent or significant disability/incapacity.

  An AE is incapacitating or disabling if the experience results in a substantial and/or permanent disruption of the patient's ability to carry out normal life functions.

- Congenital anomaly/birth defect in the offspring of a patient or patient’s partner who received pralsetinib.
- Other: Important medical events that may not result in death, be life-threatening, or require hospitalization, may be considered an SAE when, based upon appropriate medical judgment, they may jeopardize the patient and may require medical or

surgical intervention to prevent one of the outcomes listed in this definition. Examples of such events are:

- Intensive treatment in an emergency room or at home for allergic bronchospasm.
- Blood abnormalities or convulsions that do not result in inpatient hospitalization.
- Development of drug dependency or drug abuse.

10.2 Reporting Serious Adverse Events

All SAEs or serious pretreatment events that occur during the course of the study must be promptly reported by the Investigator to Blueprint Medicines. Deaths and AEs assessed as life-threatening are to be reported immediately and SAEs that meet other criteria are to be reported within 24 hours from the point in time when the Investigator becomes aware of the SAE. All SAEs must be reported whether or not they are considered causally related to pralsetinib or protocol procedures. Serious AE forms will be completed and the information collected will include patient number, a narrative description of the event, and an assessment by the Investigator as to the severity of the event and relatedness to study drug. Follow-up information on the SAE may be requested by the Sponsor or Medical Monitor.

[redacted]

If there are serious, unexpected adverse drug reactions associated with the use of pralsetinib, the Sponsor will notify the appropriate regulatory agency(ies) and all participating Investigators on an expedited basis. The local Institutional Review Board (IRB)/ Independent Ethics Committee (IEC) will be promptly notified based on local regulations where required by the IRB/IEC of all serious, unexpected adverse drug reactions involving risk to human patients.

All AEs, whether serious or not, will be described in the source documents and in the appropriate section of the eCRF. All new events, as well as those that worsen in intensity or frequency relative to baseline, which occur after signing the informed consent through 30 days following the last dose of study drug, must be recorded. Adverse events that are ongoing at the time of treatment discontinuation should be followed through the 30-day follow-up assessment. Serious AEs felt by the Investigator to be related to pralsetinib, however, must be reported any time the Investigator becomes aware of such an event, even if this occurrence is more than 30 days after the last dose of study drug.

Information to be reported in the description of each AE includes:

- A medical diagnosis of the event (if a medical diagnosis cannot be determined, a description of each sign or symptom characterizing the event should be recorded).
- The date of onset of the event.
- The date of resolution of the event, if available.
- Whether the event is serious or not.
- Intensity of the event (see Section 9.3 for definitions).
- Relationship of the event to study treatment or protocol procedure (see Section 9.4 for definitions).
- Action taken: drug withdrawn; drug temporarily interrupted; dose reduced; dose not changed; unknown; or not applicable.
- Outcome: recovered/resolved; not recovered/not resolved; recovered/resolved with sequelae; fatal (notify the Medical Monitor immediately, and complete the SAE form); or unknown.

10.2.1 Overdose

Overdose is defined as a clinically symptomatic dose that is at least twice the intended dose. Any instance of overdose (suspected or confirmed and irrespective of whether or not it involved pralsetinib) must be communicated to Blueprint Medicines or a specified designee within 24 hours and be fully documented as an SAE. Details of any signs or symptoms and their management should be recorded including details of any antidote(s) administered.

11 STATISTICS

11.1 General Procedures

Statistical analyses of safety, PK, and pharmacodynamics will be primarily descriptive in nature. Antineoplastic activity will be analyzed and presented descriptively. Hypothesis testing will be performed based on the pre-specified hypotheses for the dose expansion part (Phase 2) of the study.

Continuous variables will be summarized using descriptive statistics (n, mean, standard deviation, median, minimum, and maximum).

Categorical variables will be summarized showing the number and percentage (n, %) of patients within each classification. Appropriate CIs will also be presented.

Safety, PK, pharmacodynamics, and efficacy will be assessed in the appropriate populations accordingly. Patients treated at MTD/RP2D in Phase 1 will be pooled with Phase 2 patients accordingly.

All data will be provided in by-patient listings.

11.2 Analysis Populations

The following analysis populations will be used for presentation of the data:

- Safety Population: All patients who have received at least 1 dose of study drug regardless of starting dose levels. The Safety Population will be the primary population for safety analysis, unless otherwise specified. Patients will be analyzed based on the initial dose prescribed on Day 1, regardless of phase.

- Efficacy Population: All patients who have been exposed to at least 1 dose of study drug at the RP2D excluding patients previously treated with selective RET-inhibitor, such as selpercatinib. Efficacy Population will be the primary population for analysis of efficacy endpoints (except for ORR, DOR, CBR, DCR). Patients will be analyzed based on the initial dose prescribed on Day 1, regardless of phase.

- Dose-Determining Population: All patients in the dose-escalation part who have received ≥ 75% (21 days) of study drug and completed safety evaluations through C1D28 or experienced a DLT.

- “RET-altered” Measurable Disease Population: Efficacy Population patients who have measurable (target) disease per RECIST v1.1 (or RANO, if appropriate for tumor type) at baseline according to blinded central review and sufficient evidence of a RET alteration. The “RET-altered” Measurable Disease Population will be the primary population for analysis of ORR, DOR, CBR, DCR.

- Response-Evaluable (RE) Population: Efficacy Population patients who have measurable (target) disease per RECIST v1.1 (or RANO, if appropriate for tumor type) at baseline according to blinded central review--, have at least one evaluable post-baseline disease response assessment performed, and have experienced no major protocol violations. The RE Population will be used as a sensitivity analysis for ORR, DOR, CBR, and DCR.

- PK Population: All patients who have adequate PK samples collected so that the PK parameters can be estimated.

- ECG Population: All patients who underwent continuous Holter monitoring and have sufficient ECG tracings so that QT interval corrected for heart rate (QTc) parameter can be evaluated.

11.3 Sample Size

11.3.1 Phase 1 (Dose-escalation)

The dose escalation part of the study (Phase 1) employs the local BOIN design (Liu and Yuan, 2015) to find the MTD. The BOIN design is implemented in a simple way similar to the traditional 3+3 design but is more flexible and possesses superior operating characteristics that are comparable to those of the more complex model-based designs, such as the CRM.

The target toxicity rate for the MTD is 30%. Alternatives under which decision errors are minimized are: 18% (subtherapeutic) and 42% (overly toxic). To avoid assigning too many patients to the overly toxic doses, a dose level will be eliminated (and any planned doses that are higher) when the posterior probability is greater than 95% that the toxicity rate of a dose level is > 30%, given the number of patients (≥ 3) and DLTs observed at the dose level. Dose escalation will continue to enroll and treat patients in cohorts of size 3-6 (1-3 for the first 3 dose levels) until 12 patients are treated and evaluable for DLT at one dose level. The total number of patients to be enrolled during the dose escalation part of the study will vary depending on the underlining dose-toxicity curve and the number of dose levels tested prior to reaching MTD and is estimated to be approximately 20 to 50 patients.

Dose-escalation rules are detailed in Section 6.3.1.2.

After Phase 1 is completed, the MTD will be selected based on isotonic regression as specified by Liu and Yuan (Liu and Yuan, 2015). Specifically, MTD will be selected as the dose for which the isotonic estimate of the toxicity rate is closest to the target toxicity rate. If there are ties, the higher dose level is selected when the isotonic estimate is lower than the target toxicity rate; and the lower dose level is selected when the isotonic estimate is greater than the target toxicity rate.

11.3.1.1 Operating characteristics

Appendix 4 shows the operating characteristics of the trial design based on 100,000 simulations of the trial. For trial simulation purposes the target toxicity rate for the MTD is assumed to be 30% (0.3) and the maximum sample size is 99. Further assumptions include enrolling and treating 3 patients per cohort with a total of 13 dose levels, and that the true toxicity curves are monotonic and cover a wide range of possibilities (as shown in Figure 9). The operating characteristics show that the design selects the true MTD with high probabilities and allocates more patients to the dose levels with the DLT rate closest to the target of 30%.

Figure 9: Dose-toxicity Scenarios

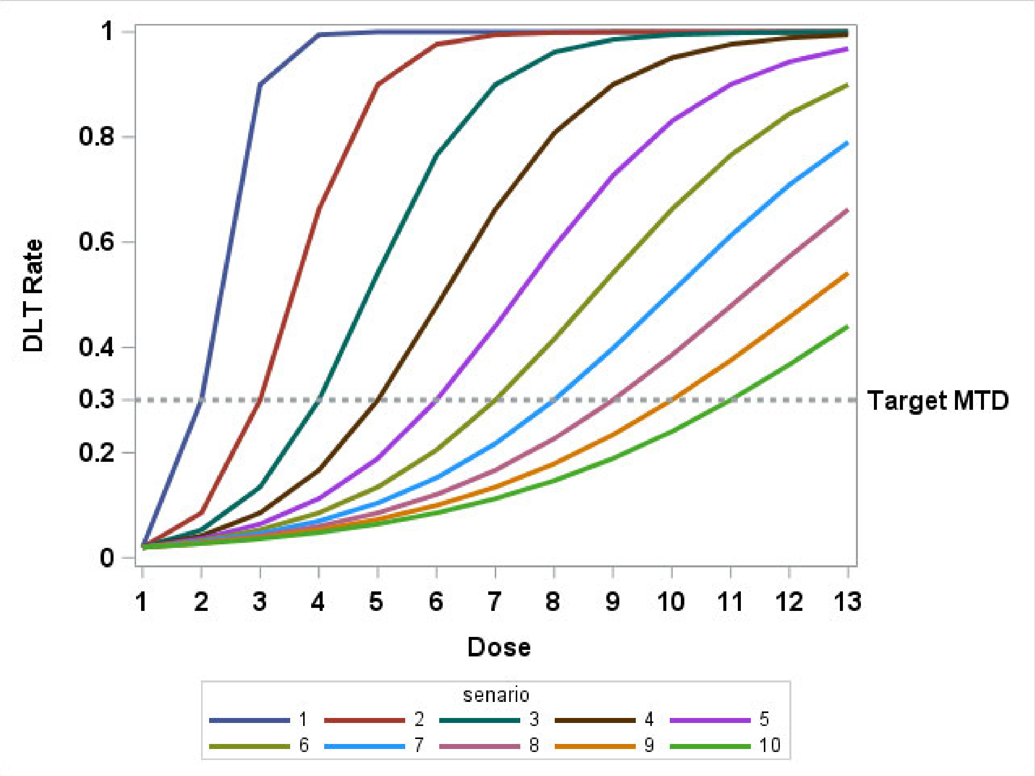

Abbreviations: BOIN = Bayesian optimal interval, DLT = dose-limiting toxicity, MTD = maximum tolerated dose

Note: scenario number shown in this figure matches the scenario number in Appendix 4‘Operating Characteristics of the BOIN design’

11.3.2 Phase 2 (Expansion)

As pralsetinib has potent activity against RET fusions, activating mutations and resistance mutations, the hypothesis and sample size calculation based on ORR as per RECIST v1.1 (or RANO, if appropriate for tumor type) is specific to the response-evaluable RET-altered patients (excluding groups 6 and 7) treated at the MTD/RP2D for each Phase 2 expansion group.

- Group 1: The sample size of approximately 80 RET-fusion NSCLC patients who have failed treatment with a prior platinum-based chemotherapy will provide >95% power at the 2-sided significance level of 0.05 for testing the assumption of the null hypothesis ORR=0.23 versus the alternative ORR=0.5.
- Group 2: The sample size of approximately 170 treatment naïve (1st line) RET-fusion NSCLC patients will provide >90% power at the 2-sided significance level of 0.05 for testing the assumption of the null hypothesis ORR=0.48 versus the alternative ORR=0.61.
- Group 3: The sample size of approximately 65 MTC patients previously treated with cabozantinib and/or vandetanib will provide >90% power at the 2-sided significance

level of 0.05 for testing the assumption of the null hypothesis ORR=0.2 versus the alternative ORR=0.4.

- Group 4: The sample size of approximately 40 MTC patients not previously treated with cabozantinib or vandetanib will be enrolled for exploratory analysis; however, see Table 16 for details regarding confidence intervals.
- Group 5: The sample size of approximately 100 patients who have solid tumors with a RET fusion previously treated with SOC, have previously received SOC appropriate for their tumor type (unless there is no accepted standard therapy for the tumor type or the Investigator has determined that treatment with standard therapy is not appropriate), and must not eligible for any of the other groups. This sample size will provide >90% power at the 2-sided significance level of 0.05 for testing the assumption of the null hypothesis ORR=0.1 versus the alternative ORR=0.3.
- Group 6: The sample size of approximately 20 patients who have RET-fusion solid tumors that failed prior selective-RET inhibitor will be enrolled for exploratory analysis; however, see Table 19 for details regarding confidence intervals.
- Group 7: The sample size of approximately 20 patients who have solid tumors with an activating RET mutation previously treated with SOC will be enrolled for exploratory analysis; however, see Table 19 for details regarding confidence intervals.
- Group 8: The sample size of approximately 30 RET-fusion NSCLC patients that have failed treatment with a prior platinum-based chemotherapy will be enrolled, see Table 20 for details regarding confidence intervals (an observed ORR of ≥ 27% in 30 patients will result in an exact binomial 95% CI with a lower bound greater than 10%.)
- Group 9: The sample size of approximately 30 MTC patients that were not previously treated with systemic therapy (except prior cytotoxic chemotherapy is allowed) for advanced or metastatic disease will be enrolled, however see Table 20 for details regarding confidence intervals (an observed ORR of ≥ 27% in 30 patients will result in an exact binomial 95% CI with a lower bound greater than 10%.)

With approximately 590 patients in the Safety Population, there is > 97% probability of observing at least 3 AEs that occur at a frequency of 0.5%; there is > 99% probability of observing at least 5 AEs that occur at a frequency of 1%.

11.4 Statistical Methods

Patients enrolled in Phase 1 and treated at the RP2D will be pooled together with the appropriate Phase 2 group patients for analyses. The analysis based on Safety Population, Efficacy Population, RET-altered Measurable Disease Population, and RE Population will be conducted according to patients' disease type, and/or RET-altered status if applicable, and/or prior treatment status, if appropriate. The analysis based on Dose-Determining Population will be conducted according to patients' initial prescribed dose level. Efficacy endpoints will be analyzed for the Efficacy Population (PFS and OS) and RET-altered Measurable Disease Population (ORR, DOR, CBR, and DCR). Safety endpoints will be analyzed for Safety population. The RE Population will be the basis for

sensitivity analyses of efficacy endpoints. In addition, for the efficacy endpoints, subgroup analyses will be completed for patients enrolled in the treatment-naïve Groups 2 (NSCLC) and 4 (MTC), consisting of 1) patients enrolled prior to Protocol Amendment 9 (who were not candidates for standard therapies and thus may have had a poor prognosis) and 2) those enrolled on or after Protocol Amendment 9 (possibly a more typical first-line population).

11.4.1 Demographic and Baseline Characteristics

Demographic and baseline disease characteristic data will be summarized descriptively.

11.4.2 Primary Endpoints

11.4.2.1 Phase 1

1. MTD and RP2D

Tabulations of the incidence of DLTs for the DLT evaluable population at each dose level will be provided.

2. Safety

Safety evaluations will be based on the incidence, severity, and type of AEs, and changes in the patient's physical examination findings, vital signs, clinical laboratory results, and ECG findings. Safety variables will be tabulated and presented for all patients of the safety population (combined Phase 1 and Phase 2), and/or by disease type, and/or RET-altered status, and/or prior treatment status if applicable. Summarization will focus on incidence of any SAEs, AEs by system organ class and preferred term, and discontinuation rates of study drug due to AE or toxicity based on clinical laboratory results.

Adverse events will be assessed for intensity according to the NCI CTCAE, version 4.03, and the verbatim AE term will be coded using the Medical Dictionary for Regulatory Activities (MedDRA) for purposes of summarization.

Treatment-emergent AEs will be tabulated, where treatment-emergent is defined as any AE that occurs during or after administration of the first dose of study drug through 30 days after the last dose of study drug, any event that is considered study drug-related regardless of the start date of the event, or any event that is present at baseline but worsens in intensity or is subsequently considered study drug-related by the Investigator.

11.4.2.2 Phase 2

Patients enrolled in Phase 1 and treated at the RP2D will be pooled together with the appropriate Phase 2 group patients for analyses.

1. Efficacy

The ORR, defined as proportion of patients with best overall response of confirmed complete response (CR) or partial response (PR), will be summarized by disease type, and/or RET-altered status if applicable, and/or prior treatment status in Phase 2 (including patients treated at the MTD/RP2D in Phase 1). The 95% CI based on the exact binomial distribution will also be provided.

An observed ORR of ≥ 35% in 80 patients will result in an exact binomial 95% CI with a lower bound greater than 30% (Table 13).

Table 13: Confidence Interval of Observed Response Rate for Group 1, N = 80

Observed ORR	Number of Confirmed CR or PR	Exact 95% CI	Exact 97.5%
35%	28	(24.7, 46.5)	(23.4, 48.1)
40%	32	(29.2, 51.6)	(27.8, 53.1)
45%	36	(33.8, 56.5)	(32.4, 58.1)
50%	40	(38.6, 61.4)	(37.1, 62.9)

Abbreviations: CI = confidence interval; CR = complete response; ORR = overall response rate; PR = partial response.

An observed ORR of ≥ 20% in 170 patients will result in an exact binomial 95% CI with a lower bound greater than 48% (Table 14).

Table 14: Confidence Interval of Observed Response Rate for Group 2, N=170

Observed ORR	Number of Confirmed CR or PR	Exact 95% CI	Exact 97.5%
56%	96	(48.7, 64)	(47.6, 65.1)
57%	97	(49.3, 64.6)	(48.2, 65.6)
58%	99	(50.4, 65.7)	(49.4, 66.7)
59%	101	(51.6, 66.9)	(50.5, 67.9)
60%	102	(52.2, 67.4)	(51.1, 68.4)
61%	104	(53.4, 68.5)	(52.3, 69.5)

An observed ORR of ≥ 20% in 65 patients will result in an exact binomial 95% CI with a lower bound greater than 20% (Table 15).

Table 15: Confidence Interval of Observed Response Rate for Group 3, N = 65

Observed ORR	Number of Confirmed CR or PR	Exact 95% CI	Exact 97.5%
32%	21	(21.2, 45.1)	(19.9, 46.8)
34%	22	(22.6, 46.6)	(21.2, 48.4)
37%	24	(25.3, 49.8)	(23.8, 51.5)
40%	26	(28, 52.9)	(26.5, 54.6)

Abbreviations: CI = confidence interval; CR = complete response; ORR = overall response rate; PR = partial response.

An observed ORR of ≥ 28% in 40 patients will result in an exact binomial 95% CI with a lower bound greater than 10% (Table 16).

Table 16: Confidence Interval of Observed Response Rate for Group 4, N = 40

Observed ORR	Number of Confirmed CR or PR	Exact 95% CI	Exact 97.5%
28%	11	(14.6, 43.9)	(13.2; 46.1)
30%	12	(16.6, 46.5)	(15.1, 48.8)
35%	14	(20.6, 51.7)	(19.0, 53.9)
40%	16	(24.9, 56.7)	(23.1, 58.8)
45%	18	(29.3, 61.5)	(27.4, 63.6)
50%	20	(33.8, 66.2)	(31.8, 68.2)

Abbreviations: CI = confidence interval; CR = complete response; ORR = overall response rate; PR = partial response.

Table 17 provides the probability of observing responders for a given range of response rate for Group 5.

Table 17: Probability of Observing Responses for Group 5, N = 100

Number of Responses Observed	True Response Rate			
	20%	30%	40%	50%
≥ 10	0.99	>0.99	>0.99	1
≥ 15	0.87	>0.99	>0.99	1
≥ 20	0.44	0.98	>0.99	>0.99
≥ 25	0.08	0.84	>0.99	>0.99
≥ 30	0.01	0.45	0.98	>0.99

An observed ORR of ≥ 17% in 100 patients will result in an exact binomial 95% CI with a lower bound greater than 10%

Table 18: Confidence Interval of Observed Response Rate for Group 5, N = 100

Observed ORR	Number of Confirmed CR or PR	Exact 95% CI	Exact 97.5%
17%	17	(10.2, 25.8)	(9.5, 27.1)
20%	20	(12.7, 29.2)	(11.8, 30.5)
25%	25	(16.9, 34.7)	(15.9, 36)
30%	30	(21.2, 40)	(20.2, 41.4)
35%	40	(25.7, 45.2)	(24.6, 46.6)
40%	45	(30.3, 50.3)	(29.1, 51.7)

Abbreviations: CI = confidence interval; CR = complete response; ORR = overall response rate; PR = partial response.

Table 19 provides the probability of observing responders for a given range of response rate.

Table 19: Probability of Observing Responses for Groups 6 and 7, N = 20

Number of Responses Observed	True Response Rate		
	**20%**	**30%**	**50%**
≥ 1	0.99	0.99	0.99
≥ 2	0.93	0.99	0.99
≥ 3	0.79	0.96	0.99
≥ 4	0.59	0.89	0.99
≥ 5	0.37	0.76	0.99
≥ 6	0.20	0.58	0.98

Table 20: Confidence Interval of Observed Response Rate for Groups 8 and 9, N = 30

Observed ORR	Number of Confirmed CR/PR	Exact 95% CI	Exact 98.75% CI
27%	8	(12.3, 45.9)	(9.6, 50.8)
30%	9	(14.7, 49.4)	(11.8, 54.3)
33%	10	(17.3, 52.8)	(14.1, 57.6)
40%	12	(22.7, 59.4)	(19.0, 64.0)
43%	13	(25.5, 62.6)	(21.6, 67.1)
50%	15	(31.3, 68.7)	(27.1, 72.9)

2. Safety

See Section 11.4.2.1 for safety analysis details.

11.4.3 Secondary Endpoints

11.4.3.1 Phase 1

1. Efficacy:

Overall response rate will be analyzed by disease type, and/or RET-altered status if applicable, and/or prior treatment status if appropriate. CBR, DCR, DOR, PFS, and OS and the correlation between RET gene status and antineoplastic activity will be defined as the following.

- CBR is defined as the rate of CR or PR, or stable disease which stable disease has been lasting more than 4 cycles (ie, 16 weeks) from the first dose date.
- DCR is defined as the rate of CR or PR, or stable disease, as per RECIST v1.1 (or RANO, if appropriate). CBR, DCR will be summarized using the same methods as ORR.
- For patients who achieve CR or PR per RECIST v1.1 (or RANO, if appropriate), DOR is defined as the time from either CR or PR (whichever response is recorded first), until the first date that disease progression is objectively documented, or until death (if death occurs within 2 scheduled assessments of the last evaluable scan). The DOR will be summarized descriptively using the Kaplan-Meier (KM) method. The median (95% CI) will be provided, if estimable.
- PFS is defined as the time from the first dose pralsetinib to the date of disease progression or death due to any cause (whichever is earlier). Patients who initiate

alternate anticancer therapy in the absence of documented progression will be censored at the latest disease assessment prior to the alternate therapy.

- OS is defined as the time from the first dose pralsetinib to the date of death due to any cause. Patients who are still alive or lost to follow-up will be censored at last known date. The censoring date will be determined from patients' last known contact date.

Progression-free survival and OS will also be summarized using the KM method for the efficacy population by disease type, and/or RET-altered status if applicable, and/or prior treatment status if appropriate. KM plots will be provided. Waterfall plots for maximum percentage of tumor reduction from baseline will also be provided. The probability of PFS and OS at certain timepoints (eg, 3, 6, 9, and 12-months etc.) will be estimated.

The ORR, CBR, DCR, DOR, and other measures of antineoplastic activity by major RET-altered gene status will be summarized. The correlation between RET-altered gene status and antineoplastic activity ORR, CBR, and DCR, will be analyzed in a same manner, if appropriate. The correlation between RET gene status and DOR may be explored with log rank model.

2. Pharmacokinetics

Pharmacokinetic parameters for pralsetinib will be calculated from the plasma concentration-time data using standard non-compartmental methods. Pharmacokinetic parameters of interest will include, as appropriate, maximum plasma drug concentration (C_{max}), time to maximum plasma drug concentration (T_{max}), time of last quantifiable plasma drug concentration (T_{last}), area under the plasma concentration versus time curve from time 0 to 24 hours postdose (AUC_{0-24}), plasma drug concentration at 24 hours postdose (C_{24}); apparent volume of distribution (Vz/F), terminal elimination half-life ($t_{½}$), apparent oral clearance (CL/F), and accumulation ratio (R). Descriptive statistics (ie, n, mean, standard deviation [SD], percent coefficient of variation [%CV], minimum, median, maximum, geometric mean [GeoMean], GeoMean %CV, and 95% CIs) will be used to summarize PK parameters for each dose level as appropriate.

3. Pharmacodynamics:

Pharmacokinetic/pharmacodynamic relationships will be descriptively analyzed within each dose level from paired PK/pharmacodynamic sample results for blood (eg, calcitonin, CEA, and ct DNA of RET) and tumor (eg, DUSP6/SPRY4) markers, if feasible.

11.4.3.2 Phase 2

Patients enrolled in Phase 1 and treated at the RP2D will be pooled together with the appropriate Phase 2 group patients for analyses.

1. Efficacy

CBR, DCR, DOR, PFS, and OS and the correlation between RET gene status (ie, gene fusion partner or primary mutation and, for MTC, whether hereditary or sporadic) and antineoplastic activity will be defined as the following.

- CBR is defined as the rate of CR or PR, or stable disease which stable disease has been lasting more than 4 cycles (ie, 16 weeks) from the first dose date.

- DCR is defined as the rate of CR, PR, and stable disease, as per RECIST v1.1 (or RANO, if appropriate). CBR, DCR will be summarized using the same methods as ORR.

- For patients who achieve CR or PR per RECIST v1.1 (or RANO, if appropriate), DOR is defined as the time from either CR or PR (whichever response is recorded first), until the first date that disease progression is objectively documented, or until death (if death occurs within 2 scheduled assessments of the last evaluable scan). The DOR will be summarized descriptively using the Kaplan-Meier (KM) method. The median (95% CI) will be provided, if estimable.

- PFS is defined as the time from the first dose pralsetinib to the date of disease progression or death due to any cause (whichever is earlier). Patients who initiate alternate anticancer therapy in the absence of documented progression will be censored at the latest disease assessment prior to the alternate therapy.

- OS is defined as the time from the first dose pralsetinib to the date of death due to any cause. Patients who are still alive or lost to follow-up will be censored at last known date. The censoring date will be determined from patients' last known contact date.

Progression-free survival and OS will also be summarized using the KM method for the safety population; KM plots will be provided. Waterfall plots for maximum percentage of tumor reduction from baseline will also be provided. The median of PFS and OS, as well as rates at certain timepoints (eg, 3, 6, 9, and 12-months), will be estimated for all patients in the safety population, as well as patients in the safety population who were treated at the MTD/RP2D, using the KM method. In addition, the OS follow-up analysis will be provided using the reverse KM method.

Antineoplastic activity, as measured by ORR, DOR, and DCR per RECIST v1.1 (or RANO, if appropriate); PFS may be analyzed centrally and summarized descriptively for all NSCLC and MTC patients in Parts 1 and 2 accordingly.

The ORR, CBR, DCR, DOR, and other measures of antineoplastic activity by major RET-altered gene status will be summarized.

2. Pharmacokinetics

Pharmacokinetic parameters for pralsetinib will be calculated from the plasma concentration-time data using standard non-compartmental methods. Pharmacokinetic

parameters of interest will include, as appropriate, maximum plasma drug concentration (C_{max}), time to maximum plasma drug concentration (T_{max}), time of last quantifiable plasma drug concentration (T_{last}), area under the plasma concentration versus time curve from time 0 to 24 hours postdose (AUC_{0-24}), plasma drug concentration at 24 hours postdose (C_{24}); apparent volume of distribution (V_z/F), terminal elimination half-life ($t_{½}$), apparent oral clearance (CL/F), and accumulation ratio (R). Descriptive statistics (ie, n, mean, standard deviation [SD], percent coefficient of variation [%CV], minimum, median, maximum, geometric mean [GeoMean], GeoMean %CV, and 95% CIs) will be used to summarize PK parameters for each dose level as appropriate.

3. ECG Assessment:

In Phase 2, the effects of pralsetinib on ECG parameters will be evaluated for 20 patients via 12-lead ECGs extracted from continuous recordings (Holter) on C1D1 and C1D15. Individual ECGs will be extracted in replicate from the Holter recordings at specified time points and will be evaluated by a central laboratory. QT intervals will be measured from Lead II and will be corrected for heart rate (QTc) using Fridericia's correction factors. The primary QTc parameter will be QTcF. Secondary parameters (heart rate, PR and QRS and T-wave morphology) will also be evaluated.

Potential effects of pralsetinib will be evaluated as change from predose baseline heart rate, PR, QRS and QTcF by post-dosing time point. For purposes of QT assessment, exposure-response analysis will be performed of the relationship between pralsetinib plasma levels and ΔQTcF.

4. Pharmacodynamics:

Pharmacokinetic/pharmacodynamic relationships will be descriptively analyzed within each dose level from paired PK/pharmacodynamic sample results for blood (eg, calcitonin, CEA, biochemical response rate, and ctDNA of RET). The change and percent change from baseline will be summarized. Patients' best biochemical response based on calcitonin and CEA will also be summarized, respectively. The detailed definition for categorizing patients into biochemical CR, PR, SD, PD and NE can be found in SAP. Patients with normal serum levels or missing values at baseline will not be included in the analysis.

5. Time to Intracranial Progression and Intracranial Response Rate:

Intracranial progression is defined as ≥ 20% increase along with a 5 mm absolute increase (relative to baseline or nadir on study) in the sum of diameters of any intracranial lesion(s) identified as RECIST v1.1 target lesions at baseline, unequivocal progression of any intracranial lesion(s) identified as RECIST v1.1 nontarget lesions at baseline, or the identification of new intracranial lesion(s).

Time to intracranial progression is defined as the number of weeks from first dose to intracranial progression. Time to intracranial progression will be estimated using the same method for PFS analysis.

Detailed censoring methods will be described in the SAP.

Intracranial response is defined as ≥ 30% decrease in the sum of diameters of any intracranial lesion(s) identified as RECIST v1.1 target lesions at baseline if in the absence of unequivocal progression of any intracranial lesion(s) identified as RECIST v1.1 nontarget lesions at baseline and the identification of new intracranial lesion(s). The intracranial response rate is defined as the proportion of patients who experience intracranial response among those with any intracranial lesion(s) identified as RECIST v1.1 target lesions according to blinded central review at baseline.

The intracranial response rate will be analyzed with the same method used for ORR.

11.4.4 Exploratory Endpoints

11.4.4.1 Phase 1

For all biomarker data, summary statistics and graphs will be provided as appropriate. Relationships between biomarker data and efficacy measures will be descriptively analyzed, as appropriate.

11.4.4.2 Phase 2

For all biomarker data, summary statistics and graphs will be provided as appropriate. Relationships between biomarker data and efficacy measures will be descriptively analyzed, as appropriate.

For EORTC QLQ-C-30 assessments, global health status, functional scales, and symptom scales will be analyzed descriptively at each time point. Change from baseline to each time point will also be analyzed descriptively.

Disease-related symptoms as reported by bowel movement history will be summarized and change from baseline in bowel movement history over time will be analyzed only for RET-mutation MTC.

Time to intracranial progression will be analyzed among all patients with tumor types other than NSCLC; and intracranial response rate will be analyzed among patients with tumor types other than NSCLC; and measurable (target by RECIST v1.1) lesions in the brain (if applicable).

12 ETHICS AND RESPONSIBILITIES

12.1 Good Clinical Practice

The study will be conducted in accordance with the International Conference on Harmonization (ICH) for GCP and the appropriate regulatory requirement(s). The Investigator will be thoroughly familiar with the appropriate use of the study drug as described in the protocol and IB. Essential clinical documents will be maintained to demonstrate the validity of the study and the integrity of the data collected. Master files

should be established at the beginning of the study, maintained for the duration of the study, and retained according to the appropriate regulations.

12.2 Data and Safety Monitoring

This is an open-label, Phase 1/2 study in which all patients receive single-agent pralsetinib. The Sponsor will have access to the safety data on a regular basis. The Sponsor Clinical Study Team will host Investigator teleconferences on a regular basis during the study. Additionally, the Sponsor's Safety Management Team (SMT) will review and manage cumulative safety data at periodic intervals (approximately quarterly) and ad hoc, as needed. The goal of the SMT is to evaluate and detect new safety signals or increased frequency or severity of known safety signals for pralsetinib. The SMT members include representatives from clinical development, clinical pharmacology, non-clinical pharmacology, regulatory affairs, biostatics, clinical operations, and pharmacovigilance.

12.2.1 Phase 1 Data and Safety Monitoring

During the dose-escalation part of the study, the Sponsor Clinical Study Team and the Investigators will meet at the end of each treatment cohort (and at least monthly) to discuss and evaluate all of the gathered safety data. At the dose-escalation teleconference, safety information, including DLTs and all Grade 2 or worse AEs reported during Cycle 1, and all available PK data will be described and reviewed for each patient in the current dose cohort. Updated safety, PK and other data for all other ongoing patients, including data from later cycles, will also be discussed.

Dose-escalation decisions in Phase 1 was based on an evaluation of all relevant, available data, and not solely on DLT information. The selection of the dose for the next cohort of patients was limited to a 100% increase, a 50% increase, or to the addition of 3 patients with no dose increase, as described in Section 6.3.1.2, however, the actual dose level chosen could be less than the maximum permitted, and was based on a medical review of relevant clinical and available PK data. The Sponsor Clinical Study Team and the Investigators must have reached an agreement on whether to determine that the MTD has been achieved, to escalate the dose further, or to de-escalate to a lower dose.

12.2.2 Phase 2 Data and Safety Monitoring

During the expansion part of the study (Phase 2), patient safety will be reviewed on an ongoing basis at a safety review meeting that includes the study investigators and the Sponsor Clinical Study Team. All available safety data will be reviewed to confirm that no unexpected, significant, or unacceptable risks have been discovered. Available PK, pharmacodynamic, and tumor response data will also be reviewed.

12.2.2.1 Phase 2 Safety Review Committee

In the Phase 2 part of the study (dose expansion), a Safety Review Committee (SRC) was established to oversee the safety aspects of the expansion portion of the study. The SRC consisted of, at a minimum, of the Sponsor's Medical Monitor, a Clinical Operations

representative, and at least 4 global pralsetinib Investigators. The Phase 2 safety committee convened at a minimum of every 3 months (or more frequently depending on enrollment or observed safety profile) and perform ongoing review of SAEs and other safety-related data throughout the conduct of the study. Meetings were held by teleconference or in person.

In conjunction with Protocol Amendment 9, a Phase 2 independent data monitoring committee (IDMC) is replacing the Phase 2 SRC.

12.2.2.2 *Phase 2* Independent Data Monitoring Committee

An IDMC has been established in conjunction with Protocol Amendment 9. The roles and responsibilities of the IDMC are listed below. Refer to the IDMC charter for additional information.

The aim of the committee is to safeguard the interests of trial participants, monitor the main outcome measures including safety and efficacy, and monitor the overall conduct of the trial.

The IDMC will receive and review information on the progress and accumulating data of this study and provide advice on the conduct of the study.

The IDMC will make recommendations to the Blueprint Study Management Group and the Study Steering Committee regarding the following aspects of the study, as is relevant in regard to the study design:

a) whether a modification should be made to the study for safety reasons, for example, a modification of the eligibility criteria when the risk/benefit ratio seems unfavorable in specific subgroups of patients.

b) on early termination of a trial when the scientific value of the trial is insufficient, either because of compelling external evidence regarding the hypothesis being tested or because the trial will not be able to produce scientifically valid results due to lack of accrual or lack of quality.

c) on modifications to the study sample size.

d) on actions needed to manage identified issues related to patient compliance or study feasibility and/or quality.

The role of the IDMC is to perform periodic reviews of the study’s progress including adherence to protocol, follow-up assessments, and safety data. Specifically, this role includes:

a) monitoring evidence for treatment harm (eg, toxicity, SAEs, deaths).

b) assessing the impact and relevance of external evidence.

c) deciding whether to recommend that the study continues to recruit participants or whether recruitment should be terminated either for all groups or for some Phase 2 groups and/or some participant subgroups.

d) maintaining confidentiality of all study information that is not in the public domain.

e) considering the ethical implications of any recommendations made by the IDMC.

f) monitoring planned sample size assumptions

g) suggesting additional data analyses if necessary.

h) advising on protocol modifications proposed by the Investigators or the Sponsor (eg, to inclusion criteria, study endpoints, or sample size).

i) monitoring compliance with previous IDMC recommendations

12.3 Institutional Review Board/Independent Ethics Committee

The study will be conducted in accordance with ethical principles founded in the Declaration of Helsinki.

The Investigator must obtain IRB/IEC approval for the investigation and must submit written documentation of the approval to the Sponsor before he or she can enroll any patient into the study. The IRB/IEC will review all appropriate study documentation in order to safeguard the rights, safety, and well-being of the patients. The study will only be conducted at study centers where IRB/IEC approval has been obtained. The protocol, IB, ICF, advertisements (if applicable), written information given to the patients, safety updates, annual progress reports, and any revisions to these documents will be provided to the IRB/IEC. The IRB/IEC is to be notified of any amendment to the protocol in accordance with local requirements. Progress reports and notifications of serious unexpected adverse drug reactions will be provided to the IRB/IEC according to local regulations and guidelines.

12.4 Informed Consent

The Investigator at each study center will ensure that the patient is given full and adequate oral and written information about the nature, purpose, possible risk, and benefit of the study. Patients must also be notified that they are free to discontinue from the study at any time. The patient should be given the opportunity to ask questions and allowed time to consider the information provided.

After the study has been fully explained, written informed consent, including separate consent for exploratory biomarker research to be conducted on biological samples collected during the study, will be obtained from either the patient or his/her guardian or legal representative prior to study participation.

The patient’s signed and dated informed consent must be obtained before conducting any study-related procedures. The Investigator must maintain the original, signed consent form. A copy of the signed form must be given to the patient.

The method of obtaining and documenting the informed consent and the contents of the consent will comply with ICH-GCP and all applicable regulatory requirement(s).

12.5 Records Management

All data for the patients recruited for the trial will be entered onto the eCRFs via an Electronic Data Capture system provided by the Sponsor or designee. Only authorized staff may enter data into the eCRFs. If an entry error is made, the corrections to the eCRFs will be made according to eCRF guidelines by an authorized member of the center staff.

Electronic case report forms will be checked for correctness against source document data by the Sponsor’s monitor. If any entries into the eCRF are incorrect or incomplete, the monitor will ask the Investigator or the study center staff to make appropriate corrections, and the corrected eCRF will again be reviewed for completeness and consistency. Any discrepancies will be noted in the eCRF system by means of electronic data queries. Authorized study center staff will be asked to respond to all electronic queries according to the eCRF guidelines.

12.6 Source Documentation

Source documents/eCRFs will be completed for each study patient. It is the Investigator’s responsibility to ensure the accuracy, completeness, and timeliness of the data reported in the patient’s source document/eCRF. The source document/eCRF should indicate the patient’s participation in the study and should document the dates and details of study procedures, AEs, and patient status.

The Investigator, or designated representative, should complete the source document/eCRF as soon as possible after information is collected, preferably on the same day that a study patient is seen for an examination, treatment, or any other study procedure. Any outstanding entries must be completed immediately after the final examination. An explanation should be given for all missing data.

The Investigator must sign and date the Investigator’s Statement at the end of the source document/eCRF to endorse the recorded data.

The Investigator will retain all completed source documents.

12.7 Study Files and Record Retention

The Investigator will maintain all study records according to ICH-GCP and applicable regulatory requirement(s). Records will be retained for at least 2 years after the last marketing application approval or 2 years after formal discontinuation of the clinical development of the investigational product or according to applicable regulatory

requirement(s). If the Investigator withdraws from the responsibility of keeping the study records, custody must be transferred to a person willing to accept the responsibility. The Sponsor must be notified in writing if a custodial change occurs.

12.8 Liability and Insurance

The Sponsor has subscribed to an insurance policy covering, in its terms and provisions, its legal liability for injuries caused to participating persons and arising out of this research performed strictly in accordance with the scientific protocol as well as with applicable law and professional standards.

13 AUDITING AND MONITORING

The study will be monitored by the Sponsor or its designee. Monitoring will be done by personal visits from a representative of the Sponsor (site monitor) and will include on-site review of the source documents/eCRFs for completeness and clarity, cross-checking with source documents, and clarification of administrative matters will be performed. The review of medical records will be performed in a manner to ensure that patient confidentiality is maintained.

The site monitor will ensure that the investigation is conducted according to protocol design and regulatory requirements by frequent communications.

All unused study drug and other study materials should be destroyed or returned to the Sponsor or designee after the study has been completed, as directed by the Sponsor.

Regulatory authorities, the IRB/IEC, and/or the Sponsor’s clinical quality assurance group or designee may request access to all source documents, eCRFs, and other study documentation for an on-site audit or inspection. Direct access to these documents must be guaranteed by the Investigator, who must provide support at all times for these activities.

14 AMENDMENTS

Protocol modifications, except those intended to reduce immediate risk to study patients, may be made only by Blueprint Medicines. A protocol change intended to eliminate an apparent immediate hazard to patients may be implemented immediately, provided the IRB/IEC is notified within 5 days.

Any permanent change to the protocol must be handled as a protocol amendment. The written amendment must be submitted to the IRB/IEC and the Investigator must await approval before implementing the changes. Blueprint Medicines will submit protocol amendments to the appropriate regulatory authorities for approval.

If in the judgment of the IRB/IEC, the Investigator, and/or Blueprint Medicines, the amendment to the protocol substantially changes the study design and/or increases the potential risk to the patient and/or has an impact on the patient's involvement as a study participant, the currently approved written informed consent form will require similar

modification. In such cases, informed consent will be renewed for patients enrolled in the study before continued participation.

15 STUDY REPORT AND PUBLICATIONS

Blueprint Medicines is responsible for preparing and providing the appropriate regulatory authorities with clinical study reports according to the applicable regulatory requirements.

The publication policy of Blueprint Medicines is discussed in the Investigator's Clinical Research Agreement.

16 STUDY DISCONTINUATION

Both Blueprint Medicines and the Investigator reserve the right to terminate the study at the Investigator’s site at any time. Should this be necessary, Blueprint Medicines or a specified designee will inform the appropriate regulatory authorities of the termination of the study and the reasons for its termination, and the Investigator will inform the IRB/IEC of the same. In terminating the study, Blueprint Medicines and the Principal Investigator will assure that adequate consideration is given to the protection of the patients’ interests.

17 CONFIDENTIALITY

All information generated in this study is considered confidential and must not be disclosed to any person or entity not directly involved with the study unless prior written consent is gained from Blueprint Medicines. However, authorized regulatory officials, IRB/IEC personnel, Blueprint Medicines and its authorized representatives are allowed full access to the records.

Identification of patients and eCRFs shall be by initials (when permitted) and screening and treatment numbers only.

18 REFERENCES

Aaronson NK, Ahmedzai S, Bergman B, Bullinger M, Cull A, Duez NJ, et al. The European Organization for Research and Treatment of Cancer QLQ-C30: a quality-of-life instrument for use in international clinical trials in oncology. *J Natl Cancer Inst.* 1993;85(5):365-76.

Ballerini P, Struski S, Cresson C, Prade N, Toujani S, Deswarte C, et al. RET fusion genes are associated with chronic myelomonocytic leukemia and enhance monocytic differentiation. *Leukemia.* 2012;26(11):2384-9.

Borrello MG, Ardini E, Locati LD, Greco A, Licitra L, and Pierotti MA. RET inhibition: implications in cancer therapy. *Expert Opin Ther Targets.* 2013;17(4):403-19.

Brose MS, Nutting CM, Jarzab B, Elisei R, Siena S, Bastholt L, et al. Sorafenib in radioactive iodine-refractory, locally advanced or metastatic differentiated thyroid cancer: a randomised, double-blind, phase 3 trial. *Lancet.* 2014;384(9940):319-28.

Brown AP, Courtney CL, King LM, Groom SC, and Graziano MJ. Cartilage dysplasia and tissue mineralization in the rat following administration of a FGF receptor tyrosine kinase inhibitor. *Toxicol Pathol.* 2005;33(4):449-55.

Broxmeyer HE. Erythropoietin: multiple targets, actions, and modifying influences for biological and clinical consideration. *J Exp Med.* 2013;210(2):205-8.

Carlomagno F, Guida T, Anaganti S, Vecchio G, Fusco A, Ryan AJ, et al. Disease associated mutations at valine 804 in the RET receptor tyrosine kinase confer resistance to selective kinase inhibitors. *Oncogene.* 2004;23(36):6056-63.

Chen HX, and Cleck JN. Adverse effects of anticancer agents that target the VEGF pathway. *Nat Rev Clin Oncol.* 2009;6(8):465-77.

Coiffier B, Altman A, Pui CH, Younes A, and Cairo MS. Guidelines for the management of pediatric and adult tumor lysis syndrome: an evidence-based review. *J Clin Oncol.* 2008;26(16):2767-78.

CTFG 2014 Recommendations related to contraception and pregnancy testing in clinical trials. Clinical Trials Facilitation Group, http://www.hma.eu/fileadmin/dateien/Human_Medicines/01-About_HMA/Working_Groups/CTFG/2014_09_HMA_CTFG_Contraception.pdf.

Dienstmann R, Rodon J, Prat A, Perez-Garcia J, Adamo B, Felip E, et al. Genomic aberrations in the FGFR pathway: opportunities for targeted therapies in solid tumors. *Ann Oncol.* 2014;25(3):552-63.

Drilon A, Hu ZI, Lai GGY, and Tan DSW. Targeting RET-driven cancers: lessons from evolving preclinical and clinical landscapes. *Nat Rev Clin Oncol.* 2018;15(3):151-167.

Fagin JA, and Wells SA, Jr. Biologic and Clinical Perspectives on Thyroid Cancer. *N Engl J Med.* 2016;375(11):1054-67.

FDA 2016 Drug Development and Drug Interactions: Table of Substrates, Inhibitors, and Inducers. [Online], https://www.fda.gov/drugs/developmentapprovalprocess/developmentresources/druginteractionslabeling/ucm093664.htm.

Fletcher AM, Bregman CL, Woicke J, Salcedo TW, Zidell RH, Janke HE, et al. Incisor degeneration in rats induced by vascular endothelial growth factor/fibroblast growth factor receptor tyrosine kinase inhibition. *Toxicol Pathol.* 2010;38(2):267-79.

Gautschi O, Milia J, Filleron T, Wolf J, Carbone DP, Owen D, et al. Targeting RET in Patients With RET-Rearranged Lung Cancers: Results From the Global, Multicenter RET Registry. *J Clin Oncol.* 2017;35(13):1403-1410.

Horiike A, Takeuchi K, Uenami T, Kawano Y, Tanimoto A, Kaburaki K, et al. Sorafenib treatment for patients with RET fusion-positive non-small cell lung cancer. *Lung Cancer.* 2016;93:43-6.

Klastersky J, de Naurois J, Rolston K, Rapoport B, Maschmeyer G, Aapro M, et al. Management of febrile neutropaenia: ESMO Clinical Practice Guidelines. *Ann Oncol.* 2016;27(suppl 5):v111-v118.

Lin C, Wang S, Xie W, Chang J, and Gan Y. The RET fusion gene and its correlation with demographic and clinicopathological features of non-small cell lung cancer: a meta-analysis. *Cancer Biol Ther.* 2015;16(7):1019-28.

Lin JJ, Kennedy E, Sequist LV, Brastianos PK, Goodwin KE, Stevens S, et al. Clinical Activity of Alectinib in Advanced RET-Rearranged Non-Small Cell Lung Cancer. *J Thorac Oncol.* 2016;11(11):2027-2032.

Liu S, and Yuan Y. Bayesian optimal interval designs for phase I clinical trials. *Journal of the Royal Statistical Society: Series C (Applied Statistics).* 2015;64(3):507-523.

Medico E, Russo M, Picco G, Cancelliere C, Valtorta E, Corti G, et al. The molecular landscape of colorectal cancer cell lines unveils clinically actionable kinase targets. *Nat Commun.* 2015;6:7002.

Mulligan LM. RET revisited: expanding the oncogenic portfolio. *Nat Rev Cancer.* 2014;14(3):173-86.

Nakashima M, Takamura N, Namba H, Saenko V, Meirmanov S, Matsumoto N, et al. RET oncogene amplification in thyroid cancer: correlations with radiation-associated and high-grade malignancy. *Hum Pathol.* 2007;38(4):621-8.

NCCN. NCCN Guidelines®. 2018. Fort Washington, PA: National Comprehensive Cancer Network.

Oken MM, Creech RH, Tormey DC, Horton J, Davis TE, McFadden ET, et al. Toxicity and response criteria of the Eastern Cooperative Oncology Group. *Am J Clin Oncol.* 1982;5(6):649-55.

Parganas E, Wang D, Stravopodis D, Topham DJ, Marine JC, Teglund S, et al. Jak2 is essential for signaling through a variety of cytokine receptors. *Cell.* 1998;93(3):385-95.

Patyna S, Arrigoni C, Terron A, Kim T-W, Heward JK, Vonderfecht SL, et al. Nonclinical Safety Evaluation of Sunitinib: A Potent Inhibitor of VEGF, PDGF, KIT, FLT3, and RET Receptors. *Toxicologic Pathology.* 2008;36(7):905-916.

Platt A, Morten J, Ji Q, Elvin P, Womack C, Su X, et al. A retrospective analysis of RET translocation, gene copy number gain and expression in NSCLC patients treated with vandetanib in four randomized Phase III studies. *BMC Cancer.* 2015;15:171.

Quelle FW, Sato N, Witthuhn BA, Inhorn RC, Eder M, Miyajima A, et al. JAK2 associates with the beta c chain of the receptor for granulocyte-macrophage colony-stimulating factor, and its activation requires the membrane-proximal region. *Mol Cell Biol.* 1994;14(7):4335-41.

Romei C, Ciampi R, Casella F, Tacito A, Torregrossa L, Ugolini C, et al. RET mutation heterogeneity in primary advanced medullary thyroid cancers and their metastases. *Oncotarget.* 2018;9(11):9875-9884.

Romei C, Ciampi R, and Elisei R. A comprehensive overview of the role of the RET proto-oncogene in thyroid carcinoma. *Nat Rev Endocrinol.* 2016;12(4):192-202.

SEER 2018 Surveillance, Epidemiology, and End Results Program. National Cancer Institute, https://seer.cancer.gov/.

Selpercatinib. US Prescribing Information: Available at: https://dailymed.nlm.nih.gov/dailymed/drugInfo.cfm?setid=7fa848ba-a59c-4144-9f52-64d090f4d828

Springuel L, Renauld JC, and Knoops L. JAK kinase targeting in hematologic malignancies: a sinuous pathway from identification of genetic alterations towards clinical indications. *Haematologica.* 2015;100(10):1240-53.

Stransky N, Cerami E, Schalm S, Kim JL, and Lengauer C. The landscape of kinase fusions in cancer. *Nat Commun.* 2014;5:4846.

Wells SA, Jr., Asa SL, Dralle H, Elisei R, Evans DB, Gagel RF, et al. Revised American Thyroid Association guidelines for the management of medullary thyroid carcinoma. *Thyroid.* 2015;25(6):567-610.

Yang HS, and Horten B. Gain of copy number and amplification of the RET gene in lung cancer. *Exp Mol Pathol.* 2014;97(3):465-9.

Yanochko GM, Vitsky A, Heyen JR, Hirakawa B, Lam JL, May J, et al. Pan-FGFR inhibition leads to blockade of FGF23 signaling, soft tissue mineralization, and cardiovascular dysfunction. *Toxicol Sci.* 2013;135(2):451-64.

19 APPENDICES

19.1 Appendix 1: Eastern Cooperative Oncology Group Performance Status

Grade	Symptomatology
0	Fully active, able to carry on all pre-disease performance without restriction
1	Restricted in physically strenuous activity but ambulatory and able to carry out work of a light or sedentary nature (eg, light house work, office work)
2	Ambulatory and capable of all self-care but unable to carry out any work activities. Up and about more than 50% of waking hours
3	Capable of only limited self-care, confined to bed or chair more than 50% of waking hours
4	Completely disabled. Cannot carry on any self-care. Totally confined to bed or chair
5	Dead

Source: (Oken et al, 1982).

19.2 Appendix 2: List of Prohibited Medications and Food

Strong Inhibitors of CYP3A4	Strong Inducers of CYP3A4
Boceprevir	Carbamazepine
Clarithromycin	Phenobarbital
Cobicistat	Phenytoin
Conivaptan	Rifampin
Grapefruit, Grapefruit juice	St. John's Wort
Indinavir	
Itraconazole	
Ketoconazole	
Lopinavir	
Nefazodone	
Nelfinavir	
Posaconazole	
Ritonavir	
Saquinavir	
Telaprevir	
Telithromycin	
Voriconazole	
**Strong Dual P-gp and CYP3A4 Inhibitors:**	
Mifebradil	
Itraconazole	

Source: (FDA, 2016)
This list is not intended to be exhaustive. A similar restriction will apply to other drugs that fall in these categories; appropriate medical judgment is required. Please contact Blueprint Medicines with any queries relating to this issue.

19.3 Appendix 3: List of Medications and Food to be Used with Caution

Sensitive CYP3A4 Substrates	Sensitive CYP2C9 Substrates	Sensitive CYP2C8 Substrates	Sensitive P-gp Substrates	Sensitive P-gp Substrates	Sensitive OATP1B1, OATP1B3 Substrates	Sensitive OAT1 Substrates	Sensitive MATE1, MATE2-K Substrates
Alfentanil	Warfarin - with INR monitoring	Repaglinide	Digoxin	2-amino-1-methyl-6-phenylimidazo[4,5-b]pyridine (PhIP)	Cholecystokinin octapeptide(CCK-8)	Adefovir	Metformin
Cyclosporine	Celecoxib		Fexofenadine	Coumestrol	Estradiol-17β-glucuronide	*p*-aminohippurate	1-methyl-4-phenylpyridinium (MPP+)
Dihydroergotamine			Loperamide	Daidzein	Estrone-3-sulfate	Cidofovir	Tetraethylammonium (TEA)
Ergotamine			Quinidine	Dantrolene	Pitavastatin	Tenofovir	
Fentanyl			Talinolol	Estrone-3-sulfate	Pravastatin		
Midazolam			Vinblastine	Genistein	Telmisartan		
Pimozide				Prazosin	Rosuvastatin		
Quinidine				Sulfasalazine			
Simvastatin							
Sirolimus							
Tacrolimus							
Terfenadine							

Source: (FDA, 2016)

This list is not intended to be exhaustive. A similar restriction will apply to other drugs that fall in these categories; appropriate medical judgment is required. Please contact Blueprint Medicines with any queries relating to this issue.

19.4 Appendix 4: Operating Characteristics of the BOIN Design

OPERATING CHARACTERISTICS OF THE BOIN DESIGN

0	Dose Level													Number of Patients	% Early Stopping
0	**1**	**2**	**3**	**4**	**5**	**6**	**7**	**8**	**9**	**10**	**11**	**12**	**13**		
**Scenario 1**															
**True DLT rate**	0.02	0.30	0.90	0.99	1.00	1.00	1.00	1.00	1.00	1.00	1.00	1.00	1.00	18.80	0.0
**Selection %**	13.2	86.8	0.0	0.0	0.0	0.0	0.0	0.0	0.0	0.0	0.0	0.0	0.0		
**# Pts Treated**	5.5	11.2	2.1	0.0	0.0	0.0	0.0	0.0	0.0	0.0	0.0	0.0	0.0		
**Scenario 2**															
**True DLT rate**	0.02	0.09	0.30	0.66	0.90	0.98	0.99	1.00	1.00	1.00	1.00	1.00	1.00	23.24	0.0
**Selection %**	0.2	19.1	77.5	3.1	0.0	0.0	0.0	0.0	0.0	0.0	0.0	0.0	0.0		
**# Pts Treated**	3.3	6.2	10.4	3.2	0.1	0.0	0.0	0.0	0.0	0.0	0.0	0.0	0.0		
**Scenario 3**															
**True DLT rate**	0.02	0.05	0.13	0.30	0.54	0.77	0.90	0.96	0.99	0.99	1.00	1.00	1.00	27.07	0.0
**Selection %**	0.1	1.4	24.2	63.8	10.5	0.0	0.0	0.0	0.0	0.0	0.0	0.0	0.0		
**# Pts Treated**	3.2	3.8	6.6	9.3	3.9	0.3	0.0	0.0	0.0	0.0	0.0	0.0	0.0		
**Scenario 4**															
**True DLT rate**	0.02	0.04	0.09	0.17	0.30	0.48	0.66	0.81	0.90	0.95	0.98	0.99	0.99	30.50	0.0

0	Dose Level													Number of Patients	% Early Stopping
0	**1**	**2**	**3**	**4**	**5**	**6**	**7**	**8**	**9**	**10**	**11**	**12**	**13**		
**Selection %**	0.1	0.5	5.4	28.4	50.9	14.4	0.4	0.0	0.0	0.0	0.0	0.0	0.0		
**# Pts Treated**	3.2	3.5	4.6	6.8	7.9	3.9	0.6	0.0	0.0	0.0	0.0	0.0	0.0		
**Scenario 5**															
**True DLT rate**	0.02	0.04	0.06	0.11	0.19	0.30	0.44	0.59	0.73	0.83	0.90	0.94	0.97	33.89	0.0
**Selection %**	0.1	0.4	1.4	8.4	29.4	42.5	16.7	1.2	0.0	0.0	0.0	0.0	0.0		
**# Pts Treated**	3.2	3.4	3.8	4.9	6.8	7.1	3.8	0.8	0.1	0.0	0.0	0.0	0.0		
**Scenario 6**															
**True DLT rate**	0.02	0.03	0.05	0.09	0.13	0.21	0.30	0.42	0.54	0.66	0.77	0.84	0.90	36.93	0.0
**Selection %**	0.0	0.2	0.8	3.7	11.9	29.5	35.8	15.9	2.1	0.1	0.0	0.0	0.0		
**# Pts Treated**	3.2	3.3	3.6	4.3	5.3	6.6	6.2	3.4	0.9	0.1	0.0	0.0	0.0		
**Scenario 7**															
**True DLT rate**	0.02	0.03	0.05	0.07	0.10	0.15	0.22	0.30	0.40	0.51	0.61	0.71	0.79	40.06	0.0
**Selection %**	0.0	0.2	0.6	1.8	5.2	14.8	28.3	30.6	15.4	2.8	0.2	0.0	0.0		
**# Pts Treated**	3.2	3.3	3.6	3.9	4.5	5.5	6.3	5.6	3.2	1.0	0.1	0.0	0.0		

0	Dose Level													Number of Patients	% Early Stopping
0	**1**	**2**	**3**	**4**	**5**	**6**	**7**	**8**	**9**	**10**	**11**	**12**	**13**		
**Scenario 8**															
**True DLT rate**	0.02	0.03	0.04	0.06	0.09	0.12	0.17	0.23	0.30	0.39	0.48	0.57	0.66	42.89	0.0
**Selection %**	0.1	0.1	0.4	1.2	3.4	7.8	17.0	26.3	26.6	13.7	3.2	0.3	0.0		
**# Pts Treated**	3.2	3.3	3.5	3.7	4.2	4.8	5.5	5.8	4.9	2.8	0.9	0.2	0.0		
**Scenario 9**															
**True DLT rate**	0.02	0.03	0.04	0.05	0.07	0.10	0.13	0.18	0.23	0.30	0.38	0.46	0.54	45.95	0.0
**Selection %**	0.0	0.2	0.3	0.6	1.8	4.4	9.5	17.4	24.5	24.2	13.1	3.7	0.5		
**# Pts Treated**	3.2	3.3	3.4	3.6	3.9	4.3	4.9	5.4	5.5	4.6	2.7	1.0	0.2		
**Scenario 10**															
**True DLT rate**	0.02	0.03	0.04	0.05	0.06	0.09	0.11	0.15	0.19	0.24	0.30	0.37	0.44	48.47	0.0
**Selection %**	0.0	0.1	0.3	0.6	1.2	3.1	5.8	11.7	17.9	22.6	20.6	11.6	4.3		
**# Pts Treated**	3.2	3.3	3.4	3.6	3.7	4.1	4.5	5.0	5.2	5.0	4.0	2.4	1.0		

Abbreviations: BOIN = Bayesian optimal interval, DLT = dose-limiting toxicity, pts = patients

19.5 Appendix 5: Investigator Signature Page

BLU-667-1101

A Phase 1/2 Study of the Highly-selective RET inhibitor, BLU-667, in Patients with Thyroid Cancer, Non-Small Cell Lung Cancer (NSCLC) and other Advanced Solid Tumors

CONFIDENTIALITY AND INVESTIGATOR STATEMENT

The information contained in this protocol and all other information relevant to pralsetinib are the confidential and proprietary information of Blueprint Medicines, and except as may be required by federal, state or local laws or regulation, may not be disclosed to others without prior written permission of Blueprint Medicines.

I have read the protocol, including all appendices, and I agree that it contains all of the necessary information for me and my staff to conduct this study as described. I will conduct this study as outlined herein, in accordance with the regulations stated in the Federal Code of Regulations for Good Clinical Practices and International Conference on Harmonization guidelines, and will make a reasonable effort to complete the study within the time designated.

I will provide all study personnel under my supervision copies of the protocol and any amendments, and access to all information provided by Blueprint Medicines or specified designees. I will discuss the material with them to ensure that they are fully informed about pralsetinib and the study.

Principal Investigator Name (printed)　　　Signature

Date

19.6 Appendix 6: Common Oncogenic RET Mutations

COMMON ONCOGENIC RET MUTATIONS

RET mutation	Exon
G533C	8
C609F/G/R/S/Y	10
C611F/G/S/Y/W	10
C618F/R/S	10
C620F/R/S	10
C630R/Y	11
D631Y	11
C634F/G/R/S/W/Y	11
K666E	11
E768D	13
L790F	13
V804L	14
V804M	14
A883F	15
S891A	15
R912P	16
M918T	16

Source: (Wells et al, 2015)

19.7 Appendix 7: EORTC QLQ-C30 Assessment

[redacted]

[redacted]

19.8 Appendix 8: Examples of Implementing the Early Stopping Rule

Number of Patients Discontinued	Number of Patients Treated	70% Exact Binomial CI, 1-sided lower bound	Action
23	140	14.57%	No Stop
24	140	15.25%	Stop
25	150	14.87%	No Stop
26	150	15.51%	Stop
26	160	14.53%	No Stop
27	160	15.13%	Stop
28	170	14.8%	No Stop
29	170	15.36%	Stop
29	180	14.51%	No Stop
30	180	15.04%	Stop
31	190	14.75%	No Stop
32	190	15.26%	Stop
33	200	14.97%	No Stop
34	200	15.45%	Stop
34	210	14.71%	No Stop
35	210	15.17%	Stop
36	220	14.92%	No Stop
37	220	15.35%	Stop
37	230	14.68%	No Stop
38	230	15.1%	Stop
39	240	14.87%	No Stop
40	240	15.28%	Stop
40	250	14.66%	No Stop
41	250	15.05%	Stop
42	260	14.84%	No Stop
43	260	15.21%	Stop
43	270	14.64%	No Stop
44	270	15%	No Stop
45	280	14.81%	No Stop
46	280	15.16%	Stop
47	290	14.97%	No Stop
48	290	15.3%	Stop
48	300	14.79%	No Stop

Number of Patients Discontinued	Number of Patients Treated	70% Exact Binomial CI, 1-sided lower bound	Action
49	300	15.11%	Stop
50	310	14.93%	No Stop
51	310	15.25%	Stop
51	320	14.77%	No Stop
52	320	15.07%	Stop
53	330	14.91%	No Stop
54	330	15.2%	Stop
54	340	14.75%	No Stop
55	340	15.04%	Stop
56	350	14.88%	No Stop
57	350	15.16%	Stop
57	360	14.74%	No Stop
58	360	15.01%	Stop
59	370	14.86%	No Stop
60	370	15.13%	Stop
61	380	14.98%	No Stop
62	380	15.24%	Stop
62	390	14.85%	No Stop
63	390	15.1%	Stop
64	400	14.96%	No Stop
65	400	15.2%	Stop
65	410	14.83%	No Stop
66	410	15.07%	Stop
67	420	14.94%	No Stop
68	420	15.17%	Stop
68	430	14.82%	No Stop
69	430	15.05%	Stop
70	440	14.92%	No Stop
71	440	15.15%	Stop
71	450	14.81%	No Stop
72	450	15.02%	Stop
73	460	14.91%	No Stop
74	460	15.12%	Stop

Number of Patients Discontinued	Number of Patients Treated	70% Exact Binomial CI, 1-sided lower bound	Action
74	470	14.8%	No Stop
75	470	15.01%	Stop
76	480	14.89%	No Stop
77	480	15.1%	Stop
78	490	14.99%	No Stop
79	490	15.19%	Stop
79	500	14.88%	No Stop
80	500	15.08%	Stop
81	510	14.97%	No Stop
82	510	15.16%	Stop
82	520	14.87%	No Stop
83	520	15.06%	Stop
84	530	14.96%	No Stop
85	530	15.14%	Stop
85	540	14.86%	No Stop
86	540	15.04%	Stop
87	550	14.95%	No Stop
88	550	15.12%	Stop
88	560	14.85%	No Stop
89	560	15.03%	Stop
90	570	14.93%	No Stop
91	570	15.11%	Stop
92	580	15.01%	Stop
93	590	14.92%	No Stop
94	590	15.09%	Stop
95	600	15%	No Stop
96	600	15.16%	Stop
96	610	14.91%	No Stop
97	610	15.07%	Stop
98	620	14.99%	No Stop
99	620	15.15%	Stop
99	630	14.9%	No Stop
100	630	15.06%	Stop

Number of Patients Discontinued	Number of Patients Treated	70% Exact Binomial CI, 1-sided lower bound	Action
101	640	14.98%	No Stop
102	640	15.13%	Stop
102	650	14.9%	No Stop
104	660	14.97%	No Stop
105	660	15.12%	Stop
105	670	14.89%	No Stop
106	670	15.03%	Stop

Abbreviations: CI = confidence interval

Supplementary Table 1 | Study Sites Whose IEC/IRB Approved the Protocol

Investigator	Study Site
Martin Schuler	University Hospital Essen, Essen, Germany
Hans Prenen	Oncology Department, Antwerp University Hospital, Antwerp, Belgium
Elena Garralda	Vall d'Hebron Institute of Oncology, Vall d'Hebron Hospital, Barcelona, Spain
Luis Paz-Ares	Hospital Universitario 12 de Octubre, CNIO-H12o Lung Cancer Unit, Ciberonc and Complutense University, Madrid, Spain
Pilar Garrido	IRYCIS Hospital Universitario Ramón y Cajal, Ciberonc and Alcalá University, Madrid, Spain
Ernest Nadal	Catalan Institute of Oncology, L'Hospitalet, Barcelona, Spain
Phillipe Cassier	Centre Léon Bérard, Lyon, France
Debashis Sarker	Guy's and St Thomas' NHS Foundation Trust - Guy's Hospital, London, UK
Giuseppe Curigliano	Istituto Europeo di Oncologia, IRCCS, Milan, Italy
Salvatore Siena	Niguarda Cancer Center, ASST Grande Ospedale Metropolitano Niguarda, Milan, Italy
Paolo Marchetti	Istituto Nazionale Tumori Regina Elena, Rome, Italy
Adrianus Joop de Langen	Nederlands Kanker Instituut – Antoni van Leeuwenhoek Ziekenhuis, Amsterdam, Netherlands
Jacqueline Vuky	Oregon Health & Science University, Portland, OR, USA

Gilberto Lopes	Sylvester Comprehensive Cancer Center, Miami, FL, USA
Shirish Gadgeel	University of Michigan, Ann Arbor, MI, USA
Yariv Houvras	NewYork-Presbyterian Hospital/Weill Cornell Medical Center, New York, NY, USA
Robert Doebele	University of Colorado Anschutz Medical Campus, Aurora, CO, USA
Helen Ross	Mayo Clinic Hospital, Phoenix, AZ, USA
Byong Chul Cho	Severance Hospital - Yonsei Cancer Center, Seoul, South Korea
Myung-Ju Ahn	Samsung Medical Center, Seoul, South Korea
Yun Fan	Department of Medical Oncology, Zhejiang Cancer Hospital, Hangzhou, China
Jianhua Chang	Fudan University Shanghai Cancer Center, Shanghai, China
Vivek Subbiah	The University of Texas MD Anderson Cancer Center, Houston, TX, USA
