# Supplementary material for: Pan-cancer efficacy of pralsetinib in patients with RET fusion–positive solid tumors from the phase 1/2 ARROW trial
Source: Nat Med. 2022 Aug 12;28(8):1640–5. doi: 10.1038/s41591-022-01931-y (PMC9388374; doi:10.1038/s41591-022-01931-y)
Supplement: Supplementary file 2 — Reporting Summary [file 41591_2022_1931_MOESM2_ESM.pdf]

nature portfolio

Corresponding author(s): Vivek Subbiah

Last updated by author(s): Jun 17, 2022



# Reporting Summary

Nature Portfolio wishes to improve the reproducibility of the work that we publish. This form provides structure for consistency and transparency in reporting. For further information on Nature Portfolio policies, see our Editorial Policies and the Editorial Policy Checklist.

## Statistics

For all statistical analyses, confirm that the following items are present in the figure legend, table legend, main text, or Methods section.

| n/a | Confirmed | |
|---|---|---|
| ☐ | ☒ | The exact sample size (*n*) for each experimental group/condition, given as a discrete number and unit of measurement |
| ☒ | ☐ | A statement on whether measurements were taken from distinct samples or whether the same sample was measured repeatedly |
| ☐ | ☒ | The statistical test(s) used AND whether they are one- or two-sided<br>*Only common tests should be described solely by name; describe more complex techniques in the Methods section.* |
| ☒ | ☐ | A description of all covariates tested |
| ☒ | ☐ | A description of any assumptions or corrections, such as tests of normality and adjustment for multiple comparisons |
| ☐ | ☒ | A full description of the statistical parameters including central tendency (e.g. means) or other basic estimates (e.g. regression coefficient) AND variation (e.g. standard deviation) or associated estimates of uncertainty (e.g. confidence intervals) |
| ☒ | ☐ | For null hypothesis testing, the test statistic (e.g. $F$, $t$, $r$) with confidence intervals, effect sizes, degrees of freedom and $P$ value noted<br>*Give P values as exact values whenever suitable.* |
| ☒ | ☐ | For Bayesian analysis, information on the choice of priors and Markov chain Monte Carlo settings |
| ☒ | ☐ | For hierarchical and complex designs, identification of the appropriate level for tests and full reporting of outcomes |
| ☒ | ☐ | Estimates of effect sizes (e.g. Cohen's $d$, Pearson's $r$), indicating how they were calculated |

*Our web collection on statistics for biologists contains articles on many of the points above.*

## Software and code

Policy information about availability of computer code

| | |
|---|---|
| Data collection | No software was used. |
| Data analysis | SAS version 9.4 |

For manuscripts utilizing custom algorithms or software that are central to the research but not yet described in published literature, software must be made available to editors and reviewers. We strongly encourage code deposition in a community repository (e.g. GitHub). See the Nature Portfolio guidelines for submitting code & software for further information.

## Data

Policy information about availability of data

All manuscripts must include a data availability statement. This statement should provide the following information, where applicable:

- Accession codes, unique identifiers, or web links for publicly available datasets
- A description of any restrictions on data availability
- For clinical datasets or third party data, please ensure that the statement adheres to our policy

The anonymized derived data from the registrational ARROW study (NCT03037385) that underlie the results reported in this article may be made available after Roche and/or Blueprint Medicines have received regulatory approval for pralsetinib in the US and the EU in the tumour-agnostic setting described herein, or upon terminating its clinical development in this setting. Qualified researchers can then request access to individual patient level clinical data through a data request platform. At the time of writing this request the platform is Vivli (https://vivli.org/ourmember/roche/). As RET fusions are rare alterations, the anonymization of patient level data in patient subgroups or trial cohorts of fewer than 50 patients may be difficult to achieve. As a result, Roche will assess the feasibility of anonymization and therefore data release as part of the review of enquiries. For up to date details on Roche's Global Policy on the Sharing of Clinical Information and how to request access to related clinical study documents, see here: https://go.roche.com/data_sharing.

Consistent with expectations of good scientific practice, researchers can request access to our studies by providing a data request with a commitment to publish their findings. The data request is reviewed by an independent review panel. This is a team of external independent experts who were appointed by the Wellcome Trust. Considering the commitment to publish, i.e. releasing some of the data obtained through the data request into the public domain, the following considerations apply:

• A legitimate commercial interest in light of the data being part of a registrational study where data have not yet been submitted to regulatory agencies for review (Roche releases data after the medicine studied has been approved by regulators for the indication in both the US and EU or terminated from development (all indications) and usually 18 months after completion of the study report (to enable a publication to be submitted). It would for example be in our interest to publish the regulatory filing data set prior to making data available to researchers.

• Patient confidentiality: it needs to be considered whether the data can be properly anonymized in light of the rarity of the alteration and the low patient numbers so that the privacy and confidentiality of research participants is not compromised in any way.

# Field-specific reporting

Please select the one below that is the best fit for your research. If you are not sure, read the appropriate sections before making your selection.

☒ Life sciences ☐ Behavioural & social sciences ☐ Ecological, evolutionary & environmental sciences

For a reference copy of the document with all sections, see nature.com/documents/nr-reporting-summary-flat.pdf

# Life sciences study design

All studies must disclose on these points even when the disclosure is negative.

| | |
|---|---|
| Sample size | 587 patients were enrolled across all groups by the data cut-off. For Group 5, which excluded patients with RET fusion–positive NSCLC but included patients with RET fusion–positive thyroid cancer, a total sample size of 100 patients with solid tumors harboring a RET fusion was intended to allow >90% power at the 2-sided significance level of 0.05 for testing the assumption of null hypothesis of ORR = 0.1 versus the alternative ORR = 0.3. Findings for patients with RET fusion–positive thyroid cancer have been reported previously so were excluded from this interim analysis. |
| Data exclusions | 3 patients were excluded from the efficacy evaluable population due to additional oncogenic driver mutations in addition to RET. This was a prespecified exclusion criterion. |
| Replication | N/A - This was an interim analysis of a non-randomized Phase 1/2 clinical study in a subset of patients from the ARROW trial with RET fusion-positive solid tumors. |
| Randomization | This is a single-arm study with no randomization. |
| Blinding | This is a single-arm study with no blinding. |

# Reporting for specific materials, systems and methods

We require information from authors about some types of materials, experimental systems and methods used in many studies. Here, indicate whether each material, system or method listed is relevant to your study. If you are not sure if a list item applies to your research, read the appropriate section before selecting a response.

## Materials & experimental systems

| n/a | Involved in the study | |
|---|---|---|
| ☒ | ☐ | Antibodies |
| ☒ | ☐ | Eukaryotic cell lines |
| ☒ | ☐ | Palaeontology and archaeology |
| ☒ | ☐ | Animals and other organisms |
| ☐ | ☒ | Human research participants |
| ☐ | ☒ | Clinical data |
| ☒ | ☐ | Dual use research of concern |

## Methods

| n/a | Involved in the study | |
|---|---|---|
| ☒ | ☐ | ChIP-seq |
| ☒ | ☐ | Flow cytometry |
| ☒ | ☐ | MRI-based neuroimaging |

## Human research participants

Policy information about studies involving human research participants

| | |
|---|---|
| Population characteristics | N/A - no covariate analyses conducted. The median age (range) of the response-evaluable population was 53 years (31-71), with 14 (61%) female patients. Sixty-five percent were white, 30% were asian, and 4% were black. |
| Recruitment | Patients were recruited by participating investigators. The Investigator at each center ensured that the patients were given |

| | |
|---|---|
| Recruitment | full and adequate oral and written information about the nature, purpose, possible risk, and benefit of the study. Patients were also notified that they were free to discontinue from the study at any time. Patients were given the opportunity to ask questions and allowed time to consider the information provided. Patients were recruited strictly abiding to the inclusion and exclusion criteria which are defined in full in the manuscript Supplementary Information (Protocol). All patients provided written informed consent. Participants were not compensated, except for the reimbursement of reasonable travel expenses.<br><br>Patients were enrolled across 23 study centers in 10 countries worldwide. Due to the geographical distribution of the study centers, participants may not represent the global general population. No other bias emerging from recruitment is expected. |
| Ethics oversight | The full protocol was approved by the institutional review board or independent ethics committee of each participating site. The name of each participating institute/organization/site in this study whose ethical committee approved the protocol is provided in the supplementary information. |

Note that full information on the approval of the study protocol must also be provided in the manuscript.

# Clinical data

Policy information about clinical studies

All manuscripts should comply with the ICMJE guidelines for publication of clinical research and a completed CONSORT checklist must be included with all submissions.

| | |
|---|---|
| Clinical trial registration | NCT03037385 |
| Study protocol | Provided with submission |
| Data collection | Patients recruited and data collected at hospitals and medical centers between March 2017 and November 2020 (data cut-off) |
| Outcomes | Primary endpoints were objective tumor response rate (confirmed complete response [CR] or partial response [PR]) per Response Evaluation Criteria in Solid Tumors version 1.1) and safety. Key secondary endpoints included clinical benefit rate, disease control rate, duration of response, progression-free survival, and overall survival. Tumor response per RECIST v1.1 was assessed by blinded independent central review (BICR). Computed tomography or magnetic resonance imaging of all known disease sites was performed at screening and approximately every 8 weeks during treatment. Adverse events were graded according to the US National Cancer Institute Common Terminology Criteria for Adverse Events version 4.03 and terms were pooled. DOR, PFS, and OS were analyzed using the Kaplan–Meier method. Estimates of follow-up duration for DOR, PFS, and OS were based on the inverse Kaplan–Meier method with 95% CIs based on the Greenwood formula. |
